# A hypothalamic circuit for circadian regulation of corticosterone secretion

**DOI:** 10.21203/rs.3.rs-4718850/v1

**Published:** 2024-07-12

**Authors:** Oscar D. Ramirez-Plascencia, Roberto De Luca, Natalia L. S. Machado, Dominique Eghlidi, Mudasir A. Khanday, Sathyajit S. Bandaru, Francesca Raffin, Nina Vujovic, Elda Arrigoni, Clifford B. Saper

**Affiliations:** 1. Department of Neurology, Division of Sleep Medicine, Beth Israel Deaconess Medical Center, Boston, MA 02215, USA.; 2. Division of Sleep Medicine, Harvard Medical School, Boston, MA 02215, USA.; 3. Departments of Medicine and Neurology, Brigham and Women’s Hospital, Boston, MA 02115, USA.; 4. Department of Biology and Biotechnology “Lazzaro Spallanzani”, University of Pavia, Pavia, PV 27100, Italy.

**Keywords:** corticosterone, corticotropin-releasing hormone, dorsomedial hypothalamus and paraventricular hypothalamus

## Abstract

The secretion of cortisol in humans and corticosterone (Cort) in rodents follows a daily rhythm which is important in readying the individual for the daily active cycle and is impaired in chronic depression. This rhythm is orchestrated by the suprachiasmatic nucleus (SCN) which governs the activity of neurons in the paraventricular nucleus of the hypothalamus that produce the corticotropin-releasing hormone (PVH^CRH^ neurons). The dorsomedial nucleus of the hypothalamus (DMH) serves as a crucial intermediary, being innervated by the SCN both directly and via relays in the subparaventricular zone, and projecting axons to the PVH, thereby exerting influence over the cortisol/corticosterone rhythm. However, the role and synaptic mechanisms by which DMH neurons regulate the daily rhythm of Cort secretion has not been explored. We found that either ablating or acutely inhibiting the DMH glutamatergic (DMH^Vglut2^) neurons resulted in a 40–70% reduction in the daily peak of Cort. Deletion of the *Vglut2* gene within the DMH produced a similar effect, highlighting the indispensable role of glutamatergic signaling. Chemogenetic stimulation of DMH^Vglut2^ neurons led to an increase of Cort levels, and optogenetic activation of their terminals in the PVH in hypothalamic slices directly activated PVH^CRH^ neurons through glutamate release (the DMH^Vglut2^ → PVH^CRH^ pathway). Similarly, ablating, inhibiting, or disrupting GABA transmission by DMH GABAergic (DMH^Vgat^) neurons diminished the circadian peak of Cort, particularly under constant darkness conditions. Chemogenetic stimulation of DMH^Vgat^ neurons increased Cort, although with a lower magnitude compared to DMH^Vglut2^ neuron stimulation, suggesting a role in disinhibiting PVH^CRH^ neurons. Supporting this hypothesis, we found that rostral DMH^Vgat^ neurons project directly to GABAergic neurons in the caudal ventral part of the PVH and adjacent peri-PVH area (cvPVH), which directly inhibit PVH^CRH^ neurons, and that activating the DMH^Vgat^ terminals in the cvPVH in brain slices reduced GABAergic afferent input onto the PVH^CRH^ neurons. Finally, ablation of cvPVH^Vgat^ neurons resulted in increased Cort release at the onset of the active phase, affirming the pivotal role of the DMH^Vgat^ → cvPVH^Vgat^ → PVH^CRH^ pathway in Cort secretion. In summary, our study delineates two parallel pathways transmitting temporal information to PVH^CRH^ neurons, collectively orchestrating the daily surge in Cort in anticipation of the active phase. These findings are crucial to understand the neural circuits regulating Cort secretion, shedding light on the mechanisms governing this physiological process and the coordinated interplay between SCN, DMH, and PVH.

## Graphical Abstract

*Model Proposed for the hypothalamic circuitry involved in the circadian regulation of Cort release.* During the first part of the light period, the DMH^Vglut2^ and DMH^Vgat^ neurons are inhibited by the SCN, allowing a tonic inhibition of the PVH^CRH^ neurons from the cvPVH^Vgat^ neurons (left panel). Starting the second part of the light phase thought the first hours of dark period, the Cort levels gradually increases by direct DMH^Vglut2^ → PVH^CRH^, and a parallel DMH^Vgat^ → cvPVH^Vgat^ → PVH^CRH^ disinhibitory circuit (right panel).

## Introduction

PVH^CRH^ neurons play a key role in the secretion of corticosteroids by releasing CRH into the hypothalamic-hypophysial portal circulation to stimulate the anterior pituitary release of adrenocorticotropic hormone (ACTH). ACTH then stimulates the adrenal cortex to secrete cortisol in humans and corticosterone (Cort) in rodents. The secretion of Cort follows a daily rhythm, with peak levels typically occurring just before the onset of the active phase, thereby regulating behavior, metabolism, and immune response. Trough levels occur toward the onset of the sleep phase, reflecting the natural dip in hormone secretion during the body’s resting period ^1,2^. This rhythmic secretion pattern reflects the intricate coordination of various neural and hormonal pathways involved in circadian regulation. Loss of the circadian rhythm of Cort secretion in rats after lesions of the suprachiasmatic nucleus (SCN) provided some of the first evidence for the role of the SCN as the brain’s master biological clock ^3^. However, the mechanism by which the SCN influences Cort secretion remains unknown. The SCN sends only limited axons to the PVH, where they mainly terminate in subregions of the PVH that control autonomic activity ^4,5^ and spare the PVH^CRH^ neurons. Indeed, the bulk of SCN efferents terminate in an arc stretching caudally and dorsally from the SCN, encompassing the subparaventricular zone (SPZ) and dorsomedial nucleus of the hypothalamus (DMH). ^6,7^. In addition, non-specific ablation of DMH neurons in rats eliminates the daily peak in Cort secretion, reducing the overall daily levels ^8^. Stimulation of the DMH increases ACTH and Cort release ^9,10^. These findings led us to hypothesize that the daily peak in Cort could be regulated by the SCN through the DMH, that, in turn, stimulates the PVH^CRH^ neurons to trigger Cort release ^11^. As the DMH is a heterogeneous region containing roughly equal proportions of glutamatergic and GABAergic neurons, we examined the roles of both GABAergic and glutamatergic neurons of the DMH in the regulation of Cort levels via the PVH^CRH^ neurons.

First, we tested whether the DMH^Vglut2^ neurons were necessary for the circadian regulation of Cort by either ablating or inhibiting them or deleting the *Vglut2* gene in the DMH, preventing glutamate release. We then tested the effect of chemogenetic activation of DMH^Vglut2^ neurons on Cort levels and used Channelrhodopsin-assisted circuit mapping (CRACM) to determine the synaptic effects of DMH^Vglut2^ → PVH^CRH^ input. We also assessed the effect of the activation, as well as ablation or inhibition of the DMH^Vgat^ neurons, and whether deleting the *Vgat* gene in the DMH affected the daily rhythm of Cort release. Finally, we examined the role of the caudal ventral part of the PVH and adjacent peri-PVH area GABAergic neurons (cvPVH^Vgat^) in disinhibiting the PVH^CRH^ neurons through a polysynaptic circuit (DMH^Vgat^→ cvPVH^Vgat^→ PVH^CRH^).

## Results

### Glutamate signaling by DMH^Vglut2^ neurons is necessary for the increase in Cort at the beginning of the active period.

**Figure 1.**
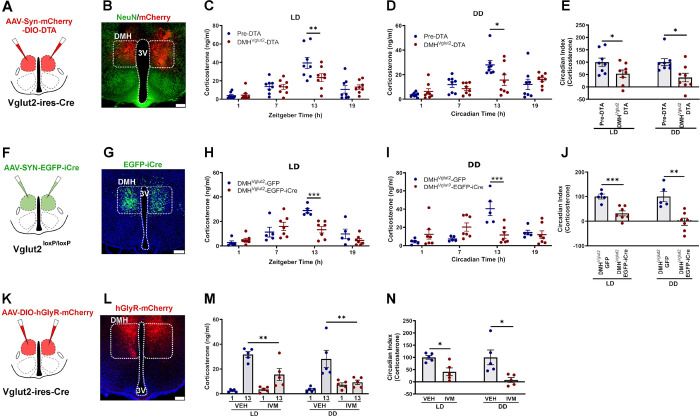
DMH^Vglut2^ neurons are necessary to maintain the endogenous circadian rhythm of Cort release. (**A**) Schematic of the DMH^Vglut2^ neuron ablation by DTA (**B**) Representative image of a section from an animal with DMH^Vglut2^ neuron ablation, showing the injection site in red (non-DMH^Vglut2^ neurons expressing mCherry) and green NeuN staining as a reference marker. (**C**) Ablation of DMH^Vglut2^ neurons reduces the circadian peak of Cort at ZT13 in LD (*Two-way ANOVA*; *Tukey’s* multiple comparisons test. ZT13 Pre-DTA vs DMH^Vglut2^-DTA: **p=0.007) and (**D**) in DD at CT13 (*Two-way ANOVA*; *Tukey’s* multiple comparisons test. CT13 Pre-DTA vs DMH^Vglut2^-DTA: *p=0.013). (**E**) The Cort circadian index (CI) in the DMH^Vglut2^ ablated mice is reduced by 46.6 ±14.2% in LD (*Paired t-test*: t=2.225, df=14, *p=0.043) and 62.0 ±17.3% in DD (*Unpaired* t-test: t=2.775, df=14, *p=0.014). (**F**) Schematic representation of *Vglut2* gene deletion in the DMH. (**G**) Representative micrograph showing EGFP expression in the DMH neurons in which the *Vglut2* gene has been deleted (in green). (**H**) *Vglut2* deletion in DMH diminishes the Cort peak at ZT13 in LD (*Two-way ANOVA*; *Tukey’s* multiple comparisons test. ZT13 DMH^Vglut2^-GFP vs DMH^Vglut2^-EGFP-iCre: ***p<0.001) and entirely prevents the peak at CT13 in DD (**I**; *Two-way ANOVA*; *Tukey’s* multiple comparisons test. CT13 DMH^Vglut2^-GFP vs DMH^Vglut2^-EGFP-iCre: ***p<0.001). (**J**) The Cort CI is reduced by 68.2 ±10.1% in LD (*Unpaired* t-test: t=4.621, df=10, ***p<0.001) and by 102.1 ±14.6% in DD (*Unpaired* t-test: t=4.167, df=10, **p=0.001). (**K**) Schematic of the chemogenetic inhibition of DMH^Vglut2^ neurons by hGlyR and IVM. (**L**) Representative micrograph of hGlyR-mCherry expression in the DMH (in red). (**M**) IVM administration diminishes the Cort rise at ZT13 in LD (*Two-way ANOVA*; *Tukey’s* multiple comparisons test. ZT13 VEH vs IVM: **p=0.006) and almost entirely prevents it at CT13 in DD compared with vehicle (*Two-way ANOVA*; *Tukey’s* multiple comparisons test. CT13 VEH vs IVM: **p=0.001). (**N**) The Cort CI is reduced one day after the administration of IVM by 58.9 ±15.9% in LD (*Paired t-test*: t=3.334, df=8, *p=0.01) and by 92.3 ± 9.9% in DD (*Paired t-test*: t=2.903, df=8, *p=0.019). LD, Light:Dark photoperiod; DD, Constant dark; *= p<0.05, **= p<0.01, ***= p<0.001; Reference scale bar= 200μm; 3V, third ventricle.

To study the role of the DMH^Vglut2^ neurons in the circadian regulation of Cort, we first measured Cort levels in blood from 14 *Vglut2-ires-Cre* mice in a 12:12 light:dark (LD) cycle, by tail nicks at four time points (ZT 1, 7, 13, 19, taken randomly with at least 30 hrs between samplings), and on the same time points in constant darkness (DD) (CT 1, 7, 13, 19), starting on the third day at CT13, and then every 30 hours. Simultaneously, we recorded the baseline circadian rhythms of locomotor activity (LMA) and body temperature (Tb) for 12 days in LD, and then 12 days under DD. We then placed injections into the DMH of a viral vector expressing mCherry in cells devoid of Cre-recombinase (Cre) and diphtheria toxin A (DTA), which induces death of cells expressing Cre (*AAV10-hSyn-mCherry-DIO-DTA*). After four weeks to allow full expression of DTA, we measured Cort levels in LD and DD as described, and recorded LMA and Tb in the mice for 12 days in LD and 12 days in DD. From a total of 14 mice, a blinded observer identified 8 mice in which the injection covered at least 70% of the DMH bilaterally (Fig. 1A–B and **Ext. Fig. 1A**) and these mice were used for further analysis. DMH^Vglut2^ ablation reduced the Cort peak at the beginning of the active phase (ZT 13) from 39.4 ± 6 ng/ml in LD and 27.9 ± 3.5 ng/ml in DD before *AAV10-hSyn-mCherry-DIO-DTA* injections, to 23.4 ± 4.2 ng/ml in LD (p=0.007, Fig. 1C) and 15.5 ± 4.3 ng/ml in DD after DMH^Vglut2^ neuron ablation (p=0.013, Fig. 1D). The Circadian Index (CI) of Cort secretion was reduced by 46.6 ±14.2% in LD (p=0.043) and 62.0 ±17.3%in DD after the ablation (p=0.014, Fig. 1E). The ablation of DMH^Vglut2^ neurons mainly reduced the peak in LMA at the transitions between active and inactive periods both in LD and DD. Because these transition periods spanned the light and dark cycles, there was no change in the CI of LMA, although the amplitude of the LMA rhythm in DD was reduced in cosinor analysis (**Ext. Fig. 1B-I**). In contrast, ablation of DMH^Vglut2^ neurons reduced Tb during the light phase in LD and in both presumptive light and dark phases in DD. As a result, the CI and cosinor amplitude of the Tb rhythm was increased during LD (**Ext. Fig. 1J-Q**).

**Figure 2.**
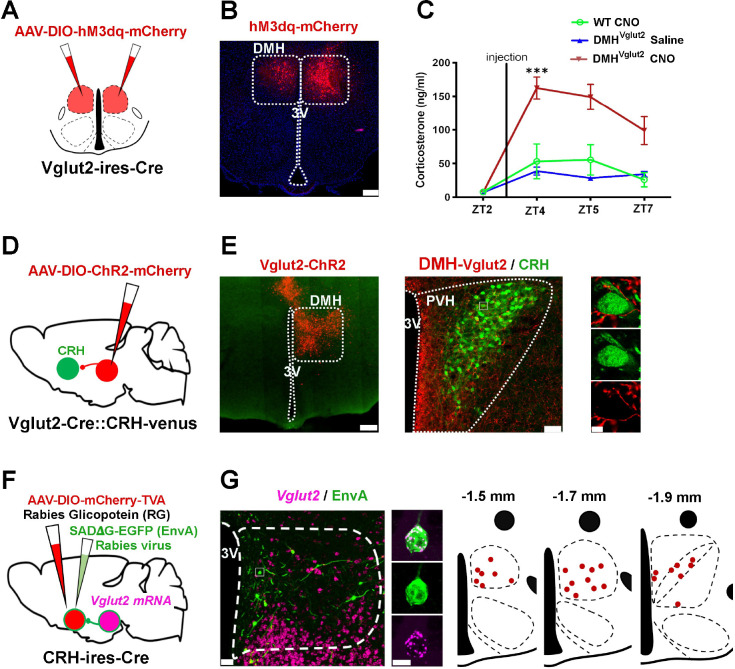
DMH^Vglut2^ neurons project to the CRH neurons of the PVH and their chemogenetic activation increases Cort. (**A**) Schematic of *AAV-DIO-hM3dq-mCherry* injection targeting the DMH^Vglut2^ neurons. (**B**) Representative image of a *AAV-DIO-hM3dq-mCherry* (in red) injection site in the DMH. (**C**) CNO-mediated chemogenetic-stimulation of DMH^Vglut2^ neurons boosts the Cort release (*Two-way ANOVA*; *Tukey’s* multiple comparisons test. CT4 WT CNO vs DMH^Vglut2^ CNO: ***p<0.001, CT4 DMH^Vglut2^ Saline vs DMH^Vglut2^ CNO: ***p<0.001). (**D**) Schematic of *AAV-DIO-ChR2-mCherry* injection targeting DMH^Vglut2^ neurons. (**E**) Representative photomicrograph of the DMH^Vglut2^ neurons expressing ChR2-mCherry (*left* panel, in red), their axon pattern in the PVH (*center* panel) and their appositions with CRH neurons (*right* panel, in green). (**F**) Schematic of the EnvA-rabies experiment to map the monosynaptic input from the DMH^Vglut2^ neurons to PVH^CRH^ neurons. (**G**) Representative images showing *Vglut2 mRNA* expression (in magenta) and rabies expression (in green) within the DMH and a higher magnification image showing a single doubly labeled DMH neurons (*left* panels). Mapping of the rabies-*Vglut2* co-labeling distribution thought the DMH (*right* panel). ***= p<0.001; Reference scale bar: in B and E (*left*)= 200 μm, E (*center*) and G (*left*)= 50μm, in E and G (*right* panels) = 10μm. 3V, third ventricle. Atlas levels are from Paxinos Atlas ^40^.

We then evaluated whether the effect of ablation of DMH^Vgut2^ neurons on Cort rhythms was due to the loss of glutamate transmission, as opposed to other possible transmitters colocalized and expressed in DMH^Vgut2^ neurons. We therefore injected the DMH of *Vglut2*^*loxP/loxP*^ (*Vglut2-flox*) mice with either *AAV8-SYN-EGFP-iCre* (*AAV8-EGFP-iCre*) or a control virus (*AAV8-DIO-GFP*) and recorded LMA, Tb and Cort levels in both LD and DD. Seven of the 11 mice injected with *AAV8-EGFP-iCre* had injections covering at least 70% of the DMH bilaterally were compared with 5 mice with *AAV8-DIO-GFP* injections in the DMH of *Vglut2-flox* mice as controls (Fig. 1F–G, **Ext. Fig. 2A**). In these animals, the Cort levels at ZT13 were reduced in LD from 28.9 ± 1.9 ng/ml to 13.2 ± 2.6 ng/ml (p<0.001, Fig. 1H) and in DD from 40.4 ± 7.5 ng/ml to 11.7 ± 3.5 ng/ml in DMH^Vglut2^-GFP and DMH^Vglut2^-EGFP-iCre mice, respectively (p<0.001, Fig. 1I). The CI of Cort was reduced by 68.2 ±10.1% in LD (p<0.001) and by 102.1 ±14.6% in DD (p=0.001, Fig. 1J). Both the LMA and Tb were reduced during the dark and subjective dark phase, and at the transition from the dark to the light phase during LD and at the presumptive transition in DD (**Ext. Fig. 2**). However, the CI of neither LMA nor Tb was affected by the loss of glutamatergic signaling in the DMH.

**Figure 3.**
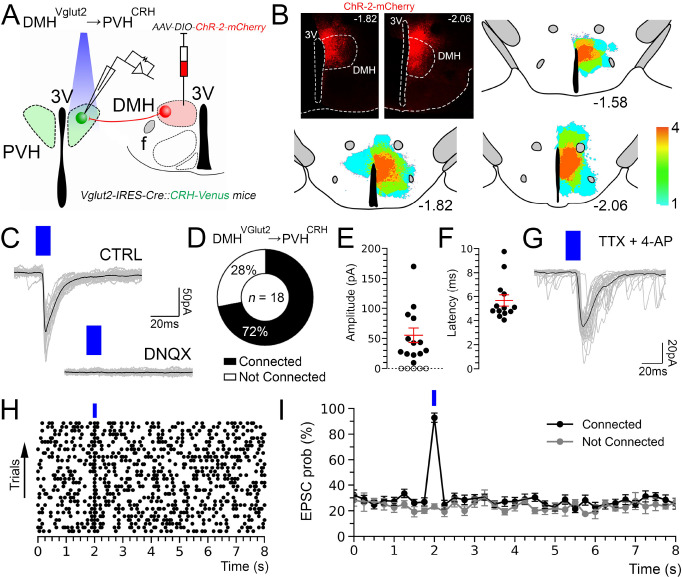
In vitro optogenetic stimulation of the glutamatergic input from the DMH directly excites PVH^CRH^ neurons. (**A**) A schematic of the experiment testing the proposed DMH^Vglut2^ → PVH^CRH^ connectivity; *Vglut2-ires-Cre::CRH-Venus mice* were injected with *AAV-DIO-ChR2-mCherry* in the DMH and recordings were conducted in brain slices in Venus-labeled PVH^CRH^ neurons while photostimulating the DMH^VGlut2^ input. (**B**) An example of ChR2-mCherry expression after an injection the DMH (*top left*) and density plots of the *AAV-DIO-ChR2-mCherry* injection sites (*n*=4 mice; *right* and *bottom*). (**C**) AMPA-mediated opto-stimulated excitatory post-synaptic currents (oEPSCs) recorded in PVH^CRH^ neurons (*upper trace)* and blockade by DNQX, 200μM (*lower trace*; *n*=4). (**D**) Percentages of PVH^CRH^ neurons responding (*Connected*) and not responding (*Not Connected*) to photostimulation of the DMH^Vglut2^ input (*n*=18, total PVH^CRH^ recorded neurons). (**E**) Amplitude and (**F**) latency of oEPSCs in PVH^CRH^ neurons in response to photostimulation of the DMH^Vglut2^ input (*n*=13; mean and ± SEM). (**G**) oEPSCs in PVH^CRH^ neurons recorded in TTX 1μM + 4-AP 500μM (*n*=6) indicating monosynaptic connectivity. (**H**) Raster plot of EPSCs in a representative PVH^CRH^ neuron with photostimulation of the DMH^Vglut2^ → PVH^CRH^ input (bin duration: 50ms). (**I**) EPSC probability in response to photostimulation of the DMH^Vglut2^ → PVH^CRH^ input (black, *n*=13; grey, *n*=5). Reference scale bar: in B = 250 μm. f, fornix, 3V, third ventricle. Atlas levels are from Paxinos Atlas ^40^.

As the DMH^Vglut2^ ablation and the *Vglut2* mRNA deletion are chronic lesions that might be affected by compensatory mechanisms, we decided to test the effect of acute inhibition of the DMH^Vglut2^ neurons on Cort, LMA and Tb rhythms. We injected the DMH in 9 *Vglut2-ires-Cre* mice with a viral vector containing a modified human alpha-1 glycine receptor (*AAV10-DIO-hGlyR-mCherry*) which is insensitive to glycine but has 100-fold increased sensitivity to the antiparasitic drug ivermectin (IVM) compared to the unmutated glycine receptor ^12^. Because the half-life of IVM is about 72 hrs, the injection of IVM induces long-lasting neuronal inhibition (4–5 d). To allow the drug to achieve a stable concentration in the CNS, we examined Cort levels between 24 and 48 hrs after drug administration ^13^ compared with injection of vehicle. We administered vehicle (VEH) or IVM (5mg/Kg, ip) one hour after the light onset or subjective light onset and sampled the mice the next day at ZT1 and ZT13 to evaluate the Cort levels, with at least 7 days of washout between injections. In 5 mice in which expression of mCherry was seen in at least 70% of the DMH bilaterally (Fig. 1K–L, **Ext. Fig. 3A**), the Cort levels at ZT13 under LD were reduced from 31.7 ± 2.3 ng/ml after VEH to 15.6 ± 4.8 ng/ml after IVM (p=0.006), and under DD from 28.1 ± 6.9 ng/ml after VEH to 9.3 ± 1.8 ng/ml after IVM (p=0.001, see Fig. 1M). The inhibition of the DMH^Vglut2^ neurons with IVM reduced the Cort CI by 58.9 ±15.9% in LD (p=0.01) and by 92.3 ± 9.9% in DD (p=0.019, see Fig. 1N). The average CI of LMA after IVM administration was reduced by 103.3 ± 21.1% in LD (i.e., the day-night difference was reversed due to higher levels of LMA during the light period) (p=0.011, **Ext. Fig. 3B-D**), and was reduced in DD by 81.3 ± 9.1% when compared with vehicle (p=0.016, **Ext. Fig. 3E-G**), however, the total LMA was not significantly different at 24–48h after IVM both in DD and LD (**Ext. Fig. 3B-G**). IVM also induced a decrease in Tb during the dark period, with a reduction of 0.5 ± 0.2°C observed in LD compared with vehicle administration (p=0.024, **Ext. Fig. 3H-J**). In DD conditions, the decrease in Tb during presumptive night was 0.4 ± 0.1°C (p=0.014, **Ext. Fig. 3K-M**). The Tb CI was reduced in LD by 38.4 ± 6.2% after administration of IVM (p=0.025, **Ext. Fig. 3J**).

These data clearly show that glutamatergic transmission by the DMH^Vglut2^ neurons plays an important role in the increase of Cort levels at the beginning of the active phase. The ablation or inhibition of DMH^Vglut2^ neurons reduces peak of Cort to the early morning levels. The DMH^Vglut2^ neurons also increase LMA in a crepuscular pattern (at the transitions between the light and dark periods) and their loss causes a small (approximately 0.3–0.5 °C) decrease in Tb, but has little if any effect on the circadian rhythm of Tb.

### Glutamatergic DMH neurons send monosynaptic projections to the PVH^CRH^ neurons and boost Cort levels

As the ablation of the DMH^Vglut2^ neurons reduced the peak of Cort levels, we hypothesized that activation of these neurons may elevate the Cort levels at the beginning of the active phase. First, we injected the DMH of 15 *Vglut2-ires-Cre* mice with *AAV10-EF1α-DIO-hM3Dq-mCherry* (Fig. 2A–B). Five to six weeks later, the mice were injected i.p. with clozapine-N-oxide (CNO) 0.3 mg/kg at ZT3. The acute activation of DMH^Vglut2^ neurons caused an increase in Cort levels from 7.4 ± 2.1 ng/ml just before the animals received the dose of CNO, to 162.5 ± 16.4 ng/ml 1 hr after the administration of CNO (p<0.001, see Fig. 2C). This increase is around three times higher than the Cort levels detected in the same mice that received saline or in WT mice injected with CNO. These results confirm that activation of the DMH^Vglut2^ neurons boosts Cort release.

**Figure 4.**
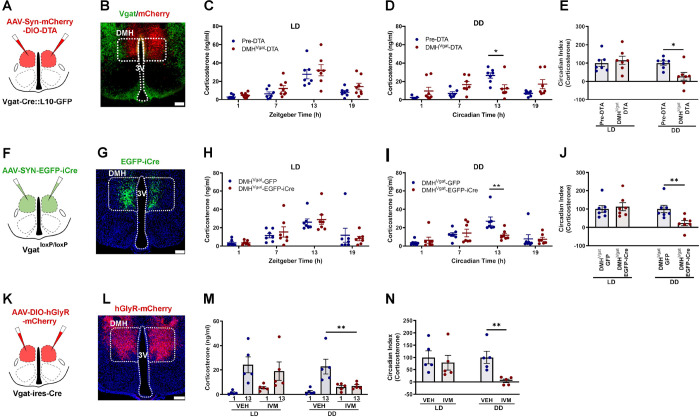
Ablation of the DMH^Vgat^ neurons reduces the endogenous circadian peak of Cort release only in constant dark. (**A**) Schematic representation of the DMH^Vgat^ neuron ablation. (**B**) Representative micrograph showing GABAergic neurons (in green) and *AAV-DIO-DTA-mCherry* (in red) in the DMH of a *Vgat-ires-Cre::L10-GFP* mice. There are no remaining GABAergic neurons in the region of mCherry expression. (**C**) DMH^Vgat^ neuron ablation does not alter the daily Cort release in LD, (**D**) but eliminates it almost entirely under DD photoperiod (*Two-way ANOVA*; *Tukey’s* multiple comparisons test. CT13 Pre-DTA vs DMH^Vgat^-DTA: *p=0.014). (**E**) The Cort CI was not significantly changed by DMH^Vgat^ ablation in LD, but was reduced to 72.4 ±21.3% in DD. (*Paired t-test*: t=3.028, df=12, *p=0.01). (**F**) Schematic representation of *Vgat* gene deletion in the DMH. (**G**) Representative photomicrograph showing the EGFP expression in *Vgat*-deleted neurons (green). (**H**) *Vgat* deletion from DMH neurons does not change the circadian regulation of Cort release in LD, (**I**) but diminishes the daily peak of Cort at CT13 in DD (*Two-way ANOVA*; *Tukey’s* multiple comparisons test. CT13 DMH^Vgat^-GFP vs DMH^Vgat^-EGFP-iCre: **p=0.006). (**J**) The CI of Cort secretion is not affected by *Vgat* gene deletion in the DMH in LD but is reduced by 76.6 ±13.6% in DD in DMH^Vgat^ deleted mice (*Unpaired t-test*: t=3.160, df=12, **p=0.008). (**K**) Schematic representation of the *AAV-DIO-hGlyR-mCherry* injection in the DMH of *Vgat-ires-Cre* mice. (**L**) Representative micrograph of *hGlyR-mCherry* expression (in red) in the Vgat neurons of the DMH. (**M**) IVM chemo-inhibition of the DMH^Vgat^ neurons by hGlyR does not alter the Cort levels in LD, but prevents the peak at CT13 in DD (*Two-way ANOVA*; *Tukey’s* multiple comparisons test. CT13 VEH vs IVM: **p=0.006). (**N**) The Cort CI is reduced by 95.7 ±6.9% in DD (*Paired t-test*: t=3.641, df=8, **p=0.006). LD, Light:Dark photoperiod; DD, Constant dark; *= p<0.05, **= p<0.01, Reference scale bar= 200μm; 3V, third ventricle.

To determine whether the DMH^Vglut2^ neurons make direct synaptic contacts onto the PVH^CRH^ neurons to activate them and so to increase Cort levels, we conducted conditional anterograde tracing and conditional monosynaptic retrograde rabies tracing studies. We first evaluated the projection pattern of DMH^Vglut2^ neurons using *Vglut2-ires-Cre* mice crossed with reporter mice that express the fluorescent protein Venus (green) in the CRH expressing neurons (*Vglut2-ires-Cre::CRH-Venus*). Following injection of *AAV8-DIO-ChR2-mCherry* into the DMH, we found a dense projection from DMH^Vglut2^ neurons to the PVH that formed appositions with the PVH^CRH^ neurons (Fig. 2D–E). To confirm direct connection between the DMH^Vglut2^ and PVH^CRH^ neurons, we injected the PVH of 5 *CRH-ires-Cre* mice with an AAV expressing TVA avian receptor (*AAV8-Ef1a-DIO-TVA-mCherry*) and an AAV expressing rabies glycoprotein necessary for rabies viral transfection (*AAV8-CAG-DIO-rabiesG*) specifically in the PVH^CRH^ neurons. Twenty-one days later, to label and map the direct monosynaptic inputs to the PVH^CRH^ neurons, we injected a glycoprotein (G)-deleted rabies virus (RVdG) that expresses EGFP and is enveloped with the avian ASLV type A protein (EnvA), which utilizes the TVA receptor for cell entry (*EnvA-ΔG-rabies-GFP*). This results in PVH^CRH^ neurons infected by both AAV and rabies viruses (starter cells) displaying both green and red fluorescent signals, while neurons expressing only EGFP-Rabies (green) were retrogradely labeled by the viral particles produced in the starter cells (**Ext. Fig. 4A-B**). Transneuronally infected neurons were seen in areas previously reported to project to the PVH such as the bed nucleus of the stria terminalis, preoptic area, arcuate nucleus, and DMH, and we also observed a large number of retrogradely labeled neurons within the PVH itself and its immediate surrounding areas (**Ext. Fig. 4C-D**) (Douglass et al., 2023). We found few, if any, EGFP-Rabies infected neurons in the SCN, which supports the hypothesis that circadian control of Cort secretion by the SCN requires intermediate relays such as the DMH.

We also combined rabies tracing from PVH^CRH^ neurons with *in situ* hybridization for *Vglut2* mRNA. We found that 57.3 ± 15.9% (*n*=2) of retrogradely-EGFP labeled neurons expressed *Vglut2*, confirming that DMH^Vglut2^ neurons are likely to directly innervate PVH^CRH^ cells. In particular, we observe that the largest number of retrogradely labeled neurons expressing *Vglut2* were located in the anterior portion of the DMH (Fig. 2F–G). By contrast, retrogradely labeled neurons that expressed *Vgat* mRNA were found throughout the DMH, but with a more caudal predominance (**Ext. Fig. 4E-F**), indicating that there is also a direct GABAergic input predominantly from the caudal DMH to the PVH^CRH^ neurons (see below).

### Stimulation of the DMH^Vglut2^ neurons directly excites PVH^CRH^ neurons

To functionally test the input from the DMH^Vglut2^ neurons to the PVH^CRH^ neurons (DMH^Vglut2^ → PVH^CRH^), we conducted *in vitro* channelrhodopsin-2(ChR2)-assisted circuit mapping (CRACM) recordings. We injected the DMH with an *AAV8-DIO-ChR2-mCherry* in a new cohort of 4 *Vglut2-ires-Cre::CRH-Venus* mice and then three to four weeks later we recorded from labeled PVH^CRH^ neurons in brain slices while photo-stimulating axons and terminals of DMH^Vglut2^ neurons within the PVH (Fig. 3A–B). Optogenetic stimulation of the DMH^Vglut2^ input evoked excitatory synaptic responses in 72% of the PVH^CRH^ neurons recorded (*n*=18). This effect was mediated by the release of glutamate and AMPA receptor signaling as the opto-evoked excitatory postsynaptic currents (oEPSCs) were blocked by the AMPA receptor antagonist DNQX (Fig. 3C–F). Furthermore, the oEPSCs persisted in the presence of TTX (TTX 1μM) and 4-aminopyridine (4-AP), 200–500 μM) indicating monosynaptic connectivity (*n*=6 out of 8; Fig. 3G). These experiments confirm the direct synaptic connectivity from DMH^Vglut2^ neurons to PVH^CRH^ neurons and are consistent with our observations that: 1) axons from DMH^Vglut2^ neurons extensively branched among the Venus-labelled PVH^CRH^ neurons (Fig. 2D), and 2) DMH^Vglut2^ neurons drive the elevation of Cort levels during the early active period by directly stimulating the release of CRH from PVH^CRH^ neurons via AMPA receptors (Fig. 1).

### Ablation, inhibition, or disruption of GABAergic signaling in DMH^Vgat^ neurons reduces the daily peak in Cort secretion only under constant darkness

**Figure 5.**
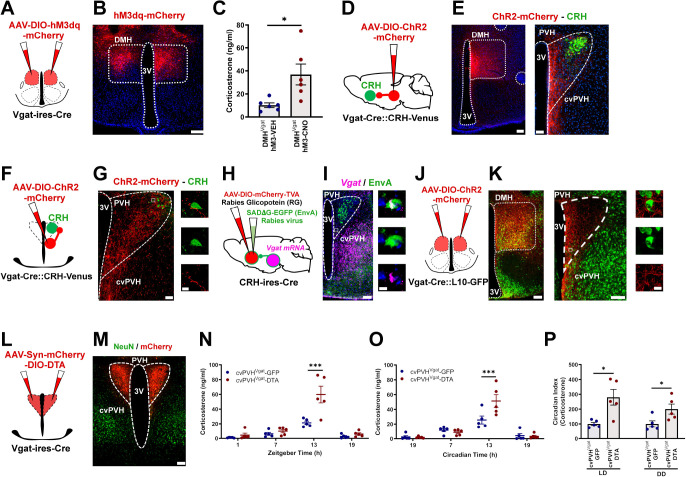
Chemo-activation of DMH^Vgat^ neurons elevates Cort levels through cvPVH GABAergic neurons. (**A**) Schematic of *AAV-DIO-hM3dq-mCherry* injections in the DMH in Vgat-ires-Cre mice. (**B**) Representative micrograph of hM3dq-mCherry expression (red) in the DMH. (**C**) Chemogenetic activation of the DMH^Vgat^ neurons increased Cort levels from 10.2 ±2 ng/ml after VEH to 36.8 ±9 ng/ml after CNO administration (*Paired t-test:* t=2.875, df=10, *p=0.016). (**D**) Schematic of a*AAV-DIO-ChR2-mCherry* injection in the DMH. (**E**) Representative micrograph of rostral DMH^Vgat^ neurons expressing ChR2-mCherry (in red, *left* panel), and their axon pattern in the PVH. Note that DMH^Vgat^ neuron projections mostly avoid the PVH^CRH^ neurons (in green) but are dense in the ventral-medial and caudal portion of the PVH. (**F**) Schematic of an *AAV-DIO-ChR2-mCherry* injection in the cvPVH^.^ (**G**) Representative image of the cvPVH^Vgat^ cell bodies and their axons expressing ChR2-mCherry (in red) in the ventral part of the PVH^CRH^ neuron field (in green), and their appositions to the PVH^CRH^ neurons. (**H**) Schematic of the EnvA-Rabies experiment to map monosynaptic inputs from the cvPVH^Vgat^ neurons to PVH^CRH^ neurons. (**I**) Representative photomicrograph showing the *Vgat mRNA* expression (in magenta) and the Rabies expression (in green) and a doubly labeled neuron within the cvPVH. (**J**) Schematic of the *AAV-DIO-ChR2-mCherry* injections in the DMH. (**K**) Micrograph showing the expression of ChR2-mCherry (in red) in the DMH^Vgat^ neurons (in green, *left* panel), and their projections and appositions to the cvPVH^Vgat^ neurons (*right* and *center* panels). (**L**) Schematic of the cvPVH^Vgat^ neuron ablation. (**M**) Representative image of the injection site showing mCherry expression (in red) in non-Vgat neurons in the PVH. (**N**) The cvPVH^Vgat^ ablation boosted the Cort increase at ZT13 in LD (*Two-way ANOVA*; *Tukey’s* multiple comparisons test. ZT13 cvPVH^Vgat^-GFP vs cvPVH^Vgat^-DTA: ***p<0.001), and (**O**) at CT13 in DD (*Two-way ANOVA*; *Tukey’s* multiple comparisons test. CT13 cvPVH^Vgat^-GFP vs cvPVH^Vgat^-DTA: ***p<0.001). (**P**) The Cort CI increased after the cvPVH^Vgat^ neuron ablation by 180.2 ±52.9% in LD (*Unpaired t-test:* t=3.336, df=8, *p=0.01) and 100.4 ±33.9% in DD (*Unpaired t-test:* t=2.512, df=8, *p=0.036). LD, Light:Dark photoperiod; DD, Constant dark; *= p<0.05, ***= p<0.001, Reference scale bar: in B, E, K and M= 200 μm, E (right), G, I and K (center) = 50μm, in G, I and K (right panels) = 10μm; 3V, third ventricle.

Although inputs from DMH^Vglut2^ neurons to PVH^CRH^ cells are necessary to drive the daily Cort increase, their ablation does not completely eliminate the circadian rhythm of Cort secretion as seen with non-specific DMH ablation. It has been estimated that half of the synapses to the PVH^CRH^ neurons are GABAergic ^14^, and our rabies virus experiment showed that about half of the DMH cells innervating the PVH^CRH^ neurons are GABAergic. Thus, we decided to evaluate whether the DMH^Vgat^ neurons could play a complementary role in the circadian release of Cort. We hypothesized that the DMH^Vgat^ neurons could contribute to the circadian rhythms of Cort secretion either by inhibiting PVH^CRH^ neurons during the inactive cycle to suppress Cort secretion, or by dis-inhibiting PVH^CRH^ neurons (i.e., inhibiting inhibitory inputs to them) during the active cycle. Thus, we used *Vgat-ires-Cre* mice crossed with a Cre-dependent GFP reporter mouse line *R26-loxSTOPlox-L10-GFP* to generate *Vgat-ires-Cre::R26-loxSTOPlox-L10-GFP* mice (*Vgat-ires-Cre::L10*), in which Vgat neurons expressing Cre-recombinase show green fluorescence. We collect blood samples at 4 temporal points in LD (ZT 1, 7, 13, 19), and then in the same temporal points in DD (CT 1, 7, 13, 19), as we did with the *Vglut2-ires-Cre* mice. Also, we recorded LMA and Tb for 12 days in LD, and then12 days in DD. Then, we injected the DMH of *Vgat-ires-Cre::L10* mice with an *AAV10-hSyn-mCherry-DIO-DTA* to ablate the DMH^Vgat^ neurons. Four weeks after the injections, we recorded and took blood samples from the mice in LD and DD conditions. From a total of 13 *Vgat-ires-Cre::L10* mice injected, we analyzed data from 8 mice in which a blinded investigator found that the injection sites covered at least 70% of the DMH bilaterally (Fig. 4A–B, **Ext. Fig. 5A**). DMH^Vgat^ ablation did not alter the daily rhythm of Cort release in LD but reduced the CT13 peak under DD from 26.5 ± 2.9 ng/ml in Ctrl to 14.1 ±4.4 ng/ml (p=0.014, Fig. 4C–D). Similarly, there was no statistically significant change in the CI of Cort in LD, but it was reduced by 72.4 ±21.3% in DD after ablation of DMH^Vgat^ neurons (p=0.01, Fig. 4E). By contrast, LMA was dramatically reduced during the dark phase after DMH^Vgat^ ablation in LD, the total count over the dark period decreasing from 873.5 ± 45 counts to 429.5 ± 52.5 counts (p<0.001); LMA was reduced even further in DD during the presumptive dark phase from 771.2 ± 40.6 counts to 401.7 ± 44.9 counts (p<0.001, **Ext. Fig. 5F**), resulting in a reduction in the Circadian Index of LMA by 38.23 ±9.2% in LD (p=0.005) and by 50.61 ±12.9% in DD (p=0.003, **Ext. Fig. 5G-H**), and a significant reduction in the amplitude of the peak at 24 hrs in the periodogram and in the cosinor analysis of both LD and DD (LD: p<0.001; DD: p<0.001, **Ext. Fig. 5C and E**). Thus, most of the reduction in LMA after DMH lesions appears to be due to loss of DMH^Vgat^ neurons. In the mice with ablation of DMH^Vgat^ neurons, Tb was about 0.3°C lower throughout the day in LD and during the subjective dark period in DD, but there was no statistically significant change in the CI or cosinor analisys (**Ext. Fig. 5J-Q**).

**Figure 6.**
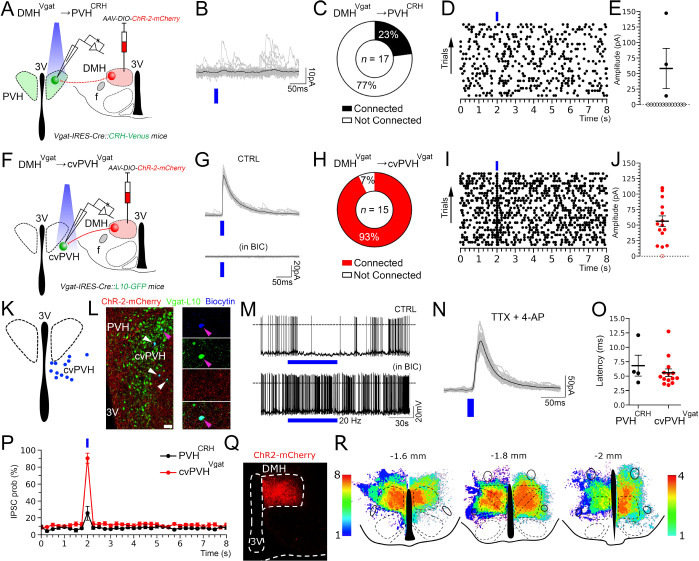
In vitro optogenetic stimulation of the GABAergic input from the rostral DMH spares PVH^CRH^ neurons and directly inhibits GABAergic neurons in the cvPVH. (**A**) A schematic diagram of experiments testing rDMH^Vgat^ → PVH^CRH^ connectivity; *Vgat-ires-Cre::CRH-Venus* mice were injected with *AAV-DIO-ChR2-mCherry* in the rDMH and recordings were conducted in brain slices from Venus-labeled PVH^CRH^ neurons while photostimulating the DMH^Vgat^ input. (**B**) Stimulation of the rDMH^Vgat^ input produced no synaptic responses time-locked to the light pulses in most of the PVH^CRH^ neurons. (**C**) Percentages of PVH^CRH^ neurons responding (Connected) and not responding (Not Connected) to photostimulation of the rDMH^Vgat^ input (*n*=17, total PVH^CRH^ recorded neurons). (**D**) Raster plot of IPSCs in a representative PVH^CRH^ neuron with photostimulation of the rDMH^Vgat^ → PVH^CRH^ input (bin duration: 50ms) showing lack of input. (**E**) oIPSC amplitude following photostimulation of the rDMH^Vgat^ → PVH^CRH^ input (filled markers, cells responding to photostimulation, *n*=4; open markers, cells not responding to photostimulation, *n*=13; mean and ± SEM of responding neurons). (**F**) To explore rDMH^Vgat^ → cvPVH^Vgat^ connectivity, *Vgat-ires-Cre::L10-GFP* mice (*n*=5) were injected with *AAV-DIO-ChR2-mCherry* in the rDMH, and recordings were conducted in GFP-labeled cvPVH^Vgat^ neurons while photostimulating the rDMH^Vgat^ input. (**G**) Photostimulation of the DMH^Vgat^ input evoked oIPSCs in most of the cvPVH^Vgat^ neurons, and these were GABA_A_-mediated (blocked by bicuculline, BIC 20μM; *n*=4). (**H**) Percentages of cvPVH^Vgat^ neurons responding (Connected) and not responding (Not Connected) to photostimulation of the rDMH^Vgat^ input (*n*=15, total cvPVH^Vgat^ recorded neurons). (**I**) Raster plot of IPSCs in a representative cvPVH^Vgat^ neuron with photostimulation of the rDMH^Vgat^ → cvPVH^Vgat^ input (bin duration: 50ms). (**J**) oIPSC amplitude following photostimulation of the rDMH^Vgat^ → cvPVH^Vgat^ input (filled markers, cells responding to photostimulation, *n*=14; open markers, cells not responding to photostimulation, *n*=1; mean and ± SEM of responding neurons). (**K**) The distribution of recorded cvPVH^Vgat^ neurons receiving input from DMH^Vgat^ neurons is shown as a schematic map of 12 recorded neurons. (**L**) A photomicrograph shows four recorded GABAergic cvPVH neurons that responded to photostimulation of DMH^Vgat^ input (filled with biocytin from the recording pipette, indicated by arrowheads). DMH^Vgat^ fibers expressing ChR2-mCherry (in red) surrounded the cvPVH^VGAT^ neurons expressing GFP (in green). The neuron indicated by the magenta arrowhead is shown at higher magnification at the right, showing labeling, from top to bottom, for biocytin, GFP, mCherry, and merged. (**M**) Photostimulation trains (20Hz, train frequency; 60s, train duration and 10ms, pulse duration) inhibited the activity of the cvPVH^Vgat^ neurons (*n*=6; *top*) and this effect was blocked by bicuculline (20μM; *n*=2; *bottom*). (**N**) oIPSCs in cvPVH^Vgat^ neurons recorded in the presence of TTX 1μM + 4-AP 200μM (*n*=6) indicating monosynaptic connectivity. (**O**) oIPSC latency recorded in PVH^CRH^ (black; *n*=4) and cvPVH^Vgat^ neurons (red; *n*=14) following photostimulation of the DMH^Vgat^ input. (**P**) oIPSC probability in PVH^CRH^ (black; *n*=4) and cvPVH^Vgat^ (red; *n*=14) neurons following photostimulation of the DMH^Vgat^ input. (**Q-R**) Photomicrograph of a representative rDMH injection with *AAV-DIO-ChR2-mCherry* and heat maps of injections in the rDMH (*n*= 8, including experiments in A-E and F-N; illustrated on the left side of each section) following which only 23% of PVH^CRH^ neurons showed oIPSCs compared to injections in the caudal DMH (n=4; illustrated on the right side of each section) after which most of the PVH^CRH^ neurons showed oIPSCs. Reference scale bar: in L (*left*) = 50 μm and (*right*) = 20 μm; in Q = 250 μm. Atlas levels are from Paxinos Atlas ^40^.

We interpreted these results as indicating that it was unlikely that DMH^Vgat^ neurons played a role in suppressing Cort secretion during the inactive (light) phase, but that they potentially accounted for the other half (not caused by the DMH^Vglut2^ neurons) of the surge in Cort levels during the active (dark) phase. We next evaluated whether this effect was due to GABA release from these neurons (vs. some other neurotransmitter in the same neurons). Thus, we placed *AAV8-SYN-EGFP-iCre* or *AAV8-DIO-GFP* injections in the DMH on *Vgat*^*loxP/loxP* (^*Vgat-flox*) mice to delete expression of functional Vgat protein and so to abolish GABA transmission from the GABAergic neurons of the DMH. From 12 mice injected with AAV-EGFP-iCre in the DMH, 7 with bilateral injections covering at least 70% of the DMH were included in the analysis (Fig. 4F–G, **Ext. Fig. 6A**). *Vgat* deletion from DMH neurons did not change the circadian regulation of Cort release in LD, but again reduced the CI of Cort release in DD by 76.6 ±13.6% (p=0.008, Fig. 4J), by reducing the CT13 peak of Cort levels in DD from 27.0 ± 4.5 ng/ml in controls to 11.7 ± 2.1 ng/ml in *Vgat-*deleted mice (p=0.006, Fig. 4H). Thus, most if not all of the effect of the DMH^Vgat^ neurons on the circadian release of Cort is mediated by GABA. Interestingly, although the total levels of LMA during the dark period were decreased by Vgat deletion in the DMH (from 979.2 ± 66.8 counts in the control group to 681.5 ± 55.6 counts in the deletion group during LD; p<0.001, **Ext. Fig. 6F**), the reduction was not nearly as profound as seen after the ablation of the DMH^Vgat^ neurons, suggesting that other transmitters released from those neurons could play a role in regulating LMA. Results in DD were similar (from 979.2 ± 66.8 total counts in DMH^Vgat^-GFP group to 681.5 ± 55.6 counts in the DMH^Vgat^-EGFP-iCre during DD; p<0.001, **Ext. Fig. 6F**), so that the CI of LMA was reduced by only 25.7 ± 6% in LD (p=0.001) and 33.3 ± 12.6% in DD (p=0.021, **Ext. Fig. 6G**), similar to the effect observed in the amplitude in the cosinor analysis (**Ext. Fig. 6G-H**). Tb of DMH^Vgat^ –deleted mice was also reduced during the dark phase by 0.2 °C in LD (mostly between ZT14–18; p=0.001, **Ext. Fig. 6J-N**), and in the subjective dark by 0.3°C in DD (p<0.001, **Ext. Fig. 6L-N**). As a result, the Circadian Index of Tb was reduced in the DMH^Vgat^–deleted mice by 20.8 ± 8.8% in LD (p=0.037) and by 26.6 ± 8.9% in DD (p=0.012, **Ext. Fig. 6O**), similar to the reduction in the amplitude in the cosinor analysis (**Ext. Fig. 6O-P**).

**Figure 7.**
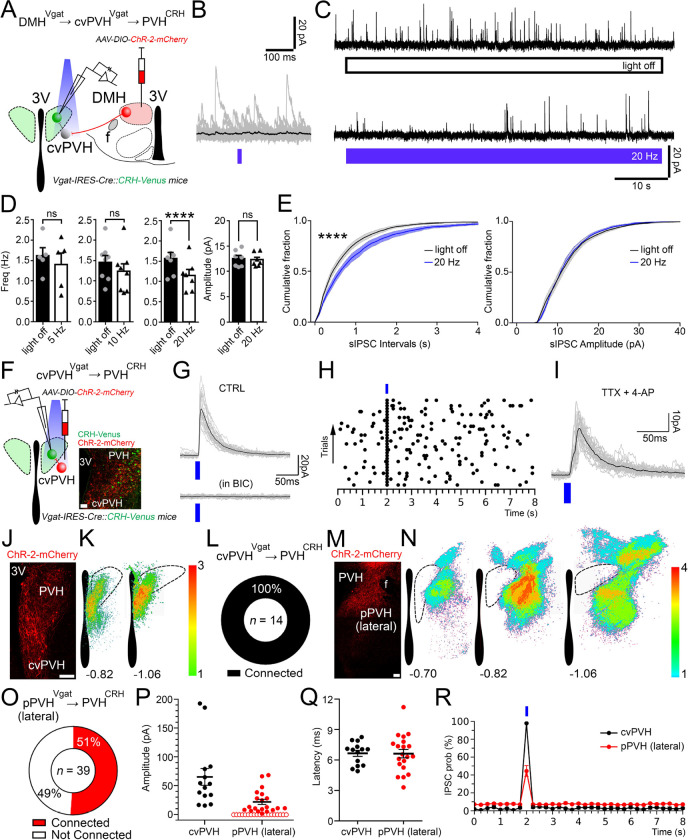
Rostral DMH^Vgat^ neurons disinhibit the PVH^CRH^ neurons via Vgat neurons in the cvPVH. (**A**) To test the rDMH^Vgat^ → cvPVH^Vgat^ → PVH^CRH^ circuit, we injected *AAV-DIO-ChR2-mCherry* into the rDMH of *Vgat-ires-Cre::CRH-Venus* mice (*n*=3) and recorded from PVH^CRH^ neurons while photostimulating rDMH^Vgat^ synaptic terminals. We tested the effects of single light pulses (10ms) and trains of optostimulation (10ms individual pulse duration; 5, 10, and 20Hz stimulation frequency; 60s train duration) on spontaneous IPSC (sIPSC) frequency in PVH^CRH^ neurons. (**B**) Single light pulses did not produce oIPSCs in most PVH^CRH^ neurons, but (**C**) trains of stimulation reduced the sIPSCs (*C; top* trace: light off; *bottom* trace: *light* on). (**D**) Change in sIPSC frequency following trains of photostimulation at 5, 10 and 20 Hz compared to sham stimulation (light off). Photostimulation at 5 and 10 Hz showed a trend toward reduction in sIPSC frequency that did not reach statistical significance (at 5 Hz: *n*=5, *paired t-test, p*=0.2772; at 10 Hz: *n*=8, *paired t-test, p*=0.0545) whereas photostimulation at 20 Hz significantly reduced sIPSCs frequency (*n*=7, paired t-test, ****p<0.0001), without affecting their amplitude (*n*=7; *paired t-test, p*= 0.7593). (**E**) Mean cumulative distribution plots of the sIPSC inter-event intervals show that intervals between sIPSCs were longer (*left*; 0.1s bins; Kolmogorov-Smirnov test, ****p<0.0001; 20Hz vs light off) but that there was no change in sIPSC amplitude (*right*; 1pA bins; *Kolmogorov-Smirnov test, p=*0.1414; 20Hz vs light off) as compiled from 7 PVH^CRH^ neurons (blue: 20Hz; black: light off; shaded areas: ± SEM). (**F**) To test the cvPVH^Vgat^ → PVH^CRH^ input, we injected *AAV-DIO-ChR2-mCherry* into the cvPVH of *Vgat-ires-Cre::CRH-Venus* mice (*n*=3) and recorded from PVH^CRH^ neurons while photostimulating cvPVH^Vgat^ neurons and terminals. (**G**) Stimulation of the cvPVH^Vgat^ input evoked GABA_A_-mediated oIPSCs in PVH^CRH^ neurons (Bicuculline, 20μM; *n*=4). (**H**) Raster plot of IPSCs in a representative PVH^CRH^ neuron showed tight correlation with photostimulation of the cvPVH^Vgat^ → PVH^CRH^ input (bin duration: 50ms). (**I**) TTX -resistant oIPSCs in cvPVH^CRH^ neurons (*n*=6; TTX 1μM + 4-AP 250μM)) indicating monosynaptic connectivity of the cvPVH^Vgat^ → PVH^CRH^ input. (**J-K**) A representative photomicrograph and heat maps of injections of AAV-ChR2-mCherry in the cvPVH (*n*=3) and (**L**) percentages of PVH^CRH^ neurons responding to photostimulation of the cvPVH^Vgat^ input (*L*; Connected; *n*=14 total PVH^CRH^ recorded neurons). (**M-N**) Representative injection site and heat maps of injections of AAV-ChR2-mCherry along the lateral margin of the PVH (pPVH) (*n*=4) and (**O**) percentages of PVH^CRH^ neurons responding (Connected) and not responding (Not Connected) to photostimulation of the GABAergic input from the lateral pPVH (*n*=39; *O*). (**P**) Amplitude and (**Q**) latency of oIPSCs in PVH^CRH^ neurons evoked by photostimulation of the cvPVH (black; *n*=14) and lateral PVH (red; *n*=39 mean and ± SEM). (**R**) oIPSC probability in PVH^CRH^ neurons following photostimulation of the input from the cvPVH^Vgat^ (black; *n*=14) and lateral pPVH^Vgat^ neurons (red; *n*=39). Reference scale bar: in G = 50 μm; K and N = 100 μm. f, fornix, 3V, third ventricle. Atlas levels are from Paxinos Atlas ^40^.****, *p* < 0.0001.

To determine whether these chronic conditional lesions or impairments of GABA transmission might be affected by compensatory mechanisms, we examined the effect of acute inhibition of the DMH^Vgat^ neurons on the circadian release of Cort. We placed injections of *AAV-DIO-hGlyR-mCherry* in the DMH of *Vgat-ires-Cre* mice. From 7 mice injected, 5 mice with bilateral injection covering at least 70% of the DMH were included for analysis (Fig. 4K–L, **Ext. Fig. 7A**). After 4 weeks allowing the full expression of hGlyR, mice were injected with either vehicle or IVM (5mg/Kg, ip) at ZT1 in LD, or CT1 in DD, and Cort levels were sampled 24h and 36h later. The inhibition of the DMH^Vgat^ neurons did not change the Cort levels under LD, but reduced the level at CT13 from 22.8 ± 6.1 ng/ml after vehicle to 6.9 ± 1.1 ng/ml after IVM administration (p=0.006, Fig. 4M) and reduced the CI by 95.7 ±6.9% in DD in DD (p=0.006, Fig. 4N). Similar to the effects of their ablation, the inhibition of DMH^Vgat^ neurons reduced LMA during the dark or subjective dark period between 24–48 h after IVM administration, reducing the CI in LD by 77.7 ± 5.7% (VEH p=0.001, **Ext. Fig. 7B-D**), and in DD by 80.7 ± 5% (p=0.002, **Ext. Fig. 7E-G**). The Tb during the dark period or presumptive dark period was lower after IVM administration when compare vs VEH (which includes the effect of the handling in contrast to the baseline), therefore, the CI of Tb was significantly reduced in both LD and DD, 24–48h after IVM administration by 77.7 ± 8.1% in LD (p=0.005, **Ext. Fig. 7H-J**), and by 68.3 ± 14% in DD (p=0.004, **Ext. Fig. 7K-M**). These data suggest that DMH^Vgat^ neurons participate in the circadian peak of Cort release at the beginning of the active period (as seen in DD), likely reducing the inhibitory tone to the PVH^CRH^ at this temporal point, but that in LD light can act as a cue for elevation of Cort at the onset of the active period, presumably through another pathway. Also, the DMH^Vgat^ neurons play a larger role than DMH^Vglut2^ neurons in the circadian regulation of LMA, as the ablation of DMH^Vgat^ but not the DMH^Vglut2^ neurons dramatically reduced LMA during the dark or subjective dark periods.

### The activation of DMH GABAergic neurons increases Cort levels, whereas cvPVH GABAergic neurons constrain Cort levels

As the ablation of the DMH^Vgat^ neurons prevented the peak in DD conditions, we tested whether the activation of those neurons can induce Cort release. We placed injections of *AAV8-DIO-hM3Dq-mCherry* in the DMH of *Vgat-ires-Cre* mice. From 8 mice injected, 6 mice were confirmed to have injection sites covering at least 70% of the DMH bilaterally (Fig. 5A–B). CNO administration at the beginning of the light phase (ZT2) elevated the Cort levels to 36.8 ± 9 ng/ml one hour after administration, while Cort was only 10.2 ± 2 ng/ml after vehicle (p=0.016, Fig. 5C). Although activation of DMH^Vglut2^ neurons produced a larger increase in Cort levels (>150 ng/ml) than activation of the DMH^Vgat^ neurons, in both cases the increase in Cort levels caused by CNO was greater than the circadian peak of Cort in undisturbed littermates. Thus, activation of either DMH^Vgat^ and DMH^Vglut2^ neurons is capable of elevating Cort levels within the range achieved by circadian rhythms.

Because activation of the DMH^Vgat^ neurons is expected to release GABA and inhibit the postsynaptic targets, we hypothesized that the DMH^Vgat^ neurons induce Cort release by disinhibiting the PVH^CRH^ neurons through inhibitory relay neurons, similar to the mechanism by which GABAergic neurons in the arcuate nucleus increase Cort secretion during food deprivation ^15^. To understand the circuit through which DMH GABAergic neurons promote CRH secretion, we crossed *Vgat-ires-Cre* mice with *CRH-Venus* reporter mice to generate *Vgat-ires-Cre::CRH-Venus* mice. We then injected the DMH of 4 *Vgat-ires-Cre::CRH-Venus* mice with *AAV8-DIO-ChR2-mCherry*. These injections were relatively small (3–9 nl compared to 24–45 nl for the other experiments) and covering mostly the rostral part of the DMH (rDMH). We found that axons and terminals labeled anterogradely (expressing mCherry, in red) from rDMH^Vgat^ neurons largely avoided PVH^CRH^ neurons (green). The bulk of the labeled terminal field was in areas surrounding the PVH, as well as in the region medial and ventral to the PVH^CRH^ neurons and extending into the caudal ventral PVH (Fig. 5D–E, 5J–K). Whereas the PVH contains very few GABAergic cells, many are found in the region surrounding the PVH, known as the peri-PVH area, as well as in the adjacent caudal ventral part of the PVH. As terminals from the DMH^Vgat^ neurons blanket the caudal ventral PVH as well as the adjacent peri-PVH just outside it, for the sake of simplicity we will refer to this region as the cvPVH. Local inhibitory inputs within the PVH have been described from this region ^16–18^, so we decided to explore whether the cvPVH^Vgat^ neurons might mediate disinhibition of the PVH^CRH^ neurons during the circadian peak of Cort. We injected 3 *Vgat-ires-Cre::CRH-Venus* mice with *AAV8-DIO-ChR2-mCherry* in the PVH. The injection covered the ventral and lateral region of the PVH including the cvPVH, and labeled axons ramified inside the PVH, making appositions with the CRH-Venus neurons (Fig. 5F–G). To explore whether the cvPVH^Vgat^ neurons make monosynaptic contacts with PVH^CRH^ neurons, we combined conditional retrograde rabies tracing from PVH^CRH^ neurons with *in situ* hybridization for *Vgat* mRNA using the *CRH-ires-Cre* mice (*n*=2). We observed doubly labeled neurons in the cvPVH suggesting monosynaptic GABAergic inputs from this region to the PVH^CRH^ neurons (Fig. 5H–I). We then investigated whether the DMH^Vgat^ neurons innervate the cvPVH^Vgat^ neurons. Following injections of *AAV8-DIO-ChR2-mCherry* into the rostral DMH of *Vgat-ires-Cre::L10-GFP* mice (*n*=2) we found dense terminal labelling in apposition to the cvPVH^Vgat^ neurons, suggesting direct connectivity (Fig. 5J–K). These neuroanatomical findings support the hypothesis of a circuit where the DMH^Vgat^ neurons disinhibit PVH^CRH^ neurons through direct inhibition of the cvPVH^Vgat^ neurons (DMH^Vgat^ → cvPVH^Vgat^ → PVH^CRH^).

To determine whether the cvPVH^Vgat^ neurons play a role in the circadian release of Cort, we ablated the Vgat expressing neurons in the cvPVH by placing injections of *AAV10-hSyn-mCherry-DIO-DTA* in the cvPVH of *Vgat-ires-Cre* mice (*n*=5). In all cases, the injection sites marked by mCherry expression involved the cvPVH, where Vgat cells projecting to the PVH^CRH^ neurons are found, and the PVH, where few Vgat cells are seen. (Fig. 5L–M, **Ext. Fig. 8A**). After four weeks to allow full expression of DTA, we recorded LMA and Tb in the mice for 12 days in both LD and DD, and we collected blood samples for Cort measurements toward the end of both the LD and DD periods. The cvPVH^Vgat^ ablated mice showed higher levels of Cort but only at the beginning of the active phase, from 21.3 ± 2.3 ng/ml in controls to 59.8 ± 11.3 ng/ml in cvPVH^Vgat^ ablated mice in LD (p<0.001, Fig. 5N), and from 25.6 ± 5.4 ng/ml to 51.3 ± 8.7 ng/ml in DD (p<0.001, Fig. 5O). The CI of Cort also increased by 180.2 ± 52.9% in LD (p=0.01) and 100.4 ± 33.9% in DD (p=0.036, Fig. 5P). No major changes were observed in LMA or Tb with ablation of cvPVH^Vgat^ neurons (**Ext. Fig. 8B-Q**). These data indicate that the cvPVH^Vgat^ neurons play an important role in inhibiting Cort secretion, even limiting its peak during the daily surge in Cort secretion at CT13. DMH^Vgat^ inhibition of cvPVH^Vgat^ neurons partially disinhibits the PVH^CRH^ neurons, allowing the surge in Cort secretion at CT13, but completely eliminating the cvPVH^Vgat^ neurons allows even greater stimulation of the PVH^CRH^ cells at that time by DMH^Vglut2^ inputs.

### Dissecting the synaptic inputs of the DMH^Vgat^→ cvPVH^Vgat^→ PVH^CRH^ circuit

To dissect the synaptic mechanisms by which the DMH^Vgat^ neurons control the PVH^CRH^ neurons, we injected the DMH of a new cohort of seven *Vgat-ires-Cre::CRH-Venus* mice with *AAV8-DIO-ChR2-mCherry*. We then performed patch clamp recordings in brain slices from green-labelled PVH^CRH^ neurons while photoactivating the red DMH^Vgat^ terminals in the PVH (Fig. 6A). Our rabies virus injections of the PVH^CRH^ neurons had shown direct inputs from GABAergic neurons in the caudal DMH, and as expected in four mice with injections covering the most caudal part of the DMH, the photostimulation of the DMH^Vgat^ input evoked inhibitory synaptic responses in most of the PVH^CRH^ neurons (data not shown). However, in three mice where the injection site was restricted mostly to the rostral part of the DMH (rDMH), the photostimulation of the DMH^Vgat^ input evoked inhibitory synaptic responses in only 4 out of 17 recorded PVH^CRH^ neurons (Fig. 6B–E, R).

Our experiments in which we disabled the DMH^Vgat^ neurons throughout the DMH (Fig. 4) reduced the circadian peak of Cort secretion, and when we activated these neurons Cort secretion was increased (Fig. 5A–C). These results are consistent with the predominant effect of DMH^Vgat^ neurons on the circadian rhythm of Cort secretion being to increase the surge at the beginning of the active period. Thus, it is likely that the direct input from caudal DMH^Vgat^ neurons to PVH^CRH^ neurons is involved in some other aspect of Cort regulation, but not in circadian rhythms of Cort secretion. We therefore hypothesized that rDMH^Vgat^ neurons, which provide few direct inhibitory inputs to the PVH^CRH^ cells might promote the circadian increase in Cort secretion by disinhibiting PVH^CRH^ neurons via a GABAergic intermediate neuronal population (Fig. 5F–K).

To investigate whether rDMH^Vgat^ neurons directly inhibit the cvPVH^Vgat^ neurons, we expressed *AAV8-DIO-ChR2-mCherry* in the rDMH of 5 *Vgat-ires-Cre::L10-GFP mice*. We then recorded from GFP-labelled cvPVH^Vgat^ neurons while stimulating the putative rDMH^Vgat^ input to them (DMH^Vgat^ →cvPVH^Vgat^) (Fig. 6F). Photostimulation evoked opto-inhibitory postsynaptic currents (oIPSCs) in 14 out of 15 recorded cvPVH^Vgat^ neurons. These oIPSCs depended on GABA_A_ receptor-mediated signaling, as they were blocked by bicuculline (Fig. 6G–M). Furthermore, oIPSCs persisted in the presence of TTX (1μM) and 4-AP (200 μM) indicating monosynaptic connectivity (*n*=6, Fig. 6N).

Collectively our study using single light pulse stimulation of the rDMH^Vgat^ input demonstrated that rDMH^Vgat^ neurons directly inhibit cvPVH^Vgat^ neurons while mainly sparing the PVH^CRH^ neurons. When we activated the rDMH^Vgat^ terminals at different frequencies while recording from the PVH^CRH^ neurons neurons in which single light pulses did not evoke oIPCS, we found that photostimulation of the rDMH^Vgat^ input at 5 or 10 Hz caused a trend toward reduction in the number of spontaneous IPSCs, which became statistically significant at 20 Hz (*n*=7; Fig. 7A–E). These results indicate that the rDMH^Vgat^ neurons can disinhibit most PVH^CRH^ neurons by reducing their GABAergic afferent input.

Our conditional tracing study and CRACM recordings suggest that the rDMH^Vgat^ input disinhibits the PVH^CRH^ neurons at least in part through the GABAergic neurons located in the cvPVH. We therefore tested whether the cvPVH^Vgat^ neurons can directly inhibit the PVH^CRH^ neurons (cvPVH^Vgat^ → PVH^CRH^). We placed *AAV8-DIO-ChR2-mCherry* injections in the cvPVH of 3 *Vgat-ires-Cre::CRH-Venus mice*. We then recorded from labelled PVH^CRH^ neurons while photostimulating cvPVH^Vgat^ neurons and terminals. Photostimulation of cvPVH^Vgat^ neurons evoked oIPSCs in all PVH^CRH^ neurons recorded (*n*=14). These oIPSCs were mediated by GABA_A_ receptor signaling as they were blocked by bicuculline (*n*=4) and were resistant to TTX+4-AP, indicating monosynaptic connectivity (*n*=6) (Fig. 7F–L). Interestingly, in four cases where the ChR2 expression was more dorsal and lateral involving the portion of the pPVH just lateral to the PVH only about half of the PVH^CRH^ neurons we recorded from showed oIPSCs (*n*=4) (Fig. 7M–R).

Taken together, these results indicate that rDMH^Vgat^ (rDMH^Vgat^) neurons can disinhibit PVH^CRH^ neurons through GABAergic neurons located in the cvPVH (rDMH^Vgat^ → cvPVH^Vgat^ → PVH^CRH^), promoting the circadian increase of Cort levels at CT13.

## Discussion

This study identifies two parallel circuits from the DMH that participate in circadian regulation of the activity of PVH^CRH^ neurons and therefore the release of Cort. The first circuit involves the direct excitation of the PVH^CRH^ neurons by DMH^Vglut2^ neurons (DMH^Vglut2^→ PVH^CRH^), whereas the second circuit involves a polysynaptic disinhibition of PVH^CRH^ neurons by rDMH^Vgat^ neurons through the intermediary cvPVH^Vgat^ neurons (rDMH^Vgat^ → cvPVH^Vgat^ → PVH^CRH^). These circuits participate in the circadian rhythms of Cort secretion in a complementary way. The direct input from the DMH^Vglut2^ neurons to the PVH^CRH^ cells appears to be necessary to maintain the daily peak in Cort under both LD and DD whereas the rDMH^Vgat^ disinhibitory circuitry appears to be necessary for the daily increase in Cort levels at CT13 only during constant dark conditions. This suggests that the rDMH^Vgat^ neurons mediate the disinhibition of PVH^CRH^ neurons in anticipation of the circadian active period, but that under LD conditions the daily light signal itself can activate circuitry to disinhibit PVH^CRH^ neurons at ZT13 even in the absence of the DMH^Vgat^ circadian input. This is similar to the “masking” phenomenon by which there is a reduction in LMA during the light phase of LD, even in mice in which the SCN has been ablated and which have no circadian rhythm of LMA in DD ^19–21^. The origin of this light-induced signal for elevation of Cort at the beginning of the active period is not known, but apparently does not involve the DMH^Vgat^ neurons.

This points out a *limitation in our study*. Our work was designed to isolate circuitry that contributes to the circadian regulation of Cort secretion in anticipation of the active period. However, Cort is a well-known stress hormone, and we did not examine the circuits responsible for elevating Cort levels after psychological or physical stressors, which can often exceed the daily circadian peak by many times. Even our methods for acquiring the Cort samples were designed to minimize stress-induced elevation of Cort, as all samples were taken within 1 minute of touching the animal. The DMH is thought to participate in stress responses, including the elevation of ACTH and Cort during stress ^9,10,22–24^. We did not investigate the role of the DMH^Vglut2^ and DMH^Vgat^ neurons in causing Cort elevation during stress, but it is noteworthy that the levels of Cort after chemogenetically activating the DMH^Vglut2^ neurons were in the range typically seen during stress responses (>150 ng/ml), whereas the peak levels seen after chemogenetic activation of the DMH^Vgat^ neurons were <40 ng/ml. This lower level of Cort secretion may reflect the fact that both rDMH^Vgat^ neurons which disinhibit PVH^CRH^ neurons and caudal DMH^Vgat^ neurons that inhibit CRH cells were being activated at the same time. If the caudal DMH^Vgat^ neurons, for example, were involved in stress responses, one would expect them to be inhibited (not activated) during stress. The caudal DMH^Vgat^ neurons do not appear to play a major role in circadian rhythms of Cort secretion (e.g., suppressing Cort levels during the inactive period), because DMH^Vgat^ ablations had little effect on Cort levels at ZT1 or 7 (Fig. 5N). Future experiments should endeavor to find unique genetic signatures for the different classes of DMH^Vgat^ neurons ^25^, and to test their roles in both circadian and stress-induced secretion of Cort.

Another limitation of our study was that while we examined the sources of circadian input from the DMH to the PVH^CRH^ neurons, our output measure was serum Cort levels. While CRH secretion is the dominant influence on secretion of ACTH by the pituitary gland, other hormones, such as arginine vasopressin may be secreted into the hypophysial portal circulation and can contribute to ACTH secretion ^26^. However, *Crh−/−* mice, despite having normal circadian rhythms of LMA, lack (males) or have minimal (females) circadian increases in Cort secretion ^27^. Thus, the focus on the PVH^CRH^ neurons as the main driver of Cort rhythms is justified.

It is also important to point out that the roles of the DMH^Vgat^ and DMH^Vglut2^ neurons in the circadian regulation of Cort secretion appear to depend upon release of GABA and glutamate, respectively, because deletion of just the *Vgat* or *Vglut2* genes from neurons in the DMH had similar effects to abating the entire neuron population. Many DMH neurons also express peptides, including galanin, dynorphin, neuropeptide Y, CART, orexin, and many others ^28–32^. While the different patterns of gene expression by DMH neurons may help us to classify them and provide key marker genes for neurons that participate in particular roles or have specific projections, it does not appear that the DMH neurons depend upon peptidergic transmission to produce the circadian pattern of Cort secretion.

In this work, we also were able to dissect the role of the DMH^Vglut2^ vs DMH^Vgat^ neurons in the global reduction of LMA and Tb and dramatic reduction in the circadian rhythm of LMA reported by Chou et al. (2003) after non-specific ablation of the DMH in rats. First, ablation of the DMH^Vgat^ neurons reduced the amount of LMA by about half across the entire dark or presumptive dark cycle, whereas ablation of the DMH^Vglut2^ neurons reduced LMA a bit less but predominantly during the transitions between the light and dark periods. The combination would explain the low levels of LMA with almost complete loss of circadian rhythms of LMA reported by Chou et al. after non-specific DMH cell ablation. The ablation of either the DMH^Vgut2^ or DMH^Vgat^ neurons reduced Tb mainly during the dark and presumptive dark phase when the animals are most active. This effect is consistent with the known role of dorsal hypothalamic/DMH glutamatergic neurons in promoting thermogenesis and heat conservation. DMH^Vgat^ neurons have been hypothesized to inhibit the DH/DMH^Vglut2^ neurons, thus would be expected to cause a rise, not fall in Tb. However, there are probably a number of classes of DMH^Vgat^ neurons which may have opposing effects on Tb, and the reduction in LMA may have contributed to the lower Tb. As our experiments only assayed the effects of the entire population of DMH^Vgat^ neurons, it will be important to examine the role of the different classes of DMH^Vgat^ neurons, which may be involved in a variety of physiological response, in regulating Tb, as well as LMA and Cort secretion.

### Synergistic regulation of Cort by the DMH Glutamatergic and GABAergic populations

Previous work by Chou et al. had shown that non-specific ablation of the DMH in rats prevented the daily increase of Cort levels associated with the active cycle and reduced Cort secretion to the level normally measured during the inactive period when the animals are mainly asleep ^8^. Because the effect of the DMH was to increase Cort levels, we initially hypothesized that the circadian input from the DMH to the PVH^CRH^ neurons was likely to be excitatory, i.e., glutamatergic. In this study we tested that hypothesis and observed that the DMH^Vglut2^ neurons play an important role in the circadian elevation of Cort at CT13 but that ablation of the DMH^Vglut2^ neurons did not completely eliminate the circadian surge in Cort. We therefore tested the role of DMH^Vgat^ neurons were surprised that they also participate in the circadian elevation of Cort, particularly in DD, at CT13.

In retrospect, the synergistic cooperation of DMH^Vgat^ and DMH^Vglut2^ neurons in the circadian regulation of Cort secretion probably should not have been a surprise. It has been estimated that about half of the synapses onto the PVH^CRH^ neurons are glutamatergic and the other half GABAergic ^14^. Similarly, we found in our rabies virus experiments that about half of the DMH neurons projecting to PVH^CRH^ neurons are glutamatergic and half are GABAergic. The DMH^Vgat^ neurons with direct projections to the PVH^CRH^ cells were found predominantly in the caudal part of the DMH in both our rabies virus and CRACM experiments, but these neurons, which would inhibit the secretion of CRH, do not appear to play a role in the DMH regulation of circadian rhythms of Cort secretion. Rather, rDMH^Vgat^ neurons are important for the daily surge in Cort secretion in anticipation of the active period, via inhibition of GABAergic interneurons and thus disinhibiting of PVH^CRH^ neurons

These observations caused us to examine the origins of the GABAergic inputs to PVH^CRH^ neurons. The PVH contains very few GABAergic neurons within its borders, whereas the pPVH is rich in GABAergic neurons and it has been proposed that the pPVH^Vgat^ neurons could provide tonic inhibition of the PVH^CRH^ neurons and function as a “brake” upon their secretion of CRH ^18^. On the other hand, other studies have found that some of these inputs derive from neurons that are distant from the PVH. For example, a recent study from Douglass and colleagues reported that the elevation of Cort levels during fasting depends upon AgRP neurons in the arcuate nucleus inhibiting tonically active GABAergic afferents from the bed nucleus of the stria terminalis to the PVH^CRH^ neurons, thus disinhibiting them ^15^. In the case of circadian secretion of Cort, we found that the daily peak in secretion in anticipation of the active phase relies in part upon input from DMH^Vgat^ cells to cvPVH^Vgat^ neurons, to disinhibit PVH^CRH^ neurons.

In support of the contribution of both the DMH^Vglu2^ and rDMH^Vgat^ inputs to the circadian peak of Cort, the surge in Cort levels at CT13 was greater after the ablation of the cvPVH^Vgat^ neurons than in control animals, suggesting that the tonic activity of cvPVH^Vgat^ neurons places a brake on CRH secretion that is only partially lifted during the circadian daily peak. This GABAergic tone holds back the level of activity of the PVH^CRH^ neurons that otherwise would be driven by the DMH^Vglut2^ circadian input. This interaction suggests that GABAergic inputs to the PVH^CRH^ neurons may be important in limiting Cort secretion under other physiological conditions, and that disinhibition may be a common motif in their regulation. Interestingly, we found that cvPVH^Vgat^ neurons provided a particularly rich source of input to the PVH^CRH^ cells compared to the lateral pPVH. It would be useful to know whether disinhibition of the cvPVH^Vgat^ neurons plays a role in other physiological situations that increase Cort secretion.

### Origin of the circadian signal to the DMH^Vglut2^ →PVH^CRH^ and DMH^Vgat^ →cvPVH^Vgat^ →PVH^CRH^ pathways

Although the activity of the DMH^Vglut2^ and DMH^Vgat^ circadian inputs to the PVH^CRH^ neurons are temporally aligned in control of the circadian rhythm of Cort, neither is in phase with the transitions between the light and dark periods. In fact, in mice the activity of PVH^CRH^ neurons peaks about 6 hr and the Cort levels rise about 3 hr prior to the onset of the active (dark or presumptive dark) cycle, and reach a peak shortly after that cycle begins, only to fall back to low levels by halfway through the active cycle ^33,34^. Similarly, in humans the cortisol levels begin to rise about halfway through the habitual sleep cycle and are about twice as high at 8am as they are at 4pm ^35^. As indicated by our rabies virus tracing experiments (**Ext. Fig. 4B-D**), and previous observations by others ^4,5,34^, there are few if any direct projections from the SCN to the PVH^CRH^ neurons. Jones et al. suggest that SCN^VIP^ inputs to regions of the PVH that are nearby the CRH neurons may influence their firing via volume transmission. However, our data indicate that such inputs are insufficient to cause the daily circadian surge in Cort secretion in the absence of DMH inputs. The offset of the timing of the increase in Cort secretion from the light-dark cycle suggests that there is further circuitry downstream of the SCN that converts its signal, which is tied to the light-dark cycle, into a timing signal that starts the increase in Cort several hours prior to the onset of the light period. In addition, in nocturnal (mice) and diurnal (humans) species the onset of Cort secretion bears the same time relationship to onset of the active phase, even though their relationships to the light cycle are 180 degrees out of phase with each other.

We hypothesize that a multi-synaptic circuit from the SCN to the subparaventricular zone (SPZ) and then the DMH is a likely site for this interaction, as the ventromedial SPZ receives a large portion of the SCN output, and projects heavily to the DMH in both rats and mice ^6,11,36,37^. Most SPZ neurons have activity patterns that are in antiphase to the SCN (i.e., are more active during the dark period) and they are almost uniformly GABAergic ^38,39^. The SPZ also receives inputs from a wide range of other hypothalamic cell groups. Thus, it is possible that various configurations of polysynaptic connections in the SPZ might allow it to produce timing signals that are driven by the SCN but not in phase with it. A previous study in which we examined the effect of deletion of the *Vgat* gene from SPZ neurons in five mice found that it had variable impact on the circadian rhythm of Cort secretion among individual animals, but that this was not statistically significant across the group ^37^. However, the location of these injections varied, and it is possible that SPZ neurons may use other neurotransmitters than GABA. Further study of the origin of the circadian timing signal to the DMH may prove fruitful in understanding how the SCN signal is used to optimally time various physiological processes.

## Material and methods

### Animals.

Because adult female mice lose their circadian rhythm of Tb during estrus and we needed to record circadian rhythms across many days, we used only male adult mice, aging 12–16 weeks old. The strain of the mice were *Vgat-ires-Cre* ^41^ (JAX: 016962), *Vglut2-ires-Cre* ^41^ (JAX: 016963), *CRH-ires-Cre* ^42^ (JAX: 012704), *CRH-VenusΔNeo* ^43^, *Vglut2*^*loxP/loxP*^ (JAX:036439), *Vgat*^*loxP/loxP* 44^ (JAX: 012897) and *R26-loxSTOPlox-L10-GFP* mice ^42^. Mice were individually housed under 12–12h light/dark cycle unless the protocol specified otherwise. Room temperature were controlled in a range between 22 ± 2°C and free access to food and water was provided. All procedures were performed in accordance with the National Institutes of Health Guide for the Care and Use of Laboratory Animals, and formal approval of our protocols was obtained from the Institutionary Animal Care and Use Committee at Beth Israel Deaconess Medical Center. All precautions were taken to minimize pain and discomfort in the mice.

### Viral Vectors used.

*AAV10-hSyn-mCherry-DIO-DTA* which conditionally expresses the subunit A of diphtheria toxin in a Cre-dependent fashion and the mCherry protein in non-Cre cells (acquired from Patrick M Fuller) ^45,46^ was injected (~24nL) in *Vgat-ires-Cre or Vglut2-ires-Cre* mice. *AAV8-eSYN-EGFP-T2A-iCre* containing the genes for Cre and EGFP under the neuron eSYN promoter (VB1089, Vector Biolabs, PA, US; 8.6X10^11^ viral genomes ml^−1^) was bilaterally injected (~24nL) in *Vglut2*^*loxP/loxP*^ and *Vgat*^*loxP/loxP*^ mice. *AAV8-Ef1a-DIO-ChR2(H134R)-mCherry* injections (UNC, addgene #AV9080, 1.4X10^13^ viral genomes ml^−1^) were used for the neuroanatomical and CRACM experiments (~3–9 nL) in *Vgat-ires-Cre or Vglut2-ires-Cre* mice. *AAV10-DIO-hGlyR-mCherry* (acquired from Patrick M Fuller) ^12,24,37^ injections were made in the DMH (~45nL) either in *Vglut2-ires-Cre* or *Vgat-ires-Cre* mice. *AAV10-EF1α-DIO-hM3Dq-mCherry* (UNC, addgene #50460 virus core; 2×10^13^ viral genomes ml^−1^) was injected (~30nL) either in *Vglut2-IRES-Cre* or *Vgat-IRES-Cre* mice. *AAV8-CAG-DIO-GFP* (~24nl) was injected as a control virus in specified experiments (UNC; addgene #59331 7X10¹² viral genomes ml^−1^). ~45nL of *AAV8-Ef1a-DIO-TVA-mCherry* (UNC Vector Core; 1.13X10^12^ viral genomes ml^−1^) mixed 1:1 with *AAV8-CAG-DIO-rabiesG* (Stanford Vector Core; 3.4X10^12^ viral genomes ml^−1^) was injected unilaterally, and 21 days later, ~55nL of *EnvA-ΔG-rabies-GFP* (Salk Viral Vector Core; 1.51X10^8^ viral genomes ml^−1^) were administrated in the PVH of *CRH-ires-Cre* mice. All the experiments and recordings started four weeks after the injections to allow a complete transfection of the AAVs.

### Surgeries.

Mice were deeply anesthetized with a mix of ketamine/xylazine (100/10 mg/kg, i.p.). Radiotelemetry sensors (TA-F10, DSI, US) for body temperature (Tb) and locomotor activity (LMA) were implanted in the peritoneal cavity with a small abdominal incision. The coordinates for the stereotactic microinjections of viral vectors in the brain, from bregma were: DMH, AP= −1.75mm, ML= ±0.25mm, DV= −4.9mm; PVH, AP= −0.8mm, ML= +0.25mm, DV= −4.8mm; cvPVH, AP= −0.8mm, ML= +0.25mm, DV= −4.85mm ^40^. Mice receive analgesic treatment with meloxicam post-surgery. All procedures were performed under aseptic conditions.

### LMA and Tb continuous recordings.

Average Tb and LMA were recorded every 5 min during all the protocol using the radiotelemetry DSI system. The signal from the telemetry probes, previously implanted, was received and converted with the PhysioTel HD and PhysioTel (DSI) hardware. The LMA and Tb data were analyzed using the software ClockLab Analysis version 6 (Acticmetrics).

### Plasma sampling and corticosterone immunoassay.

Blood samples (~20μl) were obtained in a microvette tube (CB300, Sarstedt, US) after a small incision in the tail in less than 1 min after onset of handling the mouse, centrifuged at 10,000 rpm for 10 min, and the plasma was collected and stored at −40°C. Samples were collected with a minimum interval of 30 hours between samples. Under the DD protocol, the first sample was obtained on the third day after the lights turned off at CT13 and then every 30 hours until we completed the 4 time points. ELISA assay for corticosterone was performed as per the manufacturer instructions (ADI-901–097, Enzo life science, US) and the corticosterone signal read with a 405nm filter (iMark, Bio-Rad, US). Each sample was read in duplicates.

### Perfusion and Immunohistochemistry.

At the end of each protocol, animals were deeply anesthetized with 7% chloral hydrate (0.015ml/gr of body weight i.p.) and perfused transcardially with 30ml of PBS followed by 30ml of 10%-buffered formalin (Thermo Fisher Scientific, US). Brains were extracted and postfixed in 10%-buffered formalin overnight, cryoprotected in 30% sucrose solution, sectioned at 40μm coronal sections (three series) and stored at 4°C in PBS with sodium azide. Brain sections were washed three times (5 min each) and then blocked for 1 hour with 3% normal horse serum (diluted in 0.4% Triton X-100 in PBS). Primary antibody was incubated overnight in same solution as blocking, at room temperature and continuous agitation. Primary antibody used were Chicken anti-GFP (Invitrogen; 1:5,000, A10262), Rat anti-mCherry (Invitrogen; 1:4,000, M11217), Mouse anti-NueN (Millipore; 1:3,000, MAB377). Then, the sections were washed six times (5 min each) in PBS, and incubated with secondary antibodies for 2 h in blocking solution at room temperature and continuous agitation. The secondary antibodies used were Alexa fluor 488-conjugated Donkey anti-Chicken (Jackson; 1:200, AB2340375), Alexa fluor 555-conjugated Goat anti-Rat (Invitrogen; 1:200, A21434), Alexa fluor 488-conjugated Goat anti-Mouse (Jackson; 1:200, AB2338840). Then, sections were washed three times (10 min each) in PBS, mounted on electrostatically treated slides and coverslipped with VECTASHIELD Antifade Mounting Medium with DAPI (Vector Laboratories) for microscope visualization and image acquisition.

### RNA scope in situ hybridization.

Two of the brains from the *EnvA-ΔG-rabies-GFP* experiments were sectioned at 20μm and tissue was mounted on glass slides in RNAase-free conditions. Slices were dried and *in situ* hybridization was performed using RNAScope multiplex fluorescent reagent kit V2 (catalog #323100, Advanced Cell Diagnostics, US). Slices were pretreated with hydrogen peroxide for 10 minutes at room temperature and subjected to target retrieval step for 5 min in a steamer (>99°C), This was followed by dehydration in 90% alcohol and air-dried for 5 min. Sections were then treated with protease III at 40°C for 30 min. Then, the slices were rinsed with sterile water and incubated for 2 hrs either with the *Vglut2* probe (RNAscope Probe- Mm-Slc17a6; catalog #319171, Advanced Cell Diagnostics, US) or Vgat Probe (RNAscope Probe- Mm-Slc32a1; catalog # 319191, Advanced Cell Diagnostics, US) at 40°C for RNA hybridization. This is followed by incubation with amplification reagents, AMP1, AMP2 (30 min each) and AMP3 (15 min) at 40°C. Sections were then incubated with HRP -C1 for 15 mins and Cy5 fluorophore (catalog #NEL741001, PerkinElmer) for 30 mins at 40°C. Finally, HRP blocker was added to the sections for 15 min at 40°C. After each step sections were washed with 1 x wash buffer provided in the kit. Slides were then dried and coverslipped with Vectashield antifade mounting medium (catalog #H-1400, Vector Laboratories) for microscope visualization and image acquisition.

### Image acquisition, gradient maps and rabies transfected counting.

Coronal sections were scanned at 20X magnification using an Olympus VS120 slide-scanning microscope or at 63X magnification using confocal microscope (Leica Stellaris 5). Fluorescence images were taken using Olympus OlyVIA software (3.4.1) or Leica application suite (version 4.2.1). The injection site of each animal was confirmed by the expression of mCherry or EGFP. Microphotographs were transformed to a gray scale and the background were subtracted. The images at the same anatomical level were superimposed with 30% transparency to build a gradient map using GIMP software (version 2.10.34). In the brain sections from the rabies experiment, we counted cell bodies in at least 3 levels for each nucleus (anterior, medial and posterior). In the sections used for *in situ* hybridiazation of rabies infected cells, we counted cell bodies and double stained cells in 6 levels of the DMH. Cell body counting was conducted manually using the multipoint tool in Image J software (version 1.54). Because the number of infected cells varied considerably from animal to animal and we were not trying to establish the absolute number of cells that project to the PVH^CRH^ neurons, we did not attempt to correct the numbers for cell size.

### ChR2-assisted circuit mapping (CRACM).

For electrophysiology experiments, *AAV8-DIO-ChR2-mCherry* (~3–9 nL) was injected into the DMH of *Vglut2-ires-Cre::CRH-Venus* mice (*n*=4) or *Vgat-ires-Cre::CRH-Venus* mice (*n*=3) or *Vgat-ires-Cre::L10-GFP mice* (*n=5*) or into the cvPVH of *Vgat-ires-Cre::CRH-Venus* mice (*n*=7). Mice with AAV injections placed outside of the target were excluded from the analysis. Four to six weeks following the AAV injections, mice were deeply anaesthetized with isoflurane (5% in oxygen) via inhalation and transcardially perfused with ice-cold ACSF (N-methyl-D-glucamine, NMDG-based solution described below). The mouse brains were then quickly removed and sectioned coronally (250 μm-thickness) in ice-cold NMDG-based ACSF using a vibrating microtome (VT1200S, Leica). We first incubated the slices containing the PVH for 5 min at 37°C, then transferred them into a holding chamber at 37°C containing ACSF (Na-based solution) for 10 minutes. We let the brain slices gradually return to room temperature (~1 hour) before starting recording. Brain slices were recorded in a recording chamber, where were submerged, and perfused with Na-based ACSF (described below, 1–1.5 ml/min). We recorded PVH^CRH^ and cvPVH^Vgat^ neurons identified by Venus or L10-GFP (green) fluorescence, respectively, using a combination of fluorescence and infrared differential interference contrast microscopy (IR-DIC). We used a fixed stage upright microscope (BX51WI, Olympus America) equipped with a Nomarski water immersion lens (Olympus 40X / 0.8 NAW) and IR-sensitive CCD camera (ORCA-ER, Hamamatsu) to acquire real time images using Micro-Manager software. We recorded the neurons in whole-cell voltage clamp and current clamp configurations using a Multiclamp 700B amplifier (Molecular Devices), a Digidata 1322A interface, and Clampex 9.0 software (Molecular Devices). Neurons showing a greater than 10% change in input resistance over the duration of the recording were excluded from the analysis. We photostimulated the DMH or cvPVH axons in the PVH using a full-field (~10 mW/mm^2^, 1 mm beam width) 5W LUXEON blue light-emitting diode (470 nm wavelength; #M470L2-C4; Thorlabs), coupled to the epifluorescence pathway of the microscope. We stimulated photo-evoked excitatory postsynaptic currents (oEPSCs) or inhibitory postsynaptic currents (oIPSCs) with 10ms light pulses (0.1 Hz, for a minimum of 30 trials). In the DMH^Vgat^ injected mice, we also tested the effects of photostimulation on the action potential firing of cvPVH^Vgat^ neurons and on the spontaneous IPSCs (sIPSCs) of PVH^CRH^ neurons using train stimulations (train-duration: 60s, frequency: 5, 10 and 20 Hz, light pulse duration: 10 ms). Action potential firing and oEPSCs (holding potential = −70 mV) were recorded in ACSF and using a K-gluconate-based pipette solution. oIPSCs (holding potential = 0 mV) were recorded in ACSF containing 1 mM kynurenic acid and using a Cs-methane-sulfonate-based pipette solution. To record photo-evoked synaptic events in the presence of synaptic blockade, we bath applied tetrodotoxin (TTX; 1 μM) and the potassium channel blocker, 4-AP (200–500 μM). For all recordings we added 0.5% biocytin in the pipette solutions to mark the recorded neurons. The recorded slices containing the PVH and cvPVH and the slices containing the DMH, where the AAVs were injected, were fixed overnight in 10%-buffered formalin for *post hoc* histological and anatomical assessment. To label and map the recorded neurons filled with biocytin, immediately after the *in vitro* recordings, we fixed the recorded slices in 10% buffered formalin, washed them, and incubated them overnight in streptavidin-conjugated Alexa Fluor 405 (1:500; Cat#: S32351; Invitrogen, Thermo Fisher Scientific Waltham, MA) ^47,48^. We acquired images using a Leica Stellaris 5 confocal microscope using a 63X oil immersion objective.

### Solutions for CRACM experiments.

NMDG-based ACSF solution containing (in mM): 100 NMDG, 2.5 KCl, 1.24 NaH_2_PO_4_, 30 NaHCO_3_, 25 glucose, 20 HEPES, 2 thiourea, 5 Na-L-ascorbate, 3 Na-pyruvate, 0.5 CaCl_2_, 10 MgSO_4_ (pH 7.3: 95% O_2_ and 5% CO_2_; 310–320 mOsm). Na-based ACSF solution contained (in mM): 120 NaCl, 2.5 KCl, 1.3 MgCl_2_, 10 glucose, 26 NaHCO_3_, 1.24 NaH_2_PO_4_, 4 CaCl_2_, 2 thiourea, 1 Na-L-ascorbate, 3 Na-pyruvate (pH 7.3–7.4 in 95% O_2_ and 5% CO_2_; 310–320 mOsm). Cs-methane-sulfonate-based pipette solution containing (in mM): 125 Cs-methane-sulfonate, 11 KCl, 10 HEPES, 0.1 CaCl_2_, 1 EGTA, 5 Mg-ATP and 0.3 Na-GTP (pH adjusted to 7.2 with CsOH, 280 mOsm). K-gluconate-sbased pipette solution containing (in mM): 120 K-Gluconate, 10 KCl, 3 MgCl2, 10 HEPES, 2.5 K-ATP, 0.5 Na-GTP (pH 7.2 adjusted with KOH; 280 mOsm). We purchased tetrodotoxin and kynurenic acid from Cayman Chemical (Ann Arbor, MI), bicuculline methiodide from Tocris Bioscience (Ellisville, MO). We purchased all other chemicals from Fisher Scientific (Waltham, MA) or Sigma-Aldrich (Saint Luis, MO).

### Data and statistical analysis for CRACM experiments.

Recording data were analyzed using Clampfit 10 (Molecular Devices), MiniAnalysis 6 software (Synaptosoft), customized Python scripts (Python 3, www.python.org) and MatLab (version R2020B; MathWorks; Natick, MA) software. Figures were generated using Igor Pro version 6 (WaveMetrics), Prism 7 (GraphPad, La Jolla, CA), Inkscape (GitLab) and Photoshop (Adobe) software. To ensure unbiased detection of synaptic events, the EPSCs and IPSCs were detected and analyzed automatically using MiniAnalysis. We considered EPSCs or IPSCs in PVH^CRH^ neurons and cvPVH^Vgat^ neurons to be photo-evoked if their probability during the first 50 ms following the light pulses was greater than the IPSC probability + five times the standard error of the mean (SEM) before the light stimulation and if their latency was within ± 1 ms of the median value of E/IPSC latencies calculated for each neuron within the first 50 ms following photostimulation. We calculated the latency of the photo-evoked EPSC and IPSCs as the time difference between the start of the light pulse and the 5% rise point of the first synaptic event ^48^. Group means were compared using *paired* t-tests. Cumulative distributions were compared using the *Kolmogorov-Smirnov* test*.* Values indicating p < 0.05 were considered significant.

### Statistical analysis.

Data sets were tested to see whether they fulfilled the parametric criteria of Brown-Forsythe (homo/heteroscedasticity) and Shapiro Wilk (fit to normal distribution). Corticosterone plasma levels and circadian distribution of LMA and Tb were analyzed with a *two-way ANOVA* with a factor for group and a factor for time, followed by a *post-hoc* multiple comparisons *Sidak* test. CI for Cort was calculated by the difference between the mean ZT13 and ZT1 levels and divided by the ZT13-ZT1 mean in each mouse, then normalized to 100% by the mean difference in the Control group. CI for LMA and Tb were calculated by the difference between the mean dark (or presumptive dark) and light (or presumptive light) levels divided by the 24h total counts for LMA and 24h mean for Tb, then normalized to 100% against the mean for the Control group. Light or Dark mean for LMA and Tb were evaluated with *two-way ANOVA* with a factor for group and a factor for LD vs DD, followed by a *post-hoc* multiple comparisons *Tukey* test. When the same mice were evaluated as its own control for LMA and Tb (pre- and post-DTA experiments, and the hGlyR experiments), we evaluate them with *Repeated Measures [RM] two-way ANOVA* with a factor for group and a factor for LD vs DD, followed by a *post-hoc* multiple comparisons *Tukey* test. CI and amplitude data were compared by *unpaired* t-test analysis, or *one-way ANOVA*, followed by a *post-hoc* multiple comparisons *Tukey* test in the cases where included baseline in the analysis. Actograms, periodograms and amplitude of the cosinor fitting were built using ClockLab ActiMetrics software version 6.1.02. Statistical analyses and graphs were performed with the GraphPad Prism software version 8. Data are represented as mean ± SEM. Significance values α were set at p<0.05.
