# A hypothalamic circuit for circadian regulation of corticosterone secretion

**DOI:** 10.21203/rs.3.rs-4718850/v2

**Published:** 2025-06-18

**Authors:** Oscar D. Ramirez-Plascencia, Roberto De Luca, Natalia L. S. Machado, Dominique Eghlidi, Mudasir A. Khanday, Sathyajit S. Bandaru, Francesca Raffin, Nina Vujovic, Elda Arrigoni, Clifford B. Saper

**Affiliations:** 1. Department of Neurology, Division of Sleep Medicine, Beth Israel Deaconess Medical Center, Boston, MA 02215, USA.; 2. Division of Sleep Medicine, Harvard Medical School, Boston, MA 02215, USA.; 3. Departments of Medicine and Neurology, Brigham and Women’s Hospital, Boston, MA 02115, USA.; 4. Department of Biology and Biotechnology “Lazzaro Spallanzani”, University of Pavia, Pavia, PV 27100, Italy.

**Keywords:** corticosterone, corticotropin-releasing hormone, dorsomedial hypothalamus and paraventricular hypothalamus

## Abstract

The secretion of cortisol in humans and corticosterone (Cort) in rodents follows a daily rhythm which is important in readying the individual for daily activity. This rhythm is orchestrated by the suprachiasmatic nucleus (SCN), but how it ultimately regulates the circadian rhythm of activity of neurons in the paraventricular nucleus of the hypothalamus that produce corticotropin-releasing hormone (PVH^CRH^ neurons) is not known. We hypothesized that the SCN may exert this influence by projections to the subparaventricular zone (SPZ), which in turn innervates neurons in the dorsomedial nucleus of the hypothalamus (DMH) that regulate PVH^CRH^ neurons. First, we found that ablating SPZ^Vgat^ neurons eliminates the circadian rhythm of Cort secretion, but that deleting *Vgat* from them does not, suggesting that they predominantly use some other transmitter. Next, we found that either ablating or acutely inhibiting the DMH glutamatergic (DMH^Vglut2^) neurons resulted in a 40–70% reduction in the daily peak of Cort. Deletion of the *Vglut2* gene within the DMH produced a similar effect, highlighting the indispensable role of glutamatergic signaling. Chemogenetic stimulation of DMH^Vglut2^ neurons led to an increase of Cort levels, and optogenetic activation of their terminals in the PVH in hypothalamic slices directly activated PVH^CRH^ neurons through glutamate action on AMPA receptors (the DMH^Vglut2^ → PVH^CRH^ pathway). Similar to the disruption of DMH^Vglut2^ neurons, ablating, inhibiting, or disrupting GABA transmission by DMH GABAergic (DMH^Vgat^) neurons diminished the circadian peak of Cort, particularly under constant darkness conditions. Chemogenetic stimulation of rostral DMH^Vgat^ neurons increased Cort, although with a lower magnitude compared to DMH^Vglut2^ neuron stimulation, suggesting a role in disinhibiting PVH^CRH^ neurons. Supporting this hypothesis, we found that rostral DMH^Vgat^ neurons project directly to GABAergic neurons in the caudal ventral part of the PVH and adjacent peri-PVH area (cvPVH), which directly inhibit PVH^CRH^ neurons, and that activating the rostral DMH^Vgat^ terminals in the cvPVH in brain slices reduced GABAergic afferent input onto the PVH^CRH^ neurons. Finally, ablation of cvPVH^Vgat^ neurons resulted in increased Cort release at the onset of the active phase, affirming the pivotal role of the DMH^Vgat^ → cvPVH^Vgat^ → PVH^CRH^ pathway in Cort secretion. In summary, our study delineates two parallel pathways transmitting temporal information to PVH^CRH^ neurons, collectively orchestrating the daily surge in Cort in anticipation of the active phase. These findings are crucial to understand the neural circuits regulating Cort secretion, shedding light on the mechanisms governing this physiological process and the coordinated interplay between the SCN, SPZ, DMH, and PVH.

## Graphical abstract

*Model Proposed for the hypothalamic circuitry involved in the circadian regulation of Cort release.* During the first part of the light period, the DMH^Vglut2^ and DMH^Vgat^ neurons are inhibited by the SCN, allowing a tonic inhibition of the PVH^CRH^ neurons from the cvPVH^Vgat^ neurons (left panel). Starting the second part of the light phase thought the first hours of dark period, the Cort levels gradually increases by direct DMH^Vglut2^ → PVH^CRH^, and a parallel rDMH^Vgat^ → cvPVH^Vgat^ → PVH^CRH^ disinhibitory circuit (right panel).

## Introduction

PVH^CRH^ neurons play a key role in the secretion of corticosteroids by releasing CRH into the hypothalamic-hypophysial portal circulation to stimulate the anterior pituitary release of adrenocorticotropic hormone (ACTH). ACTH then stimulates the adrenal cortex to secrete cortisol in humans and corticosterone (Cort) in rodents. The secretion of Cort follows a daily rhythm, with peak levels typically occurring just before the onset of the active phase, thereby regulating behavior, metabolism, and immune response. Trough levels occur toward the onset of the sleep phase, reflecting the natural dip in hormone secretion during the body’s resting period ^1,2^. This rhythmic secretion pattern reflects the intricate coordination of various neural and hormonal pathways involved in circadian regulation. Loss of the circadian rhythm of Cort secretion in rats after lesions of the suprachiasmatic nucleus (SCN) provided some of the first evidence for the role of the SCN as the brain’s master biological clock ^3^. However, the mechanism by which the SCN influences Cort secretion remains unknown. The SCN sends only limited axons to the PVH, where they mainly terminate in subregions of the PVH that control autonomic activity ^4,5^ and spare the PVH^CRH^ neurons. Indeed, the bulk of SCN efferents terminate in an arc stretching caudally and dorsally from the SCN, encompassing the subparaventricular zone (SPZ) and rostral dorsomedial nucleus of the hypothalamus (DMH). ^6,7^. SPZ neurons are predominantly GABAergic^8–10^, and lesion studies have shown that the ventral SPZ participates in the circadian regulation of the sleep-wake cycle, locomotor activity (LMA) and Cort release, while the dorsal part is involved in rhythms of body temperature (Tb) regulation^11,12^. Downstream, non-specific ablation of DMH neurons in rats eliminates the daily peak in Cort secretion, reducing the overall daily levels ^13^. Stimulation of the DMH increases ACTH and Cort release ^14,15^. These findings led us to hypothesize that the daily peak in Cort could be regulated by the SCN through successive relays in the SPZ and the DMH, that, in turn, stimulates the PVH^CRH^ neurons to trigger Cort release^16^.

To test this model, first we evaluated the role of SPZ^Vgat^ neurons in transmitting the temporal information from the SCN to regulate the daily Cort release by selectively ablating them or preventing their GABA release. Then, as the DMH is a heterogeneous region containing roughly equal proportions of glutamatergic and GABAergic neurons, we examined the roles of both of these DMH cell types in the circadian regulation of Cort levels. We tested whether the DMH^Vglut2^ neurons were necessary for the circadian regulation of Cort by either ablating or inhibiting them or deleting the *Vglut2* gene in the DMH, preventing glutamate release. We then tested the effect of chemogenetic activation of DMH^Vglut2^ neurons on Cort levels and used Channelrhodopsin-assisted circuit mapping (CRACM) to determine the synaptic effects of DMH^Vglut2^ → PVH^CRH^ input. We also assessed the effect of the activation, as well as ablation or inhibition of the DMH^Vgat^ neurons, and whether deleting the *Vgat* gene in the DMH affected the daily rhythm of Cort release. Finally, we examined the role of the caudal ventral part of the PVH and adjacent peri-PVH area GABAergic (cvPVH^Vgat^) neurons in disinhibiting the PVH^CRH^ neurons through a polysynaptic circuit (DMH^Vgat^→ cvPVH^Vgat^→ PVH^CRH^).

## Results

### SPZ^Vgat^ neurons, but not GABA release, are necessary for the circadian regulation of Cort.

**Figure 1.**
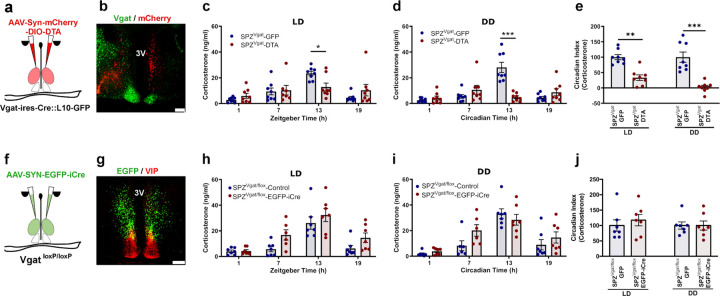
SPZ^Vgat^ neurons, but not GABA release, are necessary for maintaining the circadian rhythm of Cort secretion. (**a**) Schematic drawing of SPZ^Vgat^ neuron ablation by DTA (**b**) Representative image of a section from an animal with SPZ^Vgat^ neuron ablation, showing GABAergic neurons (in green, native signal) and the injection site in red (non-DMH^Vglut2^ neurons expressing mCherry, native signal). Notice the remaining green GABAergic neurons in the SCN, and the almost complete elimination of GABAergic neurons just dorsally in the SPZ. Red mCherry was expressed by non-GABAergic cells in the injection site. (**c**) Ablation of SPZ^Vgat^ neurons prevented the daily Cort increase at ZT13 in LD (*Two-way ANOVA*; *Tukey’s* multiple comparisons test. ZT13 SPZ^Vgat^-GFP vs SPZ^Vgat^-DTA: *p=0.025) and (**d**) at CT13 in DD (*Two-way ANOVA*; *Tukey’s* multiple comparisons test. CT13 SPZ^Vgat^-GFP vs SPZ^Vgat^-DTA: ***p<0.001). **(e)** The circadian index (CI, for method of calculation of CI, see Statistical Analysis in the Materials and Methods) of Cort secretion was reduced by 66.6 ±9.1% in LD (*Unpaired t-test:* t=5.434, df=14, **p=0.002) and by 97.7 ±5.9% in DD (*Unpaired t-test*: t=5.534, df=14, ***p<0.001). (**f**) Schematic representation of *Vgat* gene deletion in the SPZ. (**g**) Representative micrograph showing EGFP expression in the SPZ neurons in which the *Vgat* gene was deleted (green), just dorsal to the SCN^VIP^ neurons (shown immunohistochemically in red). (**h**) Mice with *Vgat* gene deletion in the SPZ showed no change in the daily Cort peak either in LD or (**i**) DD photoperiod. (**j**) No changes were detected in the Cort CI. LD, Light:Dark photoperiod; DD, Constant darkness

To evaluate whether the SPZ GABAergic neurons participate in regulating circadian Cort release, we placed injections in the SPZ of *Vgat-ires-Cre::L10* mice of a viral vector that constitutively expresses mCherry in neurons lacking Cre-recombinase (Cre), but in cells that express Cre instead produces diphtheria toxin A (DTA), which induces cell death (AAV10-hSyn-mCherry-DIO-DTA). We placed injections of an inactive virus (*AAV8-DIO-EGFP*) in the same site in control mice. After four weeks to allow the full expression of DTA or GFP, we measured Cort levels in blood from DTA injected and control mice in a 12:12 light:dark (LD) cycle, by tail nicks at four time points (ZT 1, 7, 13, 19, taken in random order with at least 30 hrs between samplings), and at the same time points in constant darkness (DD) (CT 1, 7, 13, 19), starting on the third day at CT13, and then every 30 hours. Simultaneously, we recorded the circadian rhythms of locomotor activity (LMA) and body temperature (Tb) for 15 days in LD, and then 15 days under DD. From a total of 12 mice injected with DTA, 8 mice were identified by a blinded observer with bilateral DTA injections covering at least 70% of the SPZ; some injections involved parts of the SCN (<50%) on one side of the brain, but none involved the SCN bilaterally (Fig. 1a-b and Ext. Fig. 1a). The mice with injections that missed the target were considered anatomical controls. Within the injection site, as defined by mCherry expression in the non-Cre dependent cells, nearly all Vgat neurons, identified by their expression of L10-GFP, were ablated (Fig. 1b). The ablation of the SPZ^Vgat^ neurons reduced the Circadian Index (CI, comparing the levels during the dark vs the light period; for method of calculation of CI see Statistical Analysis in the Materials and Methods) of Cort secretion compared with the Ctrl animals by 66.6 ±9.1% in LD (p=0.008) and 97.7 ±5.9% in DD (p<0.001). The peak Cort level was reduced from control levels of 23.4 ± 1.7 ng/ml in LD and 28.1 ± 3.9 ng/ml in DD, to 12.8 ± 3.0 ng/ml in LD and 4.8 ± 1.1 ng/ml in DD in SPZ^Vgat^ ablated mice; (Fig. 1c-e). The SPZ^Vgat^ ablations also caused a dramatic reduction in the CI of locomotor activity (LMA) by 51.1 ±3.5% in LD (p<0.001) and by 79.7 ±4.8% in DD (p<0.001), mostly by reducing the general LMA during the (presumptive) dark period (Ext. Fig. 1b-i). The CI of body temperature (Tb) was reduced by 50.42 ± 5.6% in LD (p<0.001) and 58.34 ±6.5% in DD (p<0.001; Ext. Fig. 1j-q). The anatomical controls (missed injections) did not show any changes in LMA or Tb.

**Figure 2.**
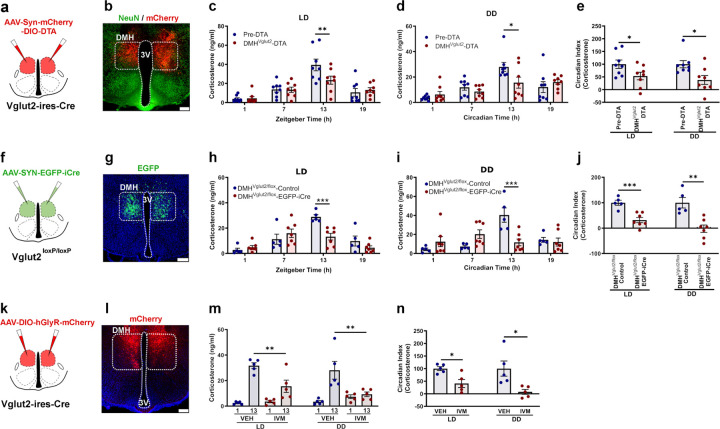
DMH^Vglut2^ neurons are necessary to maintain the endogenous circadian rhythm of Cort release. (**a**) Schematic of the DMH^Vglut2^ neuron ablation by DTA (**b**) Representative image of a section from an animal with DMH^Vglut2^ neuron ablation, showing the injection site in red (non-DMH^Vglut2^ neurons expressing mCherry) and green NeuN staining as a reference marker. (**c**) Ablation of DMH^Vglut2^ neurons reduced the circadian peak of Cort at ZT13 in LD (*Two-way ANOVA*; *Tukey’s* multiple comparisons test. ZT13 Pre-DTA vs DMH^Vglut2^-DTA: **p=0.007) and (**d**) in DD at CT13 (*Two-way ANOVA*; *Tukey’s* multiple comparisons test. CT13 Pre-DTA vs DMH^Vglut2^-DTA: *p=0.013). (**e**) The Cort circadian index (CI) in the DMH^Vglut2^ ablated mice was reduced by 46.6 ±14.2% in LD (*Paired t-test*: t=2.225, df=14, *p=0.043) and 62.0 ±17.3% in DD (*Unpaired* t-test: t=2.775, df=14,*p=0.014). (**f**) Schematic representation of *Vglut2* gene deletion in the DMH. (**g**) Representative micrograph showing EGFP expression in the DMH neurons in which the *Vglut2* gene has been deleted (in green). (**h**) *Vglut2* deletion in DMH diminished the Cort peak at ZT13 in LD (*Two-way ANOVA*; *Tukey’s* multiple comparisons test. ZT13 DMH^Vglut2/flox^-Control vs DMH^Vglut2/flox^-EGFP-iCre: ***p<0.001) and entirely prevented the peak at CT13 in DD (**i**; *Two-way ANOVA*; *Tukey’s* multiple comparisons test. CT13 DMH^Vglut2/flox^-Control vs DMH^Vglut2flox^-EGFP-iCre: ***p<0.001). (**j**) The Cort CI was reduced by 68.2 ±10.1% in LD (*Unpaired* t-test: t=4.621, df=10, ***p<0.001) and by 102.1 ±14.6% in DD (*Unpaired* t-test: t=4.167, df=10, **p=0.001). (**k**) Schematic of the chemogenetic inhibition of DMH^Vglut2^ neurons expressing hGlyR by IVM administration. (**l**) Representative micrograph of hGlyR-mCherry expression in the DMH (in red). (**m**) IVM administration diminished the Cort rise at ZT13 in LD (*Two-way ANOVA*; *Tukey’s* multiple comparisons test. ZT13 VEH vs IVM: **p=0.006) and almost entirely prevented it at CT13 in DD compared with vehicle (*Two-way ANOVA*; *Tukey’s* multiple comparisons test. CT13 VEH vs IVM: **p=0.001). (**n**) The Cort CI was reduced one day after the administration of IVM by 58.9 ±15.9% in LD (*Paired t-test*: t=3.334, df=8, *p=0.01) and by 92.3 ± 9.9% in DD (*Paired t-test*: t=2.903, df=8, *p=0.019). In all cases we visualize the native signal except for the hGlyR signal that was enhanced with immunofluorescence for mCherry. LD, Light:Dark photoperiod; DD, Constant dark; Reference scale bar= 200µm; 3V, third ventricle.

To evaluate whether the temporal information was transmitted by GABA release from the SPZ^Vgat^ neurons, we placed injections in the SPZ of *AAV8-SYN-EGFP-iCre* (*AAV8-EGFP-iCre*) or a control virus (*AAV8-DIO-GFP*) in *Vgat*^*loxP/loxP*^ (*Vgat-flox*) mice to delete expression of functional Vgat protein and so to abolish GABA transmission from the transfected neurons. From 12 injected mice, 7 received a bilateral injection of *AAV8-EGFP-iCre* covering at least 70% of the SPZ without extending into the SCN (Fig. 1f-g and Ext. Fig. 2a). Surprisingly, deletion of Vgat from the SPZ neurons did not affect the circadian release of Cort (Fig. 1h-j). By contrast, the circadian rhythms of LMA and Tb were reduced by the deletion of *Vgat* from SPZ^Vgat^ cells, although to a lesser degree than after ablation of those neurons. For instance, the CI of LMA was reduced by 39.75 ± 8.9% in DD (p<0.001; Ext. Fig. 2b-i), and the CI of Tb was reduced by 25.7 ± 4.6% in LD (p=0.006) and 38.6 ± 7.3% in DD (p<0.001; Ext. Fig. 2j-q). Taken together, these data indicate that the SPZ^Vgat^ neurons are necessary to transmit temporal information for circadian regulation of Cort, LMA and Tb from the SCN, but the mechanism is mediated at least in part by transmitters other than GABA.

### Glutamate signaling by DMH^Vglut2^ neurons is necessary for the increase in Cort at the beginning of the active period.

**Figure 3.**
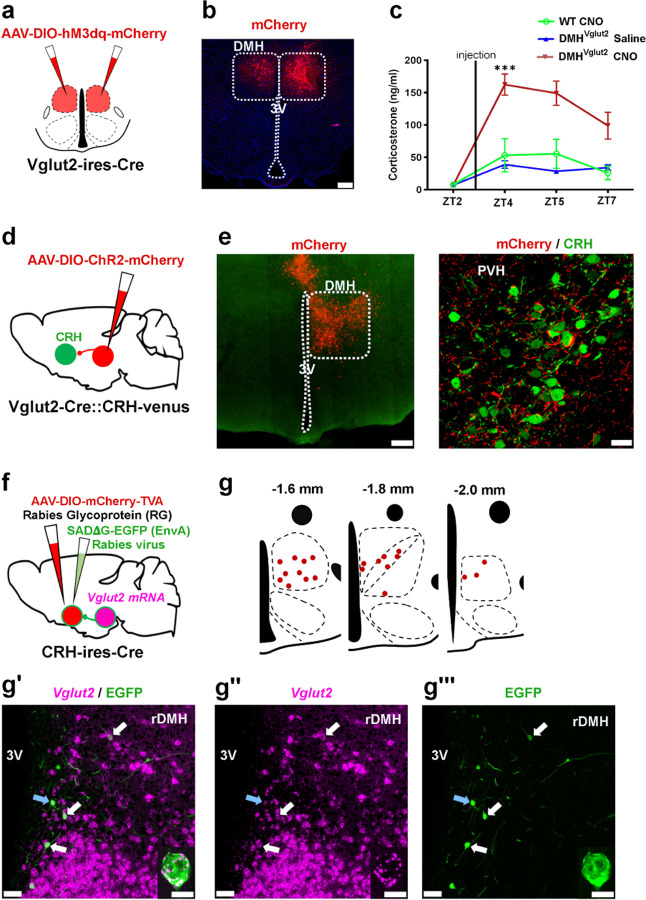
DMH^Vglut2^ neurons project to the CRH neurons of the PVH and their chemogenetic activation increases Cort. (**a**) Schematic of *AAV-DIO-hM3dq-mCherry* injection targeting the DMH^Vglut2^ neurons. (**b**) Representative image of an *AAV-DIO-hM3dq-mCherry* (in red) injection site in the DMH. (**c**) CNO-mediated chemogenetic-stimulation of DMH^Vglut2^ neurons boosted Cort to levels much higher than the usual daily peak (cf. Fig 2c; *Two-way ANOVA*; *Tukey’s* multiple comparisons test. CT4 WT CNO vs DMH^Vglut2^ CNO: ***p<0.001, CT4 DMH^Vglut2^ Saline vs DMH^Vglut2^ CNO: ***p<0.001). (**d**) Schematic of *AAV-DIO-ChR2-mCherry* injection targeting DMH^Vglut2^ neurons. (**e**) Representative photomicrograph of the DMH^Vglut2^ neurons expressing ChR2-mCherry (*left* panel, in red), and their appositions with PVH^CRH^ neurons (*right* panel, in green). (**f**) Schematic of the EnvA-rabies experiment to map the monosynaptic input from the DMH^Vglut2^ neurons to PVH^CRH^ neurons. (**g**) Mapping of the rabies-*Vglut2* co-labeling distribution at different rostro-caudal levels of the DMH, and representative images showing *Vglut2 mRNA* expression (g’ and g’’, in magenta) and rabies expression (g’ and g’’’, in green) within the rostral DMH (rDMH). The arrows point to the co-labeled cells, and the inset in the lower-right part shows a higher magnification of the neuron pointed by the blue arrow. The hM3dq and ChR2 signal were enhanced with immunofluorescence for mCherry, while the Rabies infected cells were enhanced using an EGFP antibody. Reference scale bar: in **b** and **e** (*left*)= 200 µm, **e** (*center*) and **g’-g’’’**= 50µm, in **g’-g’’’** insets = 10µm. 3V, third ventricle. Atlas levels correspond to Paxinos and Franklin Atlas ^47^.

As the DMH neurons are downstream of the SPZ and have been shown in rats to be necessary for the daily peak of Cort secretion, we hypothesized that DMH^Vglut2^ neurons might excite PVH^CRH^ cells in the circadian regulation of Cort. We first measured Cort levels in blood from 14 *Vglut2-ires-Cre* mice at the same four time points (ZT 1, 7, 13, 19) in a 12:12 light:dark (LD) cycle and in constant darkness (DD) (CT 1, 7, 13, 19). Simultaneously, we recorded the baseline circadian rhythms of LMA and Tb for 12 days in LD, and then 12 days under DD. We then placed injections into the DMH of *AAV10-hSyn-mCherry-DIO-DTA*, and after four weeks to allow full expression of DTA, we measured Cort levels in LD and DD as described, and recorded LMA and Tb in the mice for 12 days in LD and 12 days in DD. From a total of 14 mice, we identified 8 mice in which the injection covered at least 70% of the DMH bilaterally (Fig. 2a-b and Ext. Fig. 3a) and these mice were used for further analysis. DMH^Vglut2^ ablation reduced the Cort peak at the beginning of the active phase (ZT 13) from 39.4 ± 6 ng/ml in LD and 27.9 ± 3.5 ng/ml in DD before *AAV10-hSyn-mCherry-DIO-DTA* injections, to 23.4 ± 4.2 ng/ml in LD (p=0.007, Fig. 2c) and 15.5 ± 4.3 ng/ml in DD after DMH^Vglut2^ neuron ablation (p=0.013, Fig. 2d). The CI of Cort secretion was reduced by 46.6 ±14.2% in LD (p=0.043) and 62.0 ±17.3% in DD after the ablation (p=0.014, Fig. 2e). In one cohort of mice, we measured the response to stress of the mice before and after the ablation by subjecting them to 1 hr of restraint at ZT3. However, the Cort levels induced by restraint stress were comparable before and after the DTA injections, indicating that there was no impairment in overall Cort secretion and that DMH^Vglut2^ neurons do not play a role in restraint stress-induced Cort secretion (Ext. Fig. 3b). The ablation of DMH^Vglut2^ neurons also reduced the peak in LMA at the transitions between active and inactive periods both in LD and DD. Because these transition periods spanned the light and dark cycles, there was no change in the CI of LMA, although the amplitude of the LMA rhythm in DD was reduced in cosinor analysis (Ext. Fig. 3c-j). In contrast, ablation of DMH^Vglut2^ neurons reduced Tb during the light phase in LD and in both presumptive light and dark phases in DD. As a result, the CI and cosinor amplitude of the Tb rhythm was increased during LD but not DD (Ext. Fig. 3k-r).

**Figure 4.**
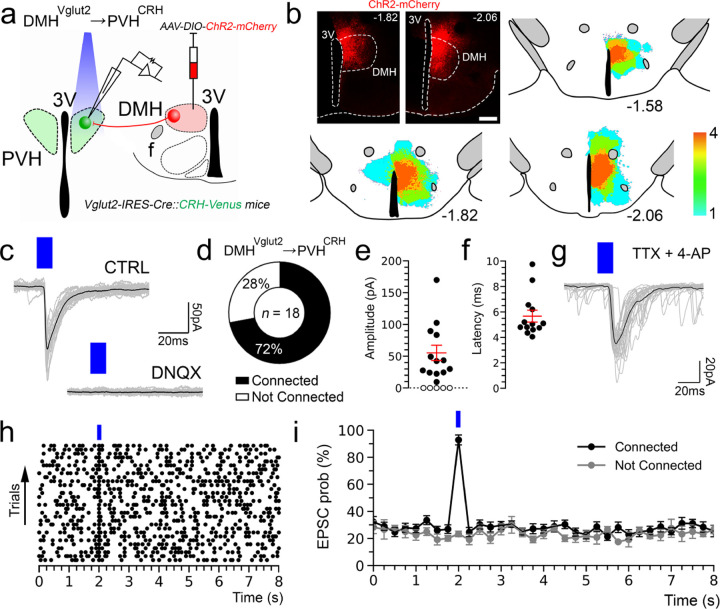
In vitro optogenetic stimulation of the DMH glutamatergic input directly excites PVH^CRH^ neurons. (**a**) Schematic representation of the recording to test the proposed DMH^Vglut2^ → PVH^CRH^ circuit; *Vglut2-ires-Cre::CRH-Venus mice* were injected with *AAV-DIO-ChR2-mCherry* in the DMH and recordings were conducted in brain slices from Venus-labeled PVH^CRH^ neurons while photostimulating the ipsilateral DMH^Vglut2^ input (the DMH is shown on the opposite side of the brain here for ease of illustration). (**b**) An example of ChR2-mCherry native expression after an injection in the DMH (*top left*) and density plots of the *AAV-DIO-ChR2-mCherry* injection sites (*n*=4 mice; *right* and *bottom*). (**c**) AMPA-mediated opto-evoked excitatory post-synaptic currents (oEPSCs) recorded in PVH^CRH^ neurons (*upper trace)* and blockade by AMPA receptor antagonist DNQX, 200µM (*lower trace*; *n*=4, from 2 mice). (**d**) Percentages of PVH^CRH^ neurons responding (*Connected*) and not responding (*Not Connected*) to photostimulation of the DMH^Vglut2^ input (*n*=18 PVH^CRH^ recorded neurons, from 4 mice). (**e**) Amplitude (filled markers, cells responding to photostimulation, *n*=13, open markers, cells not responding to photostimulation, *n*=15 neurons from 4 mice; mean and ± SEM of responding neurons) and (**f**) latency of oEPSCs in PVH^CRH^ neurons in response to photostimulation of the DMH^Vglut2^ input (mean and ± SEM; *n*=13 neurons from 4 mice). (**g**) oEPSCs in PVH^CRH^ neurons recorded in TTX 1µM + 4-AP 500µM (*n*=6 neurons from 2 mice) indicating monosynaptic connectivity. (**h**) Raster plot of EPSCs in a representative PVH^CRH^ neuron with photostimulation of the DMH^Vglut2^ → PVH^CRH^ input (bin duration: 50ms). (**I**) EPSC probability in response to photostimulation of the DMH^Vglut2^ → PVH^CRH^ input (black, *n*=13 neurons; grey, *n*=5 neurons, from 4 mice). Reference scale bar: in b = 250 µm. f, fornix, 3V, third ventricle. Atlas levels correspond to Paxinos and Franklin Atlas ^47^.

We then evaluated whether the effect of ablation of DMH^Vgut2^ neurons on Cort rhythms was due to the loss of glutamate transmission, as opposed to other possible transmitters expressed in DMH^Vgut2^ neurons. We therefore injected the DMH of *Vglut2*^*loxP/loxP*^ (*Vglut2-flox*) mice with either *AAV8-EGFP-iCre* or a control virus *AAV8-DIO-GFP* and recorded LMA, Tb and Cort levels in both LD and DD. Seven of the 11 mice had injections covering at least 70% of the DMH bilaterally and were compared with 5 mice with *AAV8-DIO-GFP* injections in the DMH of *Vglut2-flox* mice as controls (Fig. 2f-g, Ext. Fig. 4a). In these animals, the Cort levels at ZT13 were reduced in LD from 28.9 ± 1.9 ng/ml to 13.2 ± 2.6 ng/ml (p<0.001, Fig. 2h) and in DD from 40.4 ± 7.5 ng/ml to 11.7 ± 3.5 ng/ml in DMH^Vglut2/flox^-Control and DMH^Vglut2/flox^-EGFP-iCre mice, respectively (p<0.001, Fig. 2i). The CI of Cort was reduced by 68.2 ± 10.1% in LD (p<0.001) and by 102.1 ± 14.6% in DD (p=0.001, Fig. 2j). Both the LMA and Tb were reduced during the dark and subjective dark phase, and at the transition from the dark to the light phase during LD and at the presumptive transition in DD (Ext. Fig. 4). However, the CI of neither LMA nor Tb was affected by the loss of glutamatergic signaling in the DMH.

**Figure 5.**
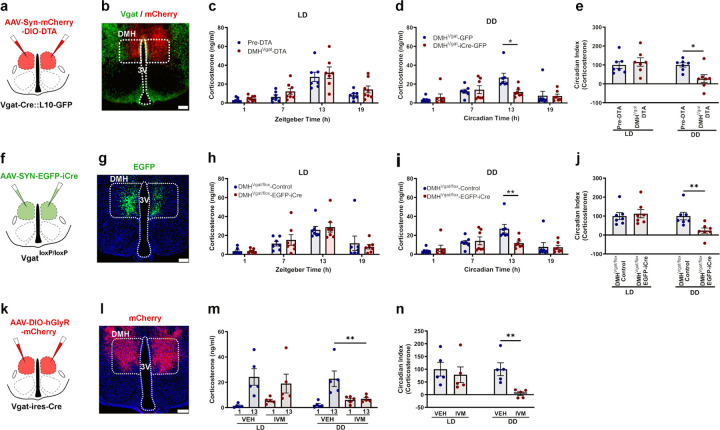
Ablation of DMH^Vgat^ neurons reduces the endogenous circadian peak of Cort release only in constant darkness. (**a**) Schematic representation of DMH^Vgat^ neuron ablation. (**b**) Representative micrograph showing GABAergic neurons (in green) and *AAV-DIO-DTA-mCherry* (in red) in the DMH of a *Vgat-ires-Cre::L10-GFP* mice. There were no remaining GABAergic neurons in the region of mCherry expression. (**c**) DMH^Vgat^ neuron ablation did not alter the daily Cort release in LD, (**d**) but eliminated it almost entirely under DD (*Two-way ANOVA*; *Tukey’s* multiple comparisons test. CT13 Pre-DTA vs DMH^Vgat^-DTA: *p=0.014). (**e**) The Cort CI was not significantly changed by DMH^Vgat^ ablation in LD, but was reduced by 72.4 ±21.3% in DD. (*Paired t-test*: t=3.028, df=12, *p=0.01). (**f**) Schematic representation of *Vgat* gene deletion in the DMH. (**g**) Representative photomicrograph showing the EGFP expression in *Vgat*-deleted neurons (green). (**h**) *Vgat* deletion from DMH neurons did not change the circadian regulation of Cort release in LD, (**i**) but eliminated the daily peak of Cort at CT13 in DD (*Two-way ANOVA*; *Tukey’s* multiple comparisons test. CT13 DMH^Vgat/flox^-Control vs DMH^Vgat/flox^-EGFP-iCre: **p=0.006). (**j**) The CI of Cort secretion was not affected by *Vgat* gene deletion in the DMH in LD but was reduced by 76.6 ±13.6% in DD in DMH^Vgat^-deleted mice (*Unpaired t-test*: t=3.160, df=12, **p=0.008). (**k**) Schematic representation of the *AAV-DIO-hGlyR-mCherry* injection in the DMH of *Vgat-ires-Cre* mice. (**l**) Representative micrograph of *hGlyR-mCherry* expression (in red) in the Vgat neurons of the DMH. (**m**) IVM chemo-inhibition of the DMH^Vgat^ neurons by hGlyR did not alter the Cort levels in LD, but prevented the peak at CT13 in DD (*Two-way ANOVA*; *Tukey’s* multiple comparisons test. CT13 VEH vs IVM: **p=0.006). (**n**) The Cort CI was reduced by 95.7 ±6.9% in DD (*Paired t-test*: t=3.641, df=8, **p=0.006). In all cases we visualize the native signal except for the hGlyR signal that was enhanced with immunofluorescence for mCherry. Reference scale bar= 200µm; 3V, third ventricle.

As the DMH^Vglut2^ ablation and the *Vglut2* mRNA deletion are chronic lesions that might be affected by compensatory mechanisms, we decided to test the effect of acute inhibition of the DMH^Vglut2^ neurons on Cort, LMA and Tb rhythms. We injected the DMH in 9 *Vglut2-ires-Cre* mice with a viral vector containing a modified human alpha-1 glycine receptor (*AAV10-DIO-hGlyR-mCherry*) which is insensitive to glycine but has 100-fold increased sensitivity to the antiparasitic drug ivermectin (IVM) compared to the unmutated glycine receptor^17^. Because the half-life of IVM is about 72 hrs, the injection of IVM induces long-lasting neuronal inhibition (4–5 d). To allow the drug to achieve a stable concentration in the CNS, we examined Cort levels between 24 and 48 hrs after drug administration ^18^ compared with injection of vehicle. We administered vehicle (VEH) or IVM (5 mg/kg, ip) one hour after the light or subjective light onset and sampled the mice the next day at ZT1 and ZT13 to evaluate the Cort levels; one week later, they received the opposite treatment. In 5 mice in which expression of mCherry was seen in at least 70% of the DMH bilaterally (Fig. 2k-l, Ext. Fig. 5a), the Cort levels at ZT13 under LD were reduced from 31.7 ± 2.3 ng/ml after VEH to 15.6 ± 4.8 ng/ml after IVM (p=0.006), and under DD from 28.1 ± 6.9 ng/ml after VEH to 9.3 ± 1.8 ng/ml after IVM (p=0.001, see Fig. 2m). The inhibition of the DMH^Vglut2^ neurons with IVM reduced the Cort CI by 58.9 ± 15.9% in LD (p=0.01) and by 92.3 ± 9.9% in DD (p=0.019, see Fig. 2n). The average CI of LMA after IVM administration was reduced by 103.3 ± 21.1% in LD (i.e., the day-night difference was reversed due to higher levels of LMA during the light period) (p=0.011, Ext. Fig. 5b-d), and was reduced in DD by 81.3 ± 9.1% when compared with vehicle (p=0.016, Ext. Fig. 5e-g) mainly due to the shifting of much of the LMA peak from the early dark period to the light period, however, the total LMA was not significantly different at 24–48h after IVM both in DD and LD (Ext. Fig. 5b-g). IVM also induced a decrease in Tb during the dark period, with a reduction of 0.5 ± 0.2°C observed in LD compared with vehicle administration (p=0.024, Ext. Fig. 5h-j). In DD conditions, the decrease in Tb during presumptive night was 0.4 ± 0.1°C (p=0.014, Ext. Fig. 5k-m). Tb and LMA returned to normal around day 5 after IVM either in LD or DD. The Tb CI was reduced in LD by 38.4 ± 6.2% after administration of IVM (p=0.025, Ext. Fig. 5j). In all the 3 experiments (DTA, iCre and hGlyR), the anatomical controls (animals with injections that missed the target) failed to show the responses observed in LMA or Tb.

These data clearly show that glutamatergic transmission by the DMH^Vglut2^ neurons plays an important role in the increase of Cort levels at the beginning of the active phase. The ablation or inhibition of DMH^Vglut2^ neurons reduces the peak of Cort to the early morning levels. The DMH^Vglut2^ neurons also increase LMA in a crepuscular pattern (at the transitions between the light and dark periods) and their loss causes a small (approximately 0.3–0.5 °C) decrease in Tb, but has little if any effect on the circadian rhythm of Tb.

### Glutamatergic DMH neurons send monosynaptic projections to the PVH^CRH^ neurons and boost Cort levels

As the ablation of DMH^Vglut2^ neurons reduced the circadian peak of Cort levels, we hypothesized that activation of these neurons may elevate the Cort levels in preparation for the active phase. First, we injected the DMH of 15 *Vglut2-ires-Cre* mice with *AAV10-EF1α-DIO-hM3Dq-mCherry* (Fig. 3a-b). Five to six weeks later, the mice were injected i.p. with clozapine-N-oxide (CNO) 0.3 mg/kg at ZT3. The acute activation of DMH^Vglut2^ neurons caused an increase in Cort levels from 7.4 ± 2.1 ng/ml just before the animals received the dose of CNO, to 162.5 ± 16.4 ng/ml 1 hr after the administration of CNO (p<0.001, see Fig. 3c). This increase is around three times higher than the Cort levels detected in the same mice that received saline or in WT mice injected with CNO. These results confirm that activation of the DMH^Vglut2^ neurons boosts Cort release.

**Figure 6.**
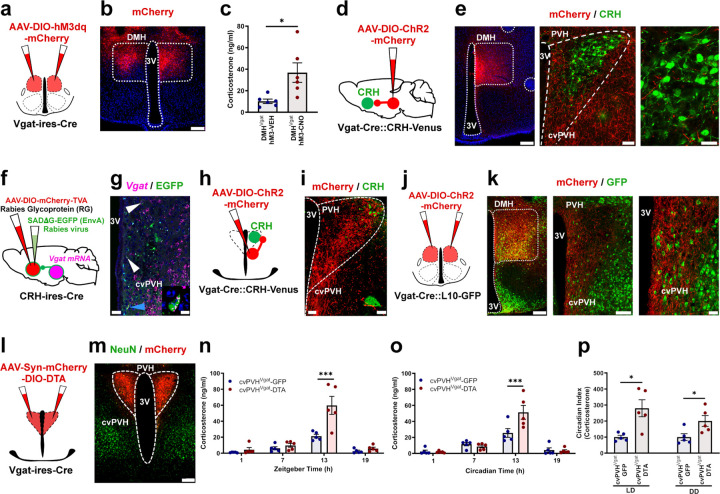
Chemo-activation of DMH^Vgat^ neurons elevates Cort levels through cvPVH GABAergic neurons. (**a**) Schematic of *AAV-DIO-hM3dq-mCherry* injections in the DMH in Vgat-ires-Cre mice. (**b**) Representative micrograph of hM3dq-mCherry expression (red) in the DMH. (**c**) Chemogenetic activation of the DMH^Vgat^ neurons increased Cort levels from 10.2 ±2 ng/ml after VEH to 36.8 ±9 ng/ml after CNO administration (*Paired t-test:* t=2.875, df=10, *p=0.016). (**d**) Schematic of an *AAV-DIO-ChR2-mCherry* injection in the DMH of a Vgat-Cre::CRH-Venus mice. (**e**) Representative micrograph of rDMH^Vgat^ neurons expressing ChR2-mCherry (in red, *left* panel), and their axon pattern in the PVH (*central* panel). Note that rDMH^Vgat^ axon terminals mostly avoided the PVH^CRH^ neurons (in green, *right* panel) but were dense in the ventral-medial and caudal portion of the PVH (cvPVH). (**f**) Schematic of the EnvA-Rabies experiment to map monosynaptic inputs from the cvPVH^Vgat^ neurons to PVH^CRH^ neurons. (**g**) Representative photomicrograph showing the *Vgat mRNA* expression (in magenta) and the Rabies expression (in green) and doubly labeled neurons within the cvPVH. Arrows point to double labeled neurons; the blue arrow points to the neuron in the *lower-right* inset. (**h**) Schematic of an *AAV-DIO-ChR2-mCherry* injection in the cvPVH. (**i**) Representative image of the cvPVH^Vgat^ cell bodies expressing ChR2-mCherry (in red) in the ventral part of the PVH^CRH^ neuron field (in green). Their axons spread through the more dorsal PVH and form appositions with the PVH^CRH^ neurons (*lower*-*right* inset). (**j**) Schematic of the *AAV-DIO-ChR2-mCherry* injections in the DMH. (**k**) Micrograph showing the expression of ChR2-mCherry (in red) in the DMH^Vgat^ neurons (in green, *left* panel), and their projections and appositions to the cvPVH^Vgat^ neurons (*central and right* panel). (**l**) Schematic of the PVH^Vgat^ neuron ablation. (**m**) Representative image of the injection site showing mCherry expression (in red) in non-Vgat neurons in the PVH, and the loss of neurons detected by the reduction of NeuN (in green) in the cvPVH and along the PVH borders due to the ablation of the Vgat cells. (**n**) The cvPVH^Vgat^ ablation boosted the Cort increase at ZT13 in LD (*Two-way ANOVA*; *Tukey’s* multiple comparisons test. ZT13 cvPVH^Vgat^-GFP vs cvPVH^Vgat^-DTA: ***p<0.001), and (**o**) at CT13 in DD (*Two-way ANOVA*; *Tukey’s* multiple comparisons test. CT13 cvPVH^Vgat^-GFP vs cvPVH^Vgat^-DTA: ***p<0.001). (**p**) The Cort CI increased after the cvPVH^Vgat^ neuron ablation by 180.2 ±52.9% in LD (*Unpaired t-test:* t=3.336, df=8, *p=0.01) and 100.4 ±33.9% in DD (*Unpaired t-test:* t=2.512, df=8, *p=0.036). The hM3dq and ChR2 signal, but not in the DTA experiments, were enhanced with immunofluorescence for mCherry, while the Rabies infected cells were enhanced using an EGFP antibody. Reference scale bar: in **b, e, I, k,** and **m** = 200 µm, **e** (center), **g** and **k** (center) = 50µm, in **e** (right panel), **i** (right panel) and k (right panel) = 20µm, in **g** and **i** (insets)= 5µm; 3V, third ventricle.

To determine whether the DMH^Vglut2^ neurons make direct synaptic contacts onto the PVH^CRH^ neurons to activate them and so to increase Cort levels, we conducted conditional anterograde tracing and conditional monosynaptic retrograde rabies tracing studies. We first evaluated the projection pattern of DMH^Vglut2^ neurons using *Vglut2-ires-Cre* mice crossed with reporter mice that express the fluorescent protein Venus (green) in the CRH expressing neurons (*Vglut2-ires-Cre::CRH-Venus*). Following injection of *AAV8-DIO-ChR2-mCherry* into the DMH, we found a dense projection from DMH^Vglut2^ neurons to the PVH that formed appositions with the PVH^CRH^ neurons (Fig. 3d-e). To identify the locations of DMH^Vglut2^ neurons potentially forming direct connections with the PVH^CRH^ cells, we injected the PVH of 5 *CRH-ires-Cre* mice with an AAV expressing TVA avian receptor (*AAV8-Ef1a-DIO-TVA-mCherry*) and an AAV expressing rabies glycoprotein necessary for rabies viral transfection (*AAV8-CAG-DIO-rabiesG*) specifically in the PVH^CRH^ neurons. Twenty-one days later, to label and map the direct monosynaptic inputs to the PVH^CRH^ neurons, we injected a glycoprotein (G)-deleted rabies virus (RVdG) that expresses EGFP and is enveloped with the avian ASLV type A protein (EnvA), which utilizes the TVA receptor for cell entry (*EnvA-ΔG-rabies-EGFP*). This results in PVH^CRH^ neurons infected by both AAV and rabies viruses (starter cells) displaying both green and red fluorescent signals, while neurons expressing only EGFP-Rabies (green) were retrogradely labeled by the viral particles produced in the starter cells (Ext. Fig. 6a-b). Transneuronally infected neurons were seen in areas previously reported to project to the PVH such as the bed nucleus of the stria terminalis, preoptic area, arcuate nucleus, and DMH, and we also observed a large number of retrogradely labeled neurons within the PVH itself and its immediate surrounding areas (Ext. Fig. 6c-d)^19^. We found few, if any, EGFP-Rabies infected neurons in the SCN, which supports the hypothesis that circadian control of Cort secretion by the SCN requires intermediate relays such as the SPZ and DMH.

We also combined rabies tracing from PVH^CRH^ neurons with *in situ* hybridization for *Vglut2* mRNA. We found about half of retrogradely-EGFP labeled neurons in the DMH expressed *Vglut2*, confirming that DMH^Vglut2^ neurons are likely to directly innervate PVH^CRH^ cells. In particular, we observe that the largest number of retrogradely labeled neurons expressing *Vglut2* were located in the anterior portion of the DMH (Fig. 3f-g). By contrast, retrogradely labeled neurons that expressed *Vgat* mRNA were found throughout the DMH, but with a more caudal predominance (Ext. Fig. 6e-f), indicating that there is also a direct GABAergic input predominantly from the caudal DMH to the PVH^CRH^ neurons (see below).

### Stimulation of the DMH^Vglut2^ neurons directly excites PVH^CRH^ neurons

To test the input from the DMH^Vglut2^ neurons to the PVH^CRH^ neurons (DMH^Vglut2^ → PVH^CRH^) functionally, we conducted *in vitro* channelrhodopsin-2(ChR2)-assisted circuit mapping (CRACM) recordings. We injected the DMH with an *AAV8-DIO-ChR2-mCherry* in a new cohort of 4 *Vglut2-ires-Cre::CRH-Venus* mice and then three to four weeks later we recorded from Venus-labeled PVH^CRH^ neurons in brain slices while photo-stimulating ipsilateral axons and terminals of DMH^Vglut2^ neurons within the PVH (Fig. 4a,b). All recordings were performed in the PVH ipsilateral to the injection site during the daytime (ZT3–8), at the nadir of the daily Cort cycle^20^. Optogenetic stimulation of the DMH^Vglut2^ input evoked excitatory synaptic responses in 72% of the PVH^CRH^ neurons recorded (*n*=18). This effect was mediated by the release of glutamate and AMPA receptor signaling as the opto-evoked excitatory postsynaptic currents (oEPSCs) were blocked by the AMPA receptor antagonist DNQX (*n*=4; Fig. 4c-f). Furthermore, the probability of oEPSCs in the PVH^CRH^ cells peaked about 5 msec after the photostimulation of the DMH^Vglut2^ terminals and the oEPSCs persisted in the presence of TTX (TTX 1µM) and 4-aminopyridine (4-AP), 200–500 µM) indicating monosynaptic connectivity (*n*=6 out of 8; Fig. 4f,g). These experiments demonstrate that DMH^Vglu2^ neurons have robust direct synaptic input to PVH^CRH^ neurons (Fig. 4h, i) that is consistent with DMH^Vglut2^ neurons driving the elevation of Cort levels during the early active period (Fig. 2).

### Ablation, inhibition, or disruption of GABAergic signaling in DMH^Vgat^ neurons reduces the daily peak in Cort secretion only under constant darkness

**Figure 7.**
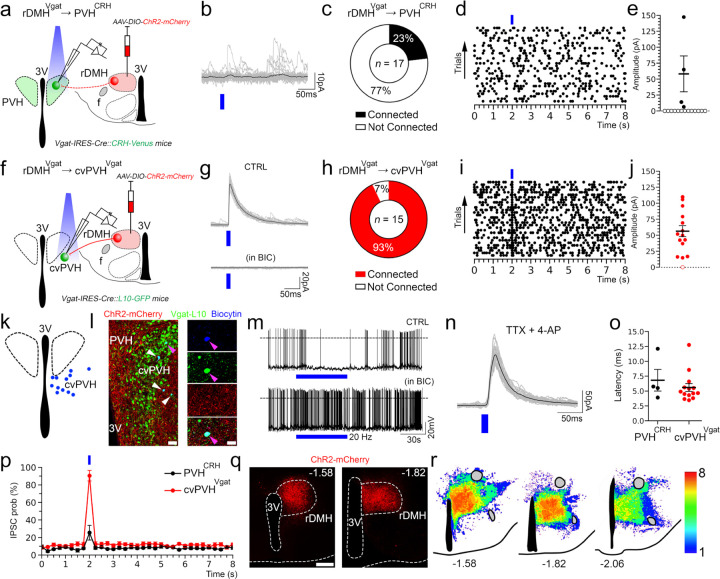
In vitro optogenetic stimulation of the GABAergic input from the rostral DMH spares PVH^CRH^ neurons and directly inhibits GABAergic neurons in the cvPVH. (**a**) A schematic representation of the recordings to test rDMH^Vgat^ → PVH^CRH^ connectivity; *Vgat-ires-Cre::CRH-Venus* mice were injected with *AAV-DIO-ChR2-mCherry* in the rDMH (*n*=3 mice). Recordings were conducted in brain slices from ipsilateral Venus-labeled PVH^CRH^ neurons while photostimulating the ipsilateral DMH^Vgat^ input (DMH is shown on the opposite side for ease of illustration). (**b**) Stimulation of the rDMH^Vgat^ input produced no synaptic responses time-locked to the light pulses in most of the PVH^CRH^ neurons. (**c**) Percentages of PVH^CRH^ neurons responding (Connected) and not responding (Not Connected) to photostimulation of the rDMH^Vgat^ input (*n*=17 PVH^CRH^ recorded neurons from 3 mice). (**d**) Raster plot of IPSCs in a representative PVH^CRH^ neuron with photostimulation of the rDMH^Vgat^ → PVH^CRH^ input (bin duration: 50ms) showing lack of input. (**e**) oIPSC amplitude following photostimulation of the rDMH^Vgat^ → PVH^CRH^ input (filled markers, cells responding to photostimulation, *n*=4 neurons; open markers, cells not responding to photostimulation, *n*=13; mean and ± SEM of responding neurons from 3 mice). (**f**) To explore rDMH^Vgat^ → cvPVH^Vgat^ connectivity, *Vgat-ires-Cre::L10-GFP* mice (*n*=5 mice) were injected with *AAV-DIO-ChR2-mCherry* in the rDMH, and recordings were conducted in ipsilateral GFP-labeled cvPVH^Vgat^ neurons while photostimulating the rDMH^Vgat^ input. (**g**) Photostimulation of the DMH^Vgat^ input produced opto-evoked inhibitory postsynaptic currents (oIPSCs) in most of the cvPVH^Vgat^ neurons. GABAA-mediated oIPSCs recorded in cvPVH^Vgat^ neurons (*upper trace)* and blocked by bicuculline (BIC 20µM; *n*=4 neurons from 4 mice). (**h**) Percentages of cvPVH^Vgat^ neurons responding (Connected) and not responding (Not Connected) to photostimulation of the rDMH^Vgat^ input (*n*=15 cvPVH^Vgat^ recorded neurons from 5 mice). (**i**) Raster plot of IPSCs in a representative cvPVH^Vgat^ neuron with photostimulation of the rDMH^Vgat^ → cvPVH^Vgat^ input (bin duration: 50ms). (**j**) oIPSC amplitude following photostimulation of the rDMH^Vgat^ → cvPVH^Vgat^ input (filled markers, cells responding to photostimulation, *n*=14 neurons; open markers, cells not responding to photostimulation, *n*=1; mean and ± SEM of responding neurons from 5 mice). (**k**) A schematic map of 12 cvPVH^Vgat^ neurons (*n*=5 mice) that were recorded and found to receive input from DMH^Vgat^ neurons. (**l**) A photomicrograph showing four recorded GABAergic cvPVH neurons that responded to photostimulation of DMH^Vgat^ input (filled with biocytin from the recording pipette, indicated by arrowheads). DMH^Vgat^ fibers expressing ChR2-mCherry (in red) surrounded the cvPVH^Vgat^ neurons expressing GFP (in green). The neuron indicated by the magenta arrowhead is shown at higher magnification at the right, showing labeling, from top to bottom, for biocytin, GFP, mCherry, and merged. (**m**) Photostimulation trains (20Hz, train frequency; 60s, train duration and 10ms, pulse duration) inhibited the activity of the cvPVH^Vgat^ neurons (*n*=6 neurons from 2 mice; *top*) and this effect was blocked by bicuculline (20µM; *n*=2 from 2 mice; *bottom*). (**n**) oIPSCs in cvPVH^Vgat^ neurons recorded in the presence of TTX 1µM + 4-AP 200µM (*n*=6 neurons from 1 mouse) indicating monosynaptic connectivity. (**o**) oIPSC latency recorded in PVH^CRH^ (black; *n*=4 neurons from 3 mice) and cvPVH^Vgat^ neurons (red; *n*=14 neurons from 5 mice) following photostimulation of the DMH^Vgat^ input. (**p**) oIPSC probability in PVH^CRH^ (black; *n*=4 neurons from 3 mice) and cvPVH^Vgat^ neurons (red; *n*=14 neurons from 5 mice) following photostimulation of the DMH^Vgat^ input. (**q-r**) Photomicrograph of a representative rDMH injection with *AAV-DIO-ChR2-mCherry* (native signal) and density plots of injections in the rDMH (*n*= 8 mice, including experiments in a-e and f-n; illustrated on the left side of each section) following which only 23% of PVH^CRH^ neurons showed oIPSCs. Reference scale bar: in l (*left*) = 50 µm and (*right*) = 20 µm; in q = 250 µm. Atlas levels correspond to Paxinos & Franklin Atlas ^47^.

Although inputs from DMH^Vglut2^ neurons to PVH^CRH^ cells are necessary to drive the daily Cort increase, their ablation does not completely eliminate the circadian rhythm of Cort secretion as is seen with non-specific DMH ablation^13^. It has been estimated that half of the synapses to the PVH^CRH^ neurons are GABAergic^21^, and our rabies virus experiment showed that about half of the DMH cells innervating the PVH^CRH^ neurons are GABAergic. Thus, we decided to evaluate whether the DMH^Vgat^ neurons could play a complementary role in the circadian release of Cort. We hypothesized that the DMH^Vgat^ neurons could contribute to the circadian rhythms of Cort secretion either by inhibiting PVH^CRH^ neurons during the inactive cycle to suppress Cort secretion, or by dis-inhibiting PVH^CRH^ neurons (i.e., inhibiting inhibitory inputs to them) during the active cycle. Thus, we used *Vgat-ires-Cre* mice crossed with a Cre-dependent GFP reporter mouse line *R26-loxSTOPlox-L10-GFP* to generate *Vgat-ires-Cre::R26-loxSTOPlox-L10-GFP* mice (*Vgat-ires-Cre::L10*), in which Vgat neurons expressing Cre-recombinase show green fluorescence. We collect blood samples at 4 temporal points in LD (ZT 1, 7, 13, 19), and then in the same temporal points in DD (CT 1, 7, 13, 19), as we did with the *Vglut2-ires-Cre* mice. Also, we recorded LMA and Tb for 12 days in LD, and then12 days in DD. Then, we injected the DMH of *Vgat-ires-Cre::L10* mice with an *AAV10-hSyn-mCherry-DIO-DTA* to ablate the DMH^Vgat^ neurons. Four weeks after the injections, we again recorded and took blood samples from the mice in LD and DD conditions. From a total of 13 *Vgat-ires-Cre::L10* mice injected, we analyzed data from 8 mice in which a blinded investigator found that the injection sites covered at least 70% of the DMH bilaterally (Fig. 5a-b, Ext. Fig. 7a-b). DMH^Vgat^ ablation did not alter the daily rhythm of Cort release in LD but reduced the CT13 peak under DD from 26.5 ± 2.9 ng/ml in Ctrl to 14.1 ±4.4 ng/ml (p=0.014, Fig. 5c-d). Similarly, there was no statistically significant change in the CI of Cort in LD, but it was reduced by 72.4 ±21.3% in DD after ablation of DMH^Vgat^ neurons (p=0.01, Fig. 5e). Importantly, ablation of DMH^Vgat^ neurons did not change the levels of Cort at its nadir (ZT1 or CT1), which might have been expected if DMH^Vgat^ neurons with direct inputs to PVH^CRH^ cells contributed to circadian rhythms of Cort by suppressing secretion during the daily nadir. To test the effect of ablation of DMH^Vgat^ neurons on other aspects of Cort secretion, at the end of the experiment we also measured Cort levels after one hour of restraint stress, but these were unaffected by the ablations (Ext. Fig. 7c). In contrast to the effects on Cort rhythms, LMA was dramatically reduced during the dark phase of LD after DMH^Vgat^ ablation, the total count over the dark period decreasing from 873.5 ± 45 counts to 429.5 ± 52.5 counts (p<0.001); LMA was reduced even further in DD during the presumptive dark phase from 771.2 ± 40.6 counts to 401.7 ± 44.9 counts (p<0.001, Ext. Fig. 7h), resulting in a reduction in the Circadian Index of LMA by 38.23 ±9.2% in LD (p=0.005) and by 50.61 ±12.9% in DD (p=0.003, Ext. Fig. 7i-j), and a significant reduction in the amplitude of the peak at 24 hrs in the periodogram and in the cosinor analysis of both LD and DD (LD: p<0.001; DD: p<0.001, Ext. Fig. 7e and g). Thus, most of the reduction in LMA after DMH lesions appears to be due to loss of DMH^Vgat^ neurons. In the mice with ablation of DMH^Vgat^ neurons, Tb was about 0.3°C lower throughout the day in LD and during the subjective dark period in DD, but there was no statistically significant change in the CI or cosinor analysis (Ext. Fig. 7l-s).

**Figure 8.**
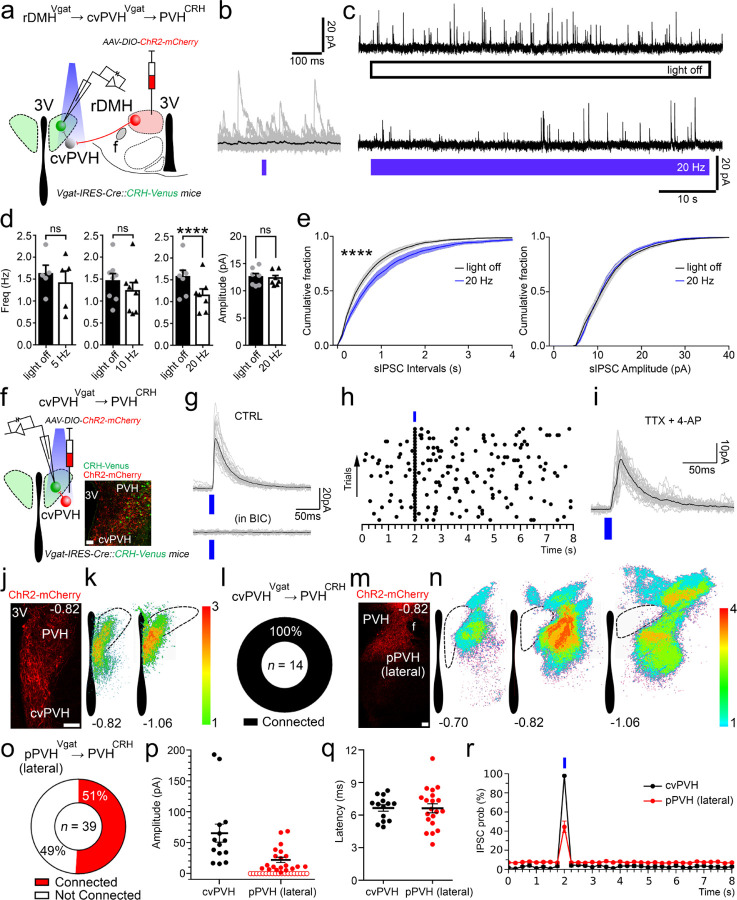
GABAergic neurons in the rostral DMH disinhibit the PVH^CRH^ neurons via GABAergic neurons in the cvPVH. (**a**) To test the rDMH^Vgat^ → cvPVH^Vgat^ → PVH^CRH^ circuit, we injected *AAV-DIO-ChR2-mCherry* into the rDMH of *Vgat-ires-Cre::CRH-Venus* mice (*n*=3 mice). Recordings were conducted in ipsilateral PVH^CRH^ neurons while photostimulating rDMH^Vgat^ synaptic terminals (DMH is shown on the opposite side of the brain for ease of illustration). We tested the effects of single light pulses (10ms) and trains of optostimulation (10ms individual pulse duration; 5, 10, and 20Hz stimulation frequency; 60s train duration) on spontaneous IPSC (sIPSC) frequency in PVH^CRH^ neurons. (**b**) Single light pulses did not produce oIPSCs in most PVH^CRH^ neurons, but (**c**) trains of stimulation reduced the sIPSCs (*C; top* trace: light off; *bottom* trace: *light* on). (**d**) Change in sIPSC frequency following trains of photostimulation at 5, 10 and 20 Hz compared to sham stimulation (light off). Photostimulation at 5 and 10 Hz showed a trend toward reduction in sIPSC frequency that did not reach statistical significance (at 5 Hz: *n*=5 neurons, *paired t-test, p*=0.2772; at 10 Hz: *n*=8 neurons, *paired t-test, p*=0.0545; from 3 mice) whereas photostimulation at 20 Hz significantly reduced sIPSCs frequency (*n*=7 neurons from 3 mice, paired t-test, ****p<0.0001), without affecting their amplitude (*n*=7 neurons from 3 mice; *paired t-test, p*= 0.7593). (**e**) Mean cumulative distribution plots of the sIPSC inter- event intervals show that intervals between sIPSCs were longer (*left*; 0.1s bins; Kolmogorov-Smirnov test, ****p<0.0001; 20Hz vs light off) but that there was no change in sIPSC amplitude (*right*; 1pA bins; *Kolmogorov-Smirnov test, p=*0.1414; 20Hz vs light off) as compiled from 7 PVH^CRH^ neurons from 3 mice (blue: 20Hz; black: light off; shaded areas: ± SEM). (**f**) To test the cvPVH^Vgat^ → PVH^CRH^ input, we injected *AAV-DIO-ChR2- mCherry* into the cvPVH of *Vgat-ires-Cre::CRH-Venus* mice (*n*=3 mice). Recordings were conducted from ipsilateral PVH^CRH^ neurons while photostimulating cvPVH^Vgat^ neurons and terminals. (**g**) Stimulation of the cvPVH^Vgat^ input evoked GABAA-mediated oIPSCs in PVH^CRH^ neurons (Bicuculline, 20µM; *n*=4 neurons from 3 mice). (**h**) Raster plot of IPSCs in a representative PVH^CRH^ neuron showed tight correlation with photostimulation of the cvPVH^Vgat^ → PVH^CRH^ input (bin duration: 50ms). (**i**) TTX -resistant oIPSCs in cvPVH^CRH^ neurons (*n*=6 neurons from 2 mice; TTX 1µM + 4-AP 250µM)) indicating monosynaptic connectivity of the cvPVH^Vgat^ → PVH^CRH^ input. (**j-k**) A representative photomicrograph and density plots of injections of AAV-ChR2-mCherry in the cvPVH (*k*; *n*=3 mice) and (**l**) percentages of PVH^CRH^ neurons responding to photostimulation of the cvPVH^Vgat^ input (Connected; *n*=14 PVH^CRH^ recorded neurons from 3 mice). (**m-n**) Representative injection site and density plots of injections of AAV-ChR2-mCherry along the lateral margin of the PVH (pPVH) (*n*=4 mice) and (**o**) percentages of PVH^CRH^ neurons responding (Connected) and not responding (Not Connected) to photostimulation of the GABAergic input from the lateral pPVH (*n*=39 neurons from 4 mice). (**p**) Amplitude (filled markers, cells responding to photostimulation, open markers, cells not responding to photostimulation; mean and ± SEM of responding neurons) and (**q**) latency of oIPSCs in PVH^CRH^ neurons evoked by photostimulation of the cvPVH (black; *n*=14 neurons from 3 mice) and lateral PVH (red; *n*=39 neurons from 4 mice, mean and ± SEM). (**r**) oIPSC probability in PVH^CRH^ neurons following photostimulation of the input from the cvPVH^Vgat^ (black; *n*=14 neurons from 4 mice) and lateral pPVH^Vgat^ neurons (red; *n*=39 neurons 4 mice). In all experiments we visualize only the native signal. Reference scale bar: in G = 50 µm; K and N = 100 µm. f, fornix, 3V, third ventricle. Atlas levels are from Paxinos & Franklin Atlas ^47^.****, *p* < 0.0001.

We interpreted these results as indicating that it was unlikely that DMH^Vgat^ neurons played a role in suppressing Cort secretion during the inactive (light) phase, but that they potentially accounted for the other half (not caused by the DMH^Vglut2^ neurons) of the surge in Cort levels during the active (dark) phase. We next evaluated whether this effect was due to GABA release from these neurons (vs. some other neurotransmitter in the same neurons). Thus, we placed *AAV8-SYN-EGFP-iCre* or *AAV8-DIO-GFP* injections in the DMH in *Vgat*^*loxP/loxP*^ (*Vgat-flox*) mice to delete expression of functional Vgat protein and so to abolish GABA transmission from the GABAergic neurons of the DMH. From 12 mice injected with AAV-EGFP-iCre in the DMH, 7 with bilateral injections covering at least 70% of the DMH were included in the analysis (Fig. 5f-g, Ext. Fig. 8a). *Vgat* deletion from DMH neurons did not change the circadian regulation of Cort release in LD, but again reduced the CI of Cort release in DD by 76.6 ±13.6% (p=0.008, Fig. 5j), by reducing the CT13 peak of Cort levels in DD from 27.0 ± 4.5 ng/ml in controls to 11.7 ± 2.1 ng/ml in *Vgat-*deleted mice (p=0.006, Fig. 5h). Thus, most if not all of the effect of the DMH^Vgat^ neurons on the circadian release of Cort is mediated by GABA. Interestingly, although the total levels of LMA during the dark period were decreased by Vgat deletion in the DMH (from 979.2 ± 66.8 counts in the control group to 681.5 ± 55.6 counts in the deletion group during LD; p<0.001, Ext. Fig. 8f), the reduction was not nearly as profound as seen after the ablation of the DMH^Vgat^ neurons, suggesting that other transmitters released from those neurons could play a role in regulating LMA. Results in DD were similar (from 979.2 ± 66.8 total counts in the DMH^Vgat/flox^-Control group to 681.5 ± 55.6 counts in the DMH^Vgat/flox^-EGFP-iCre mice during DD; p<0.001, Ext. Fig. 8f), so that the CI of LMA was reduced by only 25.7 ± 6% in LD (p=0.001) and 33.3 ± 12.6% in DD (p=0.021, Ext. Fig. 8g), similar to the effect observed in the amplitude in the cosinor analysis (Ext. Fig. 8g-h). Tb of DMH^Vgat^– deleted mice was also reduced during the dark phase by 0.2 °C in LD (mostly between ZT14-18; p=0.001, Ext. Fig. 8j-n), and in the subjective dark by 0.3°C in DD (p<0.001, Ext. Fig. 8l-n). As a result, the Circadian Index of Tb was reduced in the DMH^Vgat^–deleted mice by 20.8 ± 8.8% in LD (p=0.037) and by 26.6 ± 8.9% in DD (p=0.012, Ext. Fig. 8o), similar to the reduction in the amplitude in the cosinor analysis (Ext. Fig. 8p).

To determine whether these chronic conditional lesions or impairments of GABA transmission might be affected by compensatory mechanisms, we examined the effect of acute inhibition of the DMH^Vgat^ neurons on the circadian release of Cort. We placed injections of *AAV-DIO-hGlyR-mCherry* in the DMH of *Vgat-ires-Cre* mice. From 7 mice injected, 5 mice with bilateral injection covering at least 70% of the DMH were included for analysis (Fig. 5k-l, Ext. Fig. 9a). After 4 weeks allowing the full expression of hGlyR, mice were injected with either vehicle or IVM (5mg/Kg, ip) at ZT1 in LD, or CT1 in DD, and Cort levels were sampled 24h and 36h later. The inhibition of the DMH^Vgat^ neurons did not change the Cort levels under LD, but reduced the level at CT13 from 22.8 ± 6.1 ng/ml after vehicle to 6.9 ± 1.1 ng/ml after IVM administration (p=0.006, Fig. 5m) and reduced the CI by 95.7 ±6.9% in DD (p=0.006, Fig. 5n). Similar to the effects of their ablation, the inhibition of DMH^Vgat^ neurons reduced LMA during the dark or subjective dark period between 24–48 h after IVM administration, reducing the CI in LD by 77.7 ± 5.7% (VEH p=0.001, Ext. Fig. 9b-d), and in DD by 80.7 ± 5% (p=0.002, Ext. Fig. 9e-g). The Tb during the dark period or presumptive dark period was lower after IVM administration when compare vs VEH (which includes the effect of the handling in contrast to the baseline), therefore, the CI of Tb was significantly reduced in both LD and DD, 24–48h after IVM administration by 77.7 ± 8.1% in LD (p=0.005, Ext. Fig. 9h-j), and by 68.3 ± 14% in DD (p=0.004, Ext. Fig. 9k-m). In all three experiments (ablation, deletion, and inhibition), the changes observed in levels or rhythms of LMA or Tb were not observed in the anatomical controls (animals with injections that missed the DMH). These data suggest that DMH^Vgat^ neurons participate in the circadian peak of Cort release at the beginning of the active period (as seen in DD), likely reducing the inhibitory tone to the PVH^CRH^ neurons at this temporal point, but that in LD light can act as a cue for elevation of Cort at the onset of the active period, presumably through another pathway. The ablation of DMH^Vgat^ neurons did not, however, increase the levels of Cort secretion at ZT1 or CT1, indicating that they do not shape circadian secretion of Cort by suppressing levels at their nadir. Also, the DMH^Vgat^ neurons play a larger role than DMH^Vglut2^ neurons in the circadian regulation of LMA, as the ablation of DMH^Vgat^ but not the DMH^Vglut2^ neurons dramatically reduced LMA during the dark or subjective dark periods.

### The activation of DMH GABAergic neurons increases Cort levels, whereas cvPVH GABAergic neurons constrain Cort levels

As the ablation of the DMH^Vgat^ neurons prevented the peak in DD conditions, we tested whether the activation of those neurons can induce Cort release. We placed injections of *AAV8-DIO-hM3Dq-mCherry* in the DMH of *Vgat-ires-Cre* mice. From 8 mice injected, 6 mice were confirmed to have injection sites covering at least 70% of the DMH bilaterally (Fig. 6a-b). CNO administration at the beginning of the light phase (ZT2) elevated the Cort levels to 36.8 ± 9 ng/ml one hour after administration, while Cort was only 10.2 ± 2 ng/ml after vehicle (p=0.016, Fig. 6c). Although activation of DMH^Vglut2^ neurons produced a larger increase in Cort levels (>150 ng/ml) than activation of DMH^Vgat^ neurons, in both cases the increase in Cort levels caused by CNO was at least as great as the circadian peak of Cort in undisturbed littermates. Thus, activation of either DMH^Vgat^ or DMH^Vglut2^ neurons is capable of elevating Cort levels within the range achieved by circadian rhythms.

Because activation of the DMH^Vgat^ neurons is expected to release GABA and inhibit their postsynaptic targets, we hypothesized that the DMH^Vgat^ neurons induce Cort release by disinhibiting the PVH^CRH^ neurons through inhibitory relay neurons, similar to the mechanism by which GABAergic inputs from the arcuate nucleus increase Cort secretion during food deprivation ^19^. To understand the circuit through which DMH GABAergic neurons promote CRH secretion, we crossed *Vgat-ires-Cre* mice with *CRH-Venus* reporter mice to generate *Vgat-ires-Cre::CRH-Venus* mice. We then injected the DMH of 4 *Vgat-ires-Cre::CRH-Venus* mice with *AAV8-DIO-ChR2-mCherry*. These injections were relatively small (3–9 nl compared to 24–45 nl for the other experiments) and covering mostly the rostral part of the DMH (rDMH). We found that axons and terminals labeled anterogradely (expressing mCherry, in red) from rDMH^Vgat^ neurons largely avoided PVH^CRH^ neurons (green). The bulk of the labeled terminal field was in areas surrounding the PVH, as well as in the region medial and ventral to the PVH^CRH^ neurons and extending into the caudal ventral PVH (Fig. 6d-e, 6j-k). Although the PVH contains very few GABAergic cells, many are found in the region surrounding the PVH, known as the peri-PVH area, as well as in the adjacent caudal ventral part of the PVH. As terminals from the DMH^Vgat^ neurons blanket the caudal ventral PVH as well as the adjacent peri-PVH just outside it, for the sake of simplicity we will refer to this region as the cvPVH. Local inhibitory inputs within the PVH have been described from this region^22–24^, so we decided to explore whether the cvPVH^Vgat^ neurons might mediate disinhibition of the PVH^CRH^ neurons during the circadian peak of Cort. To explore whether the cvPVH^Vgat^ neurons might make monosynaptic contacts with PVH^CRH^ neurons, we combined conditional retrograde rabies tracing from PVH^CRH^ neurons with in situ hybridization for Vgat mRNA using the CRH-ires-Cre mice (n=2). We observed doubly labeled neurons in the cvPVH suggesting monosynaptic GABAergic inputs from this region to the PVH^CRH^ neurons (Fig. 6f-g). We next injected 3 *Vgat-ires-Cre::CRH-Venus* mice with *AAV8-DIO-ChR2-mCherry* in this part of the PVH. The injections covered the ventral and lateral region of the PVH including the cvPVH, and labeled axons ramified inside the PVH, including ones making appositions with the CRH-Venus neurons (Fig. 6h-i). We then investigated whether the DMH^Vgat^ neurons innervate the cvPVH^Vgat^ neurons. Following injections of *AAV8-DIO-ChR2-mCherry* into the rostral DMH of *Vgat-ires-Cre::L10-GFP* mice (*n*=7) we found dense terminal labelling in apposition to the cvPVH^Vgat^ neurons, suggesting direct connectivity (Fig. 6j-k). These neuroanatomical findings support the hypothesis of a circuit where the DMH^Vgat^ neurons disinhibit PVH^CRH^ neurons through direct inhibition of the cvPVH^Vgat^ interneurons (DMH^Vgat^ → cvPVH^Vgat^ → PVH^CRH^).

To determine whether the cvPVH^Vgat^ neurons play a role in the circadian release of Cort, we ablated the Vgat expressing neurons in the cvPVH by placing injections of *AAV10-hSyn-mCherry-DIO-DTA* in the cvPVH of *Vgat-ires-Cre* mice (*n*=5). In all cases, the injection sites marked by mCherry expression involved the cvPVH, where Vgat cells projecting to the PVH^CRH^ neurons are found, and the body of the PVH, where few Vgat cells are seen. (Fig. 6l-m, Ext. Fig. 10a). After four weeks to allow full expression of DTA, we recorded LMA and Tb in the mice for 12 days in both LD and DD, and we collected blood samples for Cort measurements toward the end of both the LD and DD periods. The cvPVH^Vgat^ ablated mice showed higher levels of Cort but only at the beginning of the active phase, from 21.3 ± 2.3 ng/ml in controls to 59.8 ± 11.3 ng/ml in cvPVH^Vgat^ ablated mice in LD (p<0.001, Fig. 6n), and from 25.6 ± 5.4 ng/ml to 51.3 ± 8.7 ng/ml in DD (p<0.001, Fig. 6o). The CI of Cort also increased by 180.2 ± 52.9% in LD (p=0.01) and 100.4 ± 33.9% in DD (p=0.036, Fig. 6p). At the end of the protocol, we also exposed the mice to restraint stress and found that the Cort levels were significantly higher in the cvPVH^Vgat^ ablated mice (p=0.047, Ext Fig. 10b). No major changes were observed in LMA or Tb with ablation of cvPVH^Vgat^ neurons (Ext. Fig. 10c-r). These data indicate that the cvPVH^Vgat^ neurons play an important role in inhibiting Cort secretion, even limiting its peak during the daily surge in Cort secretion at CT13 and in response to restraint stress. However, these neurons do not appear to play a role in suppressing Cort levels at their daily nadir. DMH^Vgat^ inhibition of cvPVH^Vgat^ neurons partially disinhibits the PVH^CRH^ neurons, allowing the surge in Cort secretion at CT13, but completely eliminating the cvPVH^Vgat^ neurons allows even greater stimulation of the PVH^CRH^ cells at that time by DMH^Vglut2^ inputs.

### Dissecting the synaptic inputs of the DMH^Vgat^→ cvPVH^Vgat^→ PVH^CRH^ circuit

To dissect the synaptic mechanisms by which the DMH^Vgat^ neurons control the PVH^CRH^ neurons, we injected the DMH of a new cohort of seven *Vgat-ires-Cre::CRH-Venus* mice with *AAV8-DIO-ChR2-mCherry*. We then performed patch clamp recordings in brain slices from green-labelled PVH^CRH^ neurons on the same side of the brain while photoactivating the red DMH^Vgat^ terminals in the PVH (Fig. 7a). Our rabies virus tracing data indicated that PVH^CRH^ neurons receive a direct GABAergic input from the DMH but mainly from its caudal portion (Ext. Fig. 6e-f). In support of these results, we observed that in three mice where the injection site was restricted mostly to the rostral part of the DMH (rDMH), the photostimulation of the DMH^Vgat^ input evoked inhibitory synaptic responses in only 4 out of 17 recorded PVH^CRH^ neurons (23%, *n*=17 ;Fig. 7a-e, r), whereas in four mice in which ChR2 expression involved the more caudal portion of the DMH, the photostimulation of the DMH^Vgat^ input evoked oIPSCs in nearly all PVH^CRH^ neurons (96%, *n*=23; Ext. Fig. 11a-g). As the direct GABAergic inputs from the caudal DMH to PVH^CRH^ neurons do not appear to play a role in inhibiting the PVH^CRH^ neurons at the circadian nadir of Cort, we focused on whether the rDMH GABAergic inputs could disinhibit the PVH^CRH^ neurons at the daily peak of Cort secretion.

To investigate whether rDMH^Vgat^ neurons directly inhibit the cvPVH^Vgat^ neurons, we expressed *AAV8-DIO-ChR2-mCherry* in the rDMH of 5 *Vgat-ires-Cre::L10-GFP mice*. We then recorded from GFP-labelled cvPVH^Vgat^ neurons while stimulating the ipsilateral putative rDMH^Vgat^ input to them (rDMH^Vgat^ →cvPVH^Vgat^) (Fig. 7f). Photostimulation evoked opto-inhibitory postsynaptic currents (oIPSCs) in 14 out of 15 recorded cvPVH^Vgat^ neurons. These oIPSCs depended on GABAA receptor-mediated signaling, as they were blocked by bicuculline (*n*=4; Fig. 7g-m). Furthermore, oIPSCs persisted in the presence of TTX (1µM) and 4-AP (200 µM) indicating monosynaptic connectivity (*n*=6, Fig. 7n).

When we activated the rDMH^Vgat^ terminals at different frequencies while recording from the PVH^CRH^ neurons neurons in which single light pulses did not evoke oIPCS, we found that photostimulation of the ipsilateral rDMH^Vgat^ input at 5 or 10 Hz caused a reduction in the number of spontaneous IPSCs, which became statistically significant at 20 Hz (*n*=7; Fig. 8a-e). These results indicate that the rDMH^Vgat^ neurons can disinhibit PVH^CRH^ neurons by reducing their GABAergic afferent input.

Our conditional tracing study and CRACM recordings suggest that the rDMH^Vgat^ input disinhibits the PVH^CRH^ neurons at least in part through the GABAergic neurons located in the cvPVH. We therefore tested whether the cvPVH^Vgat^ neurons can directly inhibit the PVH^CRH^ neurons (cvPVH^Vgat^ → PVH^CRH^). We placed *AAV8-DIO-ChR2-mCherry* injections in the cvPVH of 3 *Vgat-ires-Cre::CRH-Venus mice*. We then recorded from labelled PVH^CRH^ neurons while photostimulating cvPVH^Vgat^ neurons and terminals. Photostimulation of cvPVH^Vgat^ neurons evoked oIPSCs in all PVH^CRH^ neurons recorded (*n*=14). These oIPSCs were mediated by GABAA receptor signaling as they were blocked by bicuculline (*n*=4) and were resistant to TTX+4-AP, indicating monosynaptic connectivity (*n*=6) (Fig. 8f-l). Interestingly, in four cases where the ChR2 expression was more dorsal and lateral involving the portion of the pPVH just lateral to the PVH only about half of the PVH^CRH^ neurons we recorded from showed oIPSCs (*n*=4) (Fig. 8m-r). Taken together, these results indicate that rostral DMH^Vgat^ (rDMH^Vgat^) neurons can disinhibit PVH^CRH^ neurons through GABAergic interneurons located in the cvPVH (rDMH^Vgat^ → cvPVH^Vgat^ → PVH^CRH^), promoting the circadian increase of Cort levels at CT13.

## Discussion

This study identifies a circuit from the SCN with two obligate relays, in the SPZ and DMH, for controlling the circadian rhythm of Cort secretion. Nearly all SPZ neurons express Vgat (are GABAergic), and the ablation of SPZ^Vgat^ neurons eliminates the circadian rhythms of Cort secretion. However, deleting the *Vgat* gene from the SPZ has little effect on the Cort rhythm, suggesting that the message is conveyed from the SPZ to the DMH by another co-transmitter. Ablation, deletion or inhibition of either DMH^Vglut2^ or DMH^Vgat^ neurons diminishes the circadian rhythm of Cort secretion, and the input from the DMH to the PVH^CRH^ neurons takes the form of two parallel circuits with direct input from DMH^Vglut2^ neurons exciting PVH^CRH^ cells (the DMH^Vglut2^→ PVH^CRH^ pathway). By contrast, rDMH^Vgat^ neurons disinhibit PVH^CRH^ cells by rDMH^Vgat^ neurons inhibiting intermediary cvPVH^Vgat^ neurons (the rDMH^Vgat^ → cvPVH^Vgat^ → PVH^CRH^ pathway). These circuits participate in the circadian rhythms of Cort secretion in a complementary way. The direct input from the DMH^Vglut2^ neurons to the PVH^CRH^ cells appears to be necessary to maintain the daily peak in Cort under both LD and DD whereas the rDMH^Vgat^ disinhibitory circuitry appears to be necessary for the daily increase in Cort levels at CT13 only during constant dark conditions. This suggests that the rDMH^Vgat^ neurons mediate the disinhibition of PVH^CRH^ neurons in anticipation of the circadian active period, but that under LD conditions the daily light signal itself can activate circuitry to disinhibit PVH^CRH^ neurons at ZT13 even in the absence of the DMH^Vgat^ circadian input. This relationship is similar to the “masking” phenomenon by which there is a reduction in LMA during the light phase of LD, even in mice in which the SCN has been ablated and which have no circadian rhythm of LMA in DD^25–27^. The origin of this light-induced signal for elevation of Cort at the beginning of the active period is not known, but apparently does not involve the DMH^Vgat^ neurons.

This finding points out a *limitation in our study*, that although we specifically focused on the role of DMH neurons in the circadian regulation of Cort, DMH neurons may also participate in other Cort responses. For example, the DMH participates in stress responses, including the elevation of ACTH and Cort during psychosocial stress ^14,15,28–30^. Our work was designed to minimize stress during our circadian sampling by taking all blood samples within 1 minute of touching the animal, so that we could isolate circuitry that contributes to the circadian regulation of Cort secretion in anticipation of the active period. As a control for whether this circadian circuitry also mediated stress-induced Cort responses, we also examined the Cort response to acute restraint in the same animals, and found that ablating either the glutamatergic or GABAergic neurons in the DMH had little if any effect on restraint stress-induced Cort levels. In contrast, the mice with ablation of cvPVH^Vgat^ neurons showed an exacerbated Cort response to restraint, suggesting that the cvPVH^Vgat^ neurons may tonically inhibit PVH^CRH^ cells, even during restraint stress.

A second limitation in our study was that our ablations and deletions of SPZ^Vgat^ neurons encroached upon the dorsal edge of the SCN, which also is composed of GABAergic neurons. Previous studies of SCN ablation have shown that extensive bilateral loss of SCN neurons (typically >75%) is required to cause loss of circadian rhythmicity^31^. In our ablation experiments, no more than 20% of the most dorsal extent of the SCN on one side of the brain was involved, so that encroachment on the SCN was unlikely to cause the profound loss of Cort, LMA, and Tb rhythms we observed. A recent study using SCN^Vgat^ deletion methods similar to this study found that unilateral or partial Vgat deletion in the SCN had little effect on LMA or Tb rhythms^32^. Thus, involvement of the dorsal margin of the SCN in our SPZ^Vgat^ ablation and deletion studies was unlikely to affect those results.

Another limitation of our study was that while we examined the sources of circadian input from the DMH to the PVH^CRH^ neurons, our output measure was serum Cort levels. While CRH secretion is the dominant influence on secretion of ACTH by the pituitary gland, other hormones, such as arginine vasopressin may be secreted into the hypophysial portal circulation and can contribute to ACTH secretion ^33^. However, *Crh−/−*mice, despite having normal circadian rhythms of LMA, lack (males) or have minimal (females) circadian increases in Cort secretion ^34^. Thus, the focus on the PVH^CRH^ neurons as the main driver of Cort rhythms is justified.

A fourth limitation is that, although the DMH^Vgat^ neurons appear to be divided into a caudal DMH^Vgat^ group that directly inhibits PVH^CRH^ cells and a rostral DMH^Vgat^ group that disinhibits PVH^CRH^ neurons (by inhibiting cvPVH GABA neurons that innervate the PVH^CRH^ cells), our stimulation, ablation, deletion, and inhibition studies probably included both groups. Because the caudal DMH^Vgat^ neurons directly inhibit PVH^CRH^ cells, any participation in circadian regulation of Cort should be by suppressing secretion, e.g., during the daily nadir. However, DMH^Vgat^ ablations or deletions had no effect on the daily nadir, and only affected the daily peak. The rostral DMH^Vgat^ neurons, by contrast, disinhibit PVH^CRH^ cells, and their ablation or deletion reduces the daily circadian peak of Cort secretion in DD. Interestingly, although the levels of Cort after chemogenetically activating the DMH^Vglut2^ neurons were in the range typically seen during stress responses (>150 ng/ml), the peak levels seen after chemogenetic activation of the DMH^Vgat^ neurons were <40 ng/ml. This lower level of Cort secretion may reflect the fact that both rDMH^Vgat^ neurons which disinhibit PVH^CRH^ neurons and caudal DMH^Vgat^ neurons that inhibit CRH cells were being activated at the same time. Single nucleus RNA sequencing studies indicate that the DMH comprises more than 20 different cell types^35^, roughly evenly divided between GABAergic and glutamatergic neurons. Further progress will require identifying the unique genetic signatures for the different classes of DMH neurons ^35^ to test their roles in both circadian and stress-induced secretion of Cort.

### Relative contribution of DMH^Vglut2^ and DMH^Vgat^ neurons to circadian rhythms

It is also important to point out that the roles of the DMH^Vgat^ and DMH^Vglut2^ neurons in the circadian regulation of Cort secretion appear to depend upon release of GABA and glutamate, respectively, because deletion of just the *Vgat* or *Vglut2* genes from neurons in the DMH had effects similar to ablating the entire neuron population. The ablation of the SPZ^Vgat^ neurons also caused almost complete loss of the circadian rhythm of Cort secretion and LMA and reduced the CI of Tb by about 50%. These effects on LMA and Tb were similar to a previous report using nonspecific SPZ ablation in rats^36^. However, the deletion of the *Vgat* gene in the SPZ had almost no effect on the circadian rhythm of Cort and only modestly reduced the CI of LMA and Tb. This disparity may have been due in part to the SPZ^Vgat^ ablations involving the ventral SPZ more extensively and in some cases encroaching on the SCN on one side of the brain. However, the contribution of SPZ^Vgat^ neurons to these circadian rhythms may rely upon other transmitters expressed by these neurons. Information about the expression of peptides or other transmitters by SPZ neurons is limited so far, and will likely depend upon single cell mRNA expression profiling. On the other hand, many DMH neurons also express peptides, including galanin, dynorphin, neuropeptide Y, CART, orexin, and many others ^37–41^. While the different patterns of gene expression by DMH neurons may help us to classify them and provide key marker genes for neurons that participate in particular roles or have specific projections, it does not appear that the DMH neurons depend upon peptidergic transmission to produce the circadian pattern of Cort secretion.

In this work, we also were able to dissect the role of the DMH^Vglut2^ vs DMH^Vgat^ neurons in the global reduction of LMA and Tb and dramatic reduction in the circadian rhythm of LMA reported after non-specific ablation of the DMH in rats^13^. The DMH^Vgat^ neurons appear to be particularly important for the circadian increase of LMA during the active period, as their ablation reduced the amount of LMA by about half across the entire dark or presumptive dark cycle. By contrast, ablation of the DMH^Vglut2^ neurons caused a much smaller decrease in LMA which was predominantly confined to the transitions between the light and dark periods. The combination would explain the low levels of LMA with almost complete loss of circadian rhythms of LMA reported in rats after non-specific DMH cell ablation^13^. The ablation of either the DMH^Vgut2^ or DMH^Vgat^ neurons reduced Tb mainly during the dark and presumptive dark phase when the animals are most active. This effect is consistent with the known increase in Tb that occurs during LMA. In addition, dorsal hypothalamic/DMH glutamatergic neurons also play a direct role in promoting thermogenesis by projections to the raphe pallidus^30,42^.

### Synergistic regulation of Cort rhythms by the DMH Glutamatergic and GABAergic populations

Previous work in rats had shown that non-specific ablation of the DMH in rats prevented the daily increase of Cort levels associated with the active cycle and reduced Cort secretion to the level normally measured during the inactive period when the animals are mainly asleep ^13^. Because the effect of the DMH lesions was to eliminate the daily peak in Cort levels without affecting basal (nadir) levels, we initially hypothesized that the circadian input from the DMH to the PVH^CRH^ neurons was likely to be excitatory, i.e., glutamatergic. In this study we tested that hypothesis and observed that the DMH^Vglut2^ neurons play an important role in the circadian elevation of Cort at CT13 but that ablation of the DMH^Vglut2^ neurons did not completely eliminate the circadian surge in Cort. We therefore tested the role of DMH^Vgat^ neurons and were surprised to find that they also participate in the circadian elevation of Cort, particularly in DD, at CT13 suggesting that the DMH^Vgat^ neurons would be likely to disinhibit PVH^CRH^ cells at this time.

These observations caused us to examine the origins of the GABAergic inputs to PVH^CRH^ neurons. The PVH contains very few GABAergic neurons within its borders, whereas the pPVH is rich in GABAergic neurons and it has been proposed that the pPVH^Vgat^ neurons could provide tonic inhibition of the PVH^CRH^ neurons and function as a “brake” upon their secretion of CRH^24^. On the other hand, other studies have found that some of the GABAergic inputs to PVH^CRH^ neurons derive from neurons that are distant from the PVH. For example, a recent study from Douglass and colleagues reported that the elevation of Cort levels during fasting depends upon AgRP neurons in the arcuate nucleus inhibiting tonically active GABAergic afferents from the bed nucleus of the stria terminalis to the PVH^CRH^ neurons, thus disinhibiting them^19^. In the case of circadian secretion of Cort, we found that the rDMH^Vgat^ neurons project to cvPVH^Vgat^ neurons, and that cvPVH^Vgat^ neurons provided a particularly rich source of inhibitory input to the PVH^CRH^ cells. Furthermore, activation of DMH^Vgat^ terminals in the PVH disinhibited PVH^CRH^ cells. Interestingly, the surge in Cort levels at CT13 was greater after the ablation of the cvPVH^Vgat^ neurons than in control animals, suggesting that the tonic activity of cvPVH^Vgat^ neurons places a brake on CRH secretion that is only partially lifted during the circadian daily peak. This interaction suggests that disinhibition of the PVH^CRH^ neurons may be a common motif in their regulation.

Although the evidence is strong for DMH^Vglut2^ neurons directly exciting PVH^CRH^ cells and DMH^Vgat^ neurons disinhibiting PVH^CRH^ cells via their inputs to the cvPVH^Vgat^ neurons, we cannot rule out other polysynaptic contributions driven by the DMH^Vglut2^ and DMH^Vgat^ neurons. In addition, both neuron types in the DMH may contribute to regulation of Cort under other physiological conditions. Exploring these alternatives will require resolving the DMH glutamatergic and GABAergic neurons into discrete genetically identified cell types and determining their connections and physiological roles.

### Origin of the circadian signal to the DMH^Vglut2^ →PVH^CRH^ and DMH^Vgat^ →cvPVH^Vgat^ →PVH^CRH^ pathways

Although the activity of the DMH^Vglut2^ and DMH^Vgat^ circadian inputs to the PVH^CRH^ neurons are temporally aligned in control of the circadian rhythm of Cort, neither is in phase with the transitions between the light and dark periods. In fact, in mice the activity of PVH^CRH^ neurons increases about 6 hr and the Cort levels rise about 3 hr prior to the onset of the active (dark or presumptive dark) cycle, and reach a peak shortly after the active cycle begins, only to fall back to low levels by halfway through the active cycle ^43,44^. Similarly, in humans the cortisol levels begin to rise about halfway through the habitual sleep cycle and are about twice as high at 8am as they are at 4pm ^45^. As indicated by our rabies virus tracing experiments (Ext. Fig. 6b-d) and previous observations by others ^4,5,44^, there are few if any direct projections from the SCN to the PVH^CRH^ neurons. It has been suggested that the sparse SCN^VIP^ inputs to regions of the PVH that are nearby the CRH neurons may influence their firing via volume transmission^44^. However, our data indicate that such inputs are insufficient to cause the daily circadian surge in Cort secretion in the absence of relays in the SPZ and DMH. The fact that the timing of the increase in Cort secretion is offset by about 6hr from the light-dark cycle suggests that there is further circuitry downstream of the SCN that converts its signal, which is tied to the light-dark cycle, into a timing signal that starts the increase in Cort several hours prior to the onset of the active period. In addition, in nocturnal (mice) and diurnal (humans) species the onset of Cort secretion bears the same time relationship to onset of the active phase, even though their relationships to the light cycle (and the peak activity of SCN neurons) are 180 degrees out of phase with each other.

Here, our data suggest that the circadian pattern of Cort secretion depends upon a multi-synaptic circuit from the SCN to the SPZ and then the DMH. The ventromedial SPZ receives a large portion of the SCN output, and projects heavily to the DMH in both rats and mice^6,12,16,46^. Most SPZ neurons have activity patterns that are in antiphase to the SCN (i.e., are more active during the dark period) and they are almost uniformly GABAergic^9,10^, but our results point to a different mechanism than GABA release in relaying the circadian rhythm of Cort secretion. The SPZ also receives inputs from a wide range of other hypothalamic cell groups. Thus, it is possible that various configurations of polysynaptic connections in the SPZ might allow it to produce timing signals that are driven by the SCN but not in phase with it. Such a multi-synaptic pathway is consistent with the smooth and gradual increase in Cort secretion that occurs prior to the active phase, allowing the SPZ^Vgat^ neurons to drive the firing of DMH^Vgat^ and DMH^Vglut2^ neurons that are responsible for the circadian rhythm of Cort secretion. Further study of the intrinsic circuitry of the SPZ and the dynamics of its interaction with the DMH will be important for understanding how the SCN signal is used to optimally time various physiological processes.

## Material and methods

### Animals.

Because adult female mice lose their circadian rhythm of Tb every 4–5 days during estrus and we needed to record circadian rhythms at specific times across many days, we used only male adult mice, age 12–16 weeks old, in these experiments. The strains of the mice were *Vgat-ires-Cre* ^48^ (JAX: 016962), *Vglut2-ires-Cre* ^48^ (JAX: 016963), *CRH-ires-Cre* ^49^ (JAX: 012704), *CRH-Venus∆Neo* ^50^, *Vglut2*^*loxP/loxP*^ (JAX:036439), *Vgat*^*loxP/loxP* 51^ (JAX: 012897) and *R26-loxSTOPlox-L10-GFP* mice ^49^. Mice were individually housed under 12–12h light/dark cycle unless the protocol specified otherwise. Room temperature were controlled in a range between 22 ± 2°C and free access to food and water was provided. All procedures were performed in accordance with the National Institutes of Health Guide for the Care and Use of Laboratory Animals, and formal approval of our protocols was obtained from the Institutionary Animal Care and Use Committee at Beth Israel Deaconess Medical Center. All precautions were taken to minimize pain and discomfort in the mice.

### Viral Vectors used.

*AAV10-hSyn-mCherry-DIO-DTA* which conditionally expresses the subunit A of diphtheria toxin in a Cre-dependent fashion and the mCherry protein in non- Cre cells (acquired from Patrick M Fuller) ^52,53^ was injected (~24nL) in *Vgat-ires-Cre or Vglut2-ires-Cre* mice. *AAV8-eSYN-EGFP-T2A-iCre* containing the genes for Cre and EGFP under the neuronal eSYN promoter (VB1089, Vector Biolabs, PA, US; 8.6X10^11^ viral genomes ml^−1^) was bilaterally injected (~24nL) in *Vglut2*^*loxP/loxP*^ and *Vgat*^*loxP/loxP*^ mice. *AAV8- Ef1a-DIO-ChR2(H134R)-mCherry* injections (UNC, addgene #AV9080, 1.4X10^13^ viral genomes ml^−1^) were used for the neuroanatomical and CRACM experiments (~3–9 nL) in *Vgat-ires-Cre or Vglut2-ires-Cre* mice. *AAV10-DIO-hGlyR-mCherry* (acquired from Patrick M Fuller) ^12,17,30^ injections were made in the DMH (~45nL) either in *Vglut2-ires-Cre* or *Vgat- ires-Cre* mice. *AAV10-EF1α-DIO-hM3Dq-mCherry* (UNC, addgene #50460 virus core; 2×10^13^ viral genomes ml^−1^) was injected (~30nL) either in *Vglut2-IRES-Cre* or *Vgat-IRES- Cre* mice. *AAV8-CAG-DIO-GFP* (~24nl) was injected as a control virus in specified experiments (UNC; addgene #59331 7X10^12^ viral genomes ml^−1^). ~45nL of *AAV8-Ef1a- DIO-TVA-mCherry* (UNC Vector Core; 1.13X10^12^ viral genomes ml^−1^) mixed 1:1 with *AAV8-CAG-DIO-rabiesG* (Stanford Vector Core; 3.4X10^12^ viral genomes ml^−1^) was injected unilaterally, and 21 days later, ~55nL of *EnvA-ΔG-rabies-EGFP* (Salk Viral Vector Core; 1.51X10^8^ viral genomes ml^−1^) were administrated in the PVH of *CRH-ires-Cre* mice. All the experiments and recordings started four weeks after the injections to allow a complete transfection of the AAVs.

### Surgeries.

Mice were deeply anesthetized with a mixture of ketamine/xylazine (100/10 mg/kg, i.p.). Radiotelemetry sensors (TA-F10, DSI, US) for body temperature (Tb) and locomotor activity (LMA) were implanted in the peritoneal cavity with a small abdominal incision. The coordinates for the stereotactic microinjections of viral vectors in the brain, from bregma were: DMH, AP= −1.75mm, ML= ±0.25mm, DV= −4.9mm; PVH, AP= −0.8mm, ML= +0.25mm, DV= −4.8mm; cvPVH, AP= −0.8mm, ML= +0.25mm, DV= −4.85mm ^47^. Mice receive analgesic treatment with meloxicam or buprenorphine post-surgery. All procedures were performed under aseptic conditions.

### LMA and Tb continuous recordings.

Average Tb and LMA were recorded every 5 min during all the protocols using the radiotelemetry DSI system. The signal from the telemetry probes, previously implanted, was received and converted with the PhysioTel HD and PhysioTel (DSI) hardware. The LMA and Tb data were analyzed using the software ClockLab Analysis version 6 (Acticmetrics).

### Plasma sampling and corticosterone immunoassay.

Blood samples (~20µl) were obtained in a microvette tube (CB300, Sarstedt, US) after a small incision in the tail in less than 1 min after onset of handling the mouse, centrifuged at 10,000 rpm for 10 min, and the plasma was collected and stored at −40°C. Samples were collected with a minimum interval of 30 hours between samples. Under the DD protocol, the first sample was obtained on the third day after the lights turned off at CT13 and then every 30 hours until we completed the 4 time points. An ELISA assay for corticosterone was performed as per the manufacturer instructions (ADI-901-097, Enzo life science, US) and the corticosterone signal read with a 405nm filter (iMark, Bio-Rad, US). Each sample was read in duplicates.

### Restraint stress protocol.

At the end of the LMA and Tb protocols, mice were physically restrained for 1 hour starting at ZT3 in a plastic tube with small holes to allow normal respiration. Blood samples were obtained at the end of the stress protocol by a small incision in the tail. The mice were placed in their home cage after the protocol.

### Perfusion and Immunohistochemistry.

At the end of each protocol, animals were deeply anesthetized with 7% chloral hydrate (0.015ml/gr of body weight i.p.) and perfused transcardially with 30ml of PBS followed by 30ml of 10%-buffered formalin (Thermo Fisher Scientific, US). Brains were extracted and postfixed in 10%-buffered formalin overnight, cryoprotected in 30% sucrose solution, sectioned into 40µm coronal sections (three series) and stored at 4°C in PBS with sodium azide. Brain sections were washed three times (5 min each) and then blocked for 1 hour with 3% normal horse serum (diluted in 0.4% Triton X-100 in PBS). Sections were incubated in primary antibody overnight in the same solution as blocking, at room temperature with continuous agitation. Primary antibody used were rabbit anti VIP (ImmunoStar; 1:1000, #20077), Chicken anti-GFP (Invitrogen; 1:5,000, A10262), Rat anti-mCherry (Invitrogen; 1:4,000, M11217), Mouse anti-NeuN (Millipore; 1:3,000, MAB377). Then, the sections were washed six times (5 min each) in PBS, and incubated with secondary antibodies for 2 h in blocking solution at room temperature and continuous agitation. The secondary antibodies used were donkey anti rabbit antibody conjugated with CY5 fluorophore (Jackson Immuneresearch; 1:1000, # 711-175-152), Alexa fluor 488-conjugated Donkey anti-Chicken (Jackson; 1:200, AB2340375), Alexa fluor 555-conjugated Goat anti-Rat (Invitrogen; 1:200, A21434), Alexa fluor 488-conjugated Goat anti-Mouse (Jackson; 1:200, AB2338840). Then, sections were washed three times (10 min each) in PBS, mounted on electrostatically treated slides and coverslipped with VECTASHIELD Antifade Mounting Medium with DAPI (Vector Laboratories) for microscope visualization and image acquisition.

### RNA scope in situ hybridization.

Two brains from the *EnvA-ΔG-rabies-GFP* experiments were sectioned at 20µm and tissue was mounted on glass slides in RNAase-free conditions. Slices were dried and *in situ* hybridization was performed using RNAScope multiplex fluorescent reagent kit V2 (catalog #323100, Advanced Cell Diagnostics, US). Slices were pretreated with hydrogen peroxide for 10 minutes at room temperature and subjected to target retrieval step for 5 min in a steamer (>99°C), This was followed by dehydration in 90% alcohol and air-dried for 5 min. Sections were then treated with protease III at 40°C for 30 min. Then, the slices were rinsed with sterile water and incubated for 2 hrs either with the *Vglut2* probe (RNAscope Probe- Mm-Slc17a6; catalog #319171, Advanced Cell Diagnostics, US) or Vgat Probe (RNAscope Probe- Mm-Slc32a1; catalog # 319191, Advanced Cell Diagnostics, US) at 40°C for RNA hybridization. This is followed by incubation with amplification reagents, AMP1, AMP2 (30 min each) and AMP3 (15 min) at 40°C. Sections were then incubated with HRP -C1 for 15 mins and Cy5 fluorophore (catalog #NEL741001, PerkinElmer) for 30 mins at 40°C. Finally, HRP blocker was added to the sections for 15 min at 40°C. After each step sections were washed with 1 x wash buffer provided in the kit. Slides were then dried and coverslipped with Vectashield antifade mounting medium (catalog #H-1400, Vector Laboratories) for microscope visualization and image acquisition.

### Image acquisition, gradient maps and rabies transfected counting.

Coronal sections were scanned at 20X magnification using an Olympus VS120 slide-scanning microscope or at 63X magnification using a confocal microscope (Leica Stellaris 5). Fluorescence images were analyzed using Olympus OlyVIA software (3.4.1) or Leica application suite (version 4.2.1). The injection site of each animal was confirmed by the expression of mCherry or EGFP. Microphotographs were transformed to a gray scale and the background were subtracted. The images at the same anatomical level were superimposed with 30% transparency to build a gradient map using GIMP software (version 2.10.34). In the brain sections from the rabies experiment, we counted cell bodies in at least 3 levels for each nucleus (anterior, middle and posterior). In the sections used for *in situ* hybridization of rabies infected cells, we counted cell bodies and doubly-stained cells in 6 levels of the DMH. Cell body counting was conducted manually using the multipoint tool in Image J software (version 1.54). Because the number of infected cells varied considerably from animal to animal and we were not trying to establish the absolute number of cells that project to the PVH^CRH^ neurons, we did not attempt to correct the numbers for cell size.

### ChR2-assisted circuit mapping (CRACM).

For electrophysiology experiments, *AAV8-DIO- ChR2-mCherry* (~3–9 nL) was injected into the DMH of *Vglut2-ires-Cre::CRH-Venus* mice (*n*=4) or *Vgat-ires-Cre::CRH-Venus* mice (*n*=3) or *Vgat-ires-Cre::L10-GFP mice* (*n=5*) or into the cvPVH of *Vgat-ires-Cre::CRH-Venus* mice (*n*=7) or into the rDMH of *Vgat-ires- Cre::CRH-Venus* mice (*n*=4). Mice with AAV injections placed outside of the target were excluded from the analysis. Four to six weeks following the AAV injections, mice were deeply anaesthetized with isoflurane (5% in oxygen) via inhalation and transcardially perfused with ice-cold ACSF (N-methyl-D-glucamine, NMDG-based solution described below). The mouse brains were then quickly removed and sectioned coronally (250 µm- thickness) in ice-cold NMDG-based ACSF using a vibrating microtome (VT1200S, Leica). We first incubated the slices containing the PVH for 5 min at 37°C, then transferred them into a holding chamber at 37°C containing ACSF (Na-based solution) for 10 minutes. We let the brain slices gradually return to room temperature (~1 hour) before starting recording. Brain slices were recorded in a recording chamber, where were submerged, and perfused with Na-based ACSF (described below, 1–1.5 ml/min). We recorded PVH^CRH^ and cvPVH^Vgat^ neurons identified by Venus or L10-GFP (green) fluorescence, respectively, using a combination of fluorescence and infrared differential interference contrast microscopy (IR-DIC). Both the Venus-expressing CRH neurons and the L10-GFP neurons were numerous and clear under the microscopy without any enhancement protocol. We used a fixed stage upright microscope (BX51WI, Olympus America) equipped with a Nomarski water immersion lens (Olympus 40X / 0.8 NAW) and IR-sensitive CCD camera (ORCA-ER, Hamamatsu) to acquire real time images using Micro-Manager software. We recorded the neurons in whole-cell voltage clamp and current clamp configurations using a Multiclamp 700B amplifier (Molecular Devices), a Digidata 1322A interface, and Clampex 9.0 software (Molecular Devices). Neurons showing a greater than 10% change in input resistance over the duration of the recording were excluded from the analysis. We photostimulated the DMH or cvPVH axons in the PVH using a full-field (~10 mW/mm^2^, 1 mm beam width) 5W LUXEON blue light-emitting diode (470 nm wavelength; #M470L2- C4; Thorlabs), coupled to the epifluorescence pathway of the microscope. We stimulated photo-evoked excitatory postsynaptic currents (oEPSCs) or inhibitory postsynaptic currents (oIPSCs) with 10ms light pulses (0.1 Hz, for a minimum of 30 trials). In the DMH^Vgat^ injected mice, we also tested the effects of photostimulation on both the action potential firing of cvPVH^Vgat^ neurons and the spontaneous IPSC (sIPSCs) of PVH^CRH^ neurons. For sIPSC recordings, we used train stimulations with a duration of 60 seconds at frequencies of 5, 10, and 20 Hz, and a light pulse duration of 10 ms. For action potential recordings, we tested train stimulations of 10 and 60 seconds at the same frequencies (5, 10, and 20 Hz) with a light pulse duration of 10 ms. Action potential firing and oEPSCs (holding potential = −70 mV) were recorded in ACSF and using a K-gluconate-based pipette solution. oIPSCs (holding potential = 0 mV) were recorded in ACSF containing 1 mM kynurenic acid and using a Cs-methane-sulfonate-based pipette solution. To record photo-evoked synaptic events in the presence of synaptic blockade, we bath applied tetrodotoxin (TTX; 1 µM) and the potassium channel blocker, 4-AP (200–500 µM). For all recordings we added 0.5% biocytin in the pipette solutions to mark the recorded neurons. The recorded slices containing the PVH and cvPVH and the slices containing the DMH, where the AAVs were injected, were fixed overnight in 10%-buffered formalin for *post hoc* histological and anatomical assessment. To label and map the recorded neurons filled with biocytin, immediately after the *in vitro* recordings, we fixed the recorded slices in 10% buffered formalin, washed them, and incubated them overnight in streptavidin-conjugated Alexa Fluor 405 (1:500; Cat#: S32351; Invitrogen, Thermo Fisher Scientific Waltham, MA) ^54,55^. We acquired images using a Leica Stellaris 5 confocal microscope using a 63X oil immersion objective.

### Solutions for CRACM experiments.

NMDG-based ACSF solution containing (in mM): 100 NMDG, 2.5 KCl, 1.24 NaH_2_PO_4_, 30 NaHCO_3_, 25 glucose, 20 HEPES, 2 thiourea, 5 Na-L-ascorbate, 3 Na-pyruvate, 0.5 CaCl_2_, 10 MgSO_4_ (pH 7.3: 95% O_2_ and 5% CO_2_; 310–320 mOsm). Na-based ACSF solution contained (in mM): 120 NaCl, 2.5 KCl, 1.3 MgCl_2_, 10 glucose, 26 NaHCO_3_, 1.24 NaH_2_PO_4_, 4 CaCl_2_, 2 thiourea, 1 Na-L-ascorbate, 3 Na-pyruvate (pH 7.3–7.4 in 95% O_2_ and 5% CO_2_; 310–320 mOsm). Cs-methane-sulfonate- based pipette solution containing (in mM): 125 Cs-methane-sulfonate, 11 KCl, 10 HEPES, 0.1 CaCl_2_, 1 EGTA, 5 Mg-ATP and 0.3 Na-GTP (pH adjusted to 7.2 with CsOH, 280 mOsm). K-gluconate-based pipette solution containing (in mM): 120 K-Gluconate, 10 KCl, 3 MgCl2, 10 HEPES, 2.5 K-ATP, 0.5 Na-GTP (pH 7.2 adjusted with KOH; 280 mOsm). We purchased tetrodotoxin and kynurenic acid from Cayman Chemical (Ann Arbor, MI) and bicuculline methiodide from Tocris Bioscience (Ellisville, MO). We purchased all other chemicals from Fisher Scientific (Waltham, MA) or Sigma-Aldrich (Saint Luis, MO).

### Data and statistical analysis for CRACM experiments.

Recording data were analyzed using Clampfit 10 (Molecular Devices), MiniAnalysis 6 software (Synaptosoft), customized Python scripts (Python 3, www.python.org) and MatLab (version R2020B; MathWorks; Natick, MA) software. Figures were generated using Igor Pro version 6 (WaveMetrics), Prism 7 (GraphPad, La Jolla, CA), Inkscape (GitLab) and Photoshop (Adobe) software. To ensure unbiased detection of synaptic events, the EPSCs and IPSCs were detected and analyzed automatically using MiniAnalysis. We considered EPSCs or IPSCs in PVH^CRH^ neurons and cvPVH^Vgat^ neurons to be photo-evoked if their probability during the first 50 ms following the light pulses was greater than the IPSC probability + five times the standard error of the mean (SEM) before the light stimulation and if their latency was within ± 1 ms of the median value of E/IPSC latencies calculated for each neuron within the first 50 ms following photostimulation. We calculated the latency of the photo-evoked EPSC and IPSCs as the time difference between the start of the light pulse and the 5% rise point of the first synaptic event ^55^. Group means were compared using *paired* t-tests. Cumulative distributions were compared using the *Kolmogorov-Smirnov* test. Values indicating p < 0.05 were considered significant.

### Statistical analysis.

Data sets were tested to see whether they fulfilled the parametric criteria of Brown-Forsythe (homo/heteroscedasticity) and Shapiro Wilk (fit to normal distribution). Corticosterone plasma levels and circadian distribution of LMA and Tb were analyzed with a *two-way ANOVA* with a factor for group and a factor for time, followed by a *post-hoc* multiple comparisons *Sidak* test. CI for Cort was calculated by the difference between the mean ZT13 and ZT1 levels and divided by the ZT13-ZT1 mean in each mouse, then normalized to 100% by the mean difference in the Control group. CI for LMA and Tb were calculated by the difference between the mean dark (or presumptive dark) and light (or presumptive light) levels divided by the 24h total counts for LMA and 24h mean for Tb, then normalized to 100% against the mean for the Control group. Light or Dark mean for LMA and Tb were evaluated with *two-way ANOVA* with a factor for group and a factor for LD vs DD, followed by a *post-hoc* multiple comparisons *Tukey* test. When the same mice were evaluated under different conditions as their own controls for LMA and Tb (pre- and post-DTA experiments, and the hGlyR experiments), we evaluate them with *Repeated Measures [RM] two-way ANOVA* with a factor for group and a factor for LD vs DD, followed by a *post-hoc* multiple comparisons *Tukey* test. CI and amplitude data were compared by *unpaired* t-test analysis, or *one-way ANOVA*, followed by a *post-hoc* multiple comparisons *Tukey* test in the cases where included baseline in the analysis. Actograms, periodograms and amplitude of the cosinor fitting were built using ClockLab ActiMetrics software version 6.1.02. Statistical analyses and graphs were performed with the GraphPad Prism software version 8. Data are represented as mean ± SEM. Significance values α were set at p<0.05.

## Supplementary Material

1 *Extended figure 1. SPZ*^*Vgat*^ *neuron ablation flattens the circadian rhythm of LMA and reduces the amplitude of the rhythm of Tb.* (**a**) Representative micrograph of GABAergic neurons (native signal in green) from a *Vgat-ires-Cre::L10-GFP* control mouse (*left* panel), and the density plots of the distribution injections of AAV-mCherry-DIO-DTA in the SPZ of the *Vgat-ires-cre* mice (*n*=8; *right* panels). (**b**) Daily LMA was reduced during the dark period in LD after SPZ^*Vgat*^ ablation (*Repeated Measures [RM] Two-way ANOVA*; *Šídák’s* multiple comparisons test. SPZ^Vgat^-GFP vs SPZ^Vgat^-DTA: *p<0.05, ***p<0.001), and (**c**) the periodogram showed reduced amplitude (*Two-way ANOVA*; *Šídák’s* multiple comparisons test. SPZ^Vgat^-GFP vs SPZ^Vgat^-DTA: *p<0.05). (**d**) SPZ^Vgat^ neuron ablation further reduced the circadian rhythm of LMA in DD (RM *Two-way ANOVA*; *Šídák’s* multiple comparisons test. SPZ^Vgat^-GFP vs SPZ^Vgat^-DTA: *p<0.05), (**e**) causing a dramatical reduction on the amplitude of the periodogram (*Two-way ANOVA*; *Šídák’s* multiple comparisons test. SPZ^Vgat^-GFP vs SPZ^Vgat^-DTA: *p<0.05). (**f**) Bar graphs showing total LMA counts in the light and dark periods from the SPZ^Vgat^-GFP and SPZ^Vgat^-DTA mice. LMA was significantly reduced during the dark (*Two-way ANOVA*; *Tukey’s* multiple comparisons: SPZ^Vgat^-GFP dark vs SPZ^Vgat^-DTA dark = ***p<0.001) or subjective dark periods (*Two-way ANOVA*; *Tukey’s* multiple comparisons: SPZ^Vgat^-GFP subjective dark vs SPZ^Vgat^-DTA subjective dark = ***p<0.001). (**g**) The reduction of LMA during the dark or subjective dark period in SPZ^Vgat^-ablated mice reduced circadian index (CI, for method of calculation of CI, see Statistical Analysis in the Materials and Methods) by 51.1 ±3.5% in LD (*Unpaired t-test*: t=6.381, df=14, ***p<0.001) and by 79.7 ±4.8% in DD (*Unpaired t-test*: t=8.901, df=14, ***p<0.001), (**h**) while the cosinor amplitude was also reduced in LD (*Unpaired t-test*: t=6.142, df=14, ***p<0.001) and DD (*Unpaired t-test*: t=6.190, df=14, ***p<0.001) (**i**) Representative LMA actograms, showing LD and DD recordings from SPZ^Vgat^-GFP (blue) and SPZ^Vgat^-DTA (red) mice. (**j-n**) SPZ^Vgat^-ablated mice had a small increase in Tb during the light period and a small decrease during the dark period in both LD and DD, causing a significant change in the amplitude of the periodograms (LD: *Two-way ANOVA*; *Šídák’s* multiple comparisons test. SPZ^Vgat^-GFP vs SPZ^Vgat^-DTA: *p<0.05; DD: *Two-way ANOVA*; *Šídák’s* multiple comparisons test. SPZ^Vgat^-GFP vs SPZ^Vgat^-DTA: *p<0.05). However, (**o**) the CI was reduced by 50.4 ±5.6% in LD (*Unpaired t-test*: t=6.551, df=14, ***p<0.001) and by 58.3 ±6.5% in DD (*Unpaired t-test*: t=5.228, df=14, ***p<0.001), and (**p**) the cosinor amplitude of Tb was also reduced both in LD (Paired t-test: t=6.199, df=14, ***p<0.001) and DD (*Unpaired t-test*: t=4.885, df=14, ***p<0.001). (**q**) Representative Tb actograms, red and blue lines as in panel **i**. LD, Light:Dark photoperiod; DD, Constant darkness.
*Extended figure 2. Vgat gene deletion from SPZ neurons only partially reduces LMA and Tb during the dark period.* (**a**) Representative micrograph of *Vgat* mRNA expression (in red) from a control mouse (*left* panel) and an iCre-EGFP injected mouse (in green; *right* panel). Notice the elimination of *Vgat mRNA* expression in the area where iCre-EGFP was expressed, whereas the *Vgat* expression in the SCN was preserved. The EGFP signal was enhanced with immunofluorescence for EGFP. Density plots of the distribution of injections of AAV-EGFP-iCre in the SPZ of the *Vgat* ^*loxP/loxP*^ mice (*n*=7; *right* panels). (**b**) Neither daily total LMA nor (**c**) the LMA periodogram was affected by the *Vgat* deletion in the SPZ when animals were in LD, but (**d**) LMA was reduced during the subjective dark period in DD (RM *Two-way ANOVA*; *Šídák’s* multiple comparisons test. SPZ^Vgat/flox^-Control vs SPZ^Vgat/flox^-EGFP-iCre: *p<0.05), (**e**) with a reduction of the amplitude in the periodogram (*Two-way ANOVA*; *Šídák’s* multiple comparisons test. SPZ^Vgat/flox^-Control vs SPZ^Vgat/flox^-EGFP-iCre: *p<0.05). (**f**) Total LMA counts in the light and dark periods from the SPZ^Vgat/flox^-Control and SPZ^Vgat/flox^-EGFP-iCre mice. *Vgat* ablation from SPZ neurons reduced the LMA during the subjective dark period under DD (*Two-way ANOVA*; *Tukey’s* multiple comparisons: SPZ^Vgat/flox^-Control subjective dark vs SPZ^Vgat/flox^- EGFP-iCre subjective dark = **p=0.004), (**g**) resulting in a reduced circadian index (CI) by 39.7 ±4.8% in DD (*Unpaired t-test*: t=4.674, df=10, ***p<0.001), (**h**) and a similar reduction the cosinor amplitude in DD (*Unpaired t-test*: t=9.399, df=12, ***p<0.001) (**i**) Representative LMA actograms, showing LD and DD recordings from SPZ^Vgat/flox^-Control (blue) and SPZ^Vgat/flox^- EGFP-iCre (red). (**j**) The Vgat deletion from SPZ slightly reduced the Tb toward the end of the dark period in LD *(RM Two-way ANOVA*; Šídák’s multiple comparisons test. SPZ^Vgat/flox^-Control vs SPZ^Vgat/flox^-EGFP-iCre: *p<0.05), (**k**) reducing the amplitude in the periodogram (*Two-way ANOVA*; *Šídák’s* multiple comparisons test. SPZ^Vgat/flox^-Control vs SPZ^Vgat/flox^- EGFP-iCre: *p<0.05). (**l**) Similar reduction was observed during the subjective dark period in DD in the SPZ^Vgat/flox^-EGFP-iCre mice *(RM Two-way ANOVA*; Šídák’s multiple comparisons test. SPZ^Vgat/flox^-Control vs SPZ^Vgat/flox^-EGFP-iCre: *p<0.05) (**m**) and in the periodogram (*Two-way ANOVA*; *Šídák’s* multiple comparisons test. SPZ^Vgat/flox^- Control vs SPZ^Vgat/flox^-EGFP-iCre: *p<0.05). (**n**) The mean Tb was significantly reduced during the dark or subjective dark period in the SPZ^Vgat/flox^-EGFP-iCre mice either in LD (*Two-way ANOVA*; *Tukey’s* multiple comparisons: SPZ^Vgat/flox^-Control vs SPZ^Vgat/flox^-EGFP- iCre dark = *p=0.021) or DD, (*Two-way ANOVA*; *Tukey’s* multiple comparisons: SPZ^Vgat/flox^- Control subjective dark vs SPZ^Vgat/flox^-EGFP-iCre subjective dark = ***p<0.001). (**o**) The CI of Tb was reduced by 25.7 ±4.6% in LD (*Unpaired t-test:* t=3.300, df=12, ***p=0.006) and by 38.6 ±7.3% in DD (*Unpaired t-test*: t=4.689, df=12, ***p<0.001), (**p**) similar to the cosinor amplitude in LD (*Unpaired t-test*: t=2.864, df=12, **p=0.014) and DD (*Unpaired t- test*: t=5.318, df=12, ***p<0.001). (**q**) Representative Tb actograms, red and blue lines as in panel **i**.
*Extended figure 3. DMH*^*Vglut2*^ *neuron ablation reduces LMA during the subjective night and decreases Tb across the day.* (**a**) Density plots of the distribution of injections of AAV- mCherry-DIO-DTA in the DMH of the *Vglut2-ires-cre* mice (*n*=8). (**b**) No statistically significant change was detected in the Cort levels after the restraint stress protocol. (**c**) Daily LMA was reduced during the dark period in LD after DMH^Vglut2^ neuron ablation (RM *Two-way ANOVA*; *Šídák’s* multiple comparisons test. Pre-DTA vs DMH^Vglut2^-DTA: *p<0.05), but (**d**) the periodogram showed no change. (**e**) DMH^Vglut2^ neuron ablation reduced LMA during the subjective dark in DD (RM *Two-way ANOVA*; *Šídák’s* multiple comparisons test. Pre-DTA vs DMH^Vglut2^-DTA: *p<0.05), (**f**) reducing the amplitude of the periodogram (*Two-way ANOVA*; *Šídák’s* multiple comparisons test. SPZ^Vgat^-GFP vs SPZ^Vgat^-DTA: *p<0.05). (**g**) Bar graphs showing total LMA counts in the light and dark periods before and after ablation of DMH^Vglut2^ neurons, during LD (left) and presumptive light and dark periods in DD (right). (**h**) The reduction of LMA during the dark period after DTA was sufficiently small that the circadian index (CI) was not significantly different. (**i**) However, the amplitude of the circadian rhythm of LMA as measured by cosinor analysis was reduced in DD after ablation (*Paired t-test*: t=3.611, df=14, **p=0.002). (**j**) Representative LMA actograms, showing LD and DD recordings before (blue) and after (red) DTA. (k) Tb was reduced across the light phase and in the middle of the dark phase after DMH^Vglut2^ neuron ablation in LD (*Repeated Measures [RM] Two-way ANOVA*; Šídák’s multiple comparisons test. Pre-DTA vs DMH^Vglut2^-DTA: *p<0.05), while (**l**) the periodogram showed no change. (**m**) The reduction in the daily Tb was larger in both the presumptive light and dark periods in DD after DMH^Vglut2^ neuron ablation (RM *Two-way ANOVA*; *Šídák’s* multiple comparisons test. Pre-DTA vs DMH^Vglut2^-DTA: *p<0.05), (**n**) with no change in the periodogram. (**o**) The mean Tb was significantly reduced by DMH^Vglut2^ ablation for the light but not the dark period in LD, and for both in DD (*Two-way ANOVA*; *Tukey’s* multiple comparisons. ** p<0.01, ***p<0.001). However, (**p**) the CI and (**q**) the cosinor amplitude of Tb was increased after ablation (by 46.65 ±12.8%) only during LD (*Paired t-test for CI*: t=3.075, df=14, df=14, *p=0.008; *for cosinor amplitude*: t=2.415, df=14, *p=0.03). (**r**) Representative Tb actograms, red and blue lines as in panel **j**. LD, Light:Dark photoperiod; DD, Constant darkness.
*Extended figure 4. Vglut2 gene deletion from DMH neurons reduces the peak of LMA and Tb during the transition from the dark to the light period.* (**a**) Density plots of the injections of AAV-EGFP-iCre in the DMH of Vglut2^loxP/loxP^ mice (*n*=7). (**b**) Daily distribution of LMA in LD (*RM Two-way ANOVA*; Šídák’s multiple comparisons test. DMH^Vglut2/flox^-Control vs DMH^Vglut2/flox^-EGFP-iCre: *p<0.05). *Vglut2* gene deletion from DMH neurons reduced LMA during the transition from the dark to the light period, and to a lesser extend in the early dark period, (**c**) reducing the amplitude of the periodogram peak at 24h (*Two-way ANOVA*; *Šídák’s* multiple comparisons test. DMH^Vglut2/flox^-Control vs DMH^Vglut2/flox^-EGFP-iCre: *p<0.05). (**d**) In DD, the reduction in LMA during the transitions between the presumptive light and dark periods was similar to LD (RM *Two-way ANOVA*; *Šídák’s* multiple comparisons test. DMH^Vglut2/flox^-Control vs DMH^Vglut2/flox^-EGFP-iCre: *p<0.05), (**e**) with similar reduction in the amplitude of the periodogram (*Two-way ANOVA*; *Šídák’s* multiple comparisons test. DMH^Vglut2/flox^-Control vs DMH^Vglut2/flox^-EGFP-iCre: *p<0.05). (**f**) However, for the entire light and dark periods in LD and presumptive light and dark periods in DD, the small changes in LMA did not reach statistical significance for total LMA counts, (**g**) CI or (**h**) cosinor amplitude. (**i**) Representative LMA actograms from the Control and DMH *Vglut2* gene*-*deleted mice. (**j**) Tb was also reduced in LD during the transition from the dark to the light phase and to a lesser extent in the early dark phase in DMH *Vglut2* gene- deleted mice (RM *Two-way ANOVA*; *Šídák’s* multiple comparisons test. DMH^Vglut2/flox^- Control vs DMH^Vglut2/flox^-EGFP-iCre: *p<0.05), (**k**) reducing the amplitude in the periodogram around the 24 period (*Two-way ANOVA*; *Šídák’s* multiple comparisons test. DMH^Vglut2/flox^-Control vs DMH^Vglut2/flox^-EGFP-iCre: *p<0.05). (**l**) A similar pattern in Tb was seen during DD (RM *Two-way ANOVA*; *Šídák’s* multiple comparisons test. DMH^Vglut2/flox^- Control vs DMH^Vglut2/flox^-EGFP-iCre: *p<0.05), (**m**) with similar reduction in the amplitude of the periodogram (*Two-way ANOVA*; *Šídák’s* multiple comparisons test. DMH^Vglut2/flox^- Control vs DMH^Vglut2/flox^-EGFP-iCre: *p<0.05). (**n**) However, as for LMA, these changes were not large enough to produce a statistically significant difference in mean Tb, (**o**) CI or (**p**) cosinor amplitude of the circadian rhythm of Tb. (**q**) Tb actograms from the DMH *Vglut2* gene-deleted mice included in this study. LD, Light:Dark photoperiod; DD, Constant darkness.
*Extended figure 5. Chemogenetic inhibition of the DMH*^*Vglut2*^ *neurons decreases the circadian index of LMA and reduces Tb.* (**a**) Density plots of the distribution of injections of AAV-DIO-hGlyR-mCherry in the DMH of the *Vglut2-ires-Cre* mice (*n*=5). (**b-d**) Chemo- inhibitions with IVM in LD induced higher LMA at the transition from the dark to the light phase and reduced the peak during the first hours of the dark phase (**b**: *RM Two-way ANOVA*; *Tukey’s* multiple comparisons test p<0.05. Line above the 24h graphs represent significative differences in Baseline vs IVM in green, and VEH vs IVM in blue; **c**: *Two-way ANOVA*; *Tukey’s* multiple comparisons test. light Baseline vs light IVM: **p=0.001, light VEH vs light IVM: *p=0.042, dark Baseline vs dark IVM: *p=0.03), reducing the CI (**d**: *One- way ANOVA*; *Tukey’s* multiple comparisons test. Baseline vs IVM: **p=0.001, VEH vs IVM: *p=0.016). (**e-g**) In DD, the daily increase in LMA at the beginning of the dark period was reduced (**e**: *RM Two-way ANOVA*; *Tukey’s* multiple comparisons test p<0.05. Lines above same as in B. **f**: *Two-way ANOVA*; *Tukey’s* multiple comparisons test. dark Baseline vs dark IVM: *p=0.036), reducing also the LMA CI (**g**: *One-way ANOVA*; *Tukey’s* multiple comparisons test. Baseline vs IVM: ***p<0.001, VEH vs IVM: *p=0.01). (**h-m**) Tb during the dark and presumptive dark period was reduced between 24–48 hr after IVM (**h** and **k**: *RM Two-way ANOVA*; *Tukey’s* multiple comparisons test p<0.05. Line above same as in B. I: *Two-way ANOVA*; *Tukey’s* multiple comparisons test. dark Baseline vs dark IVM: *p=0.01, dark VEH vs dark IVM: *p=0.024. **l**: *Two-way ANOVA*; *Tukey’s* multiple comparisons test. dark Baseline vs dark IVM: *p=0.01, dark VEH vs dark IVM: *p=0.014) resulting in a roughly 50% decrease in the CI of Tb during this same time period in LD and DD, although this only reached statistical significance in LD (*One-way ANOVA*; *Tukey’s* multiple comparisons test. Baseline vs IVM: *p=0.015, VEH vs IVM: *p=0.024).
*Extended figure 6. Presumed monosynaptic inputs to the PVH*^*CRH*^ *neurons based on conditional rabies virus tracing.* (**a**) Schematic of the rabies infection of PVH^CRH^ neurons to map their inputs. (**b**) Expression of both TVA (in red) and rabies EnvA (in green) marks doubly-transfected PVH^CRH^ neurons (yellow) as “starter cells” to which neurons labeled only with green are presumed to project. (**c**) Representative micrographs of areas with retrogradely labeled neurons. (**d**) Total counts of the EnvA-rabies transfected neurons through the brain. The hypothalamus represents the most important source of inputs to the PVH^CRH^ neurons. (**e**) Schematic of the EnvA-rabies experiment to map the monosynaptic input from the DMH^Vgat^ neurons to PVH^CRH^ neurons. (**f**) Mapping of the rabies-*Vgat* co- labeling distribution in the DMH at different rostro-caudal levels (*left* panel), and representative images showing *Vgat mRNA* expression (in magenta, f’ and f’’) and rabies expression (in green; f’ and f’’’) within the DMH (*right* panels). Neurons without Vgat mRNA (presumably glutamatergic) are shown by arrowheads and a doubly labeled cell indicated by the arrow is shown in a magnified inset at the lower right of each panel. The green signal from the Rabies infected cells was enhanced with immunofluorescence for EGFP. Reference scale bar: in **c** = 200µm, in **b and f’-f’’’**= 50µm, in **f’-f’’’** insets = 10µm. 3V, third ventricle; AC, Anterior commissure; F, Fornix; OC, Optic Chiasm; SCP, Superior Cerebellar Peduncle; AHA, Anterior Hypothalamic Area; Arc, Arcuate Nucleus; BNST, Bed Nucleus of the Stria Terminalis; DMH, Dorsomedial Hypothalamus; LH, Lateral Hypothalamus; LPB, Lateral Parabrachial; LPO, Lateral Preoptic Area; MnPO, Median Preoptic Nucleus; MPA, Medial Preoptic Area; NTS, Nucleus of the Tractus Solitarius; PAG, Periaqueductal Gray Area; PE, Periventricular hypothalamic nucleus; POA, Preoptic Area; PVH, Paraventricular Hypothalamic nucleus ; RCh, Retrochiasmatic Nucleus; SCN, Suprachiasmatic Nucleus; SFO, Subfornical organ; SON, Supraoptic Nucleus; SPZ, Subparaventricular Zone; VLPO, Ventrolateral Preoptic Area; VMH, Ventromedial Hypothalamus; VMPO, Ventromedial Preoptic Area; VOLT, Vascular Organ of Lamina Terminalis; VP, Ventral Pallidum. Neuroanatomical regions and names were based on the Paxinos & Franklin Atlas ^47^.
*Extended figure 7.* Ablation of *DMH*^*Vgat*^ *neurons dramatically reduces the total amount and circadian rhythm of LMA, but only reduces the daily level of Tb with little effect on its circadian rhythm.* (**a**) Density plots of the distribution of injections of AAV-mCherry-DIO- DTA in the DMH of Vgat-ires-cre mice (*n*=8). (**b**) Magnification of the representative micrograph showed in Fig 5b, showing few if any remaining Vgat-expressing neurons (green, native signal) within the area of the injection site (red, native signal). (**c**) The Cort levels were similar before and after the DMH^Vgat^ ablation in the restraint stress protocol. (**d-j**) LMA was reduced during the dark phase after DMH^Vgat^ neuron ablation in both LD (*RM Two-way ANOVA*; *Šídák’s* multiple comparisons test. Pre-DTA vs DMH^Vgat^-DTA: *p<0.05) and DD (*RM Two-way ANOVA*; *Šídák’s* multiple comparisons test. Pre-DTA vs DMH^Vgat^- DTA: *p<0.05), resulting in a reduced circadian index by 38.23 ±9.2% in LD (Paired t-test: t=3.252, df=14, ***p=0.005) and by 50.61 ±12.9% in DD (*Paired t-test*: t=3.605, df=14, ***p=0.003) and similar reductions in cosinor amplitude in LD (*Paired t-test*: t=5.138, df=14, ***p<0.001) and DD (*Paired t-test*: t=5.503, df=14, ***p<0.001), with a reduction in peak amplitude but no change in tau in the periodogram (LD and DD: *Two-way ANOVA*; *Šídák’s* multiple comparisons test. SPZ^Vgat^-GFP vs SPZ^Vgat^-DTA: *p<0.05). (**k**) Representative LMA actograms before and after DMH^Vgat^ neuron ablation (blue lines represents pre-ablation and red lines is post-ablation). (**l**) Tb was reduced by about 0.3^o^ C in LD, but (**m**) there was no change in the amplitude or period of the rhythm in periodogram analysis. (**n**) In DD, only the Tb during the presumptive dark period was reduced, but (**o**) this reduced the amplitude of the mean periodogram peak at 24h (*Two- way ANOVA*; *Šídák’s* multiple comparisons test. SPZ^Vgat^-GFP vs SPZ^Vgat^-DTA: *p<0.05). (**p**) The reduction in mean Tb during the dark period is statistically significant in both LD and DD (LD: *Two-way ANOVA*; *Tukey’s* multiple comparisons. Dark Pre-DTA vs DMH^Vgat^- DTA: *p=0.032; DD: Dark Pre-DTA vs DMH^Vgat^-DTA: *p=0.012), but (**q-r**) there is no significant difference in the CI or cosinor amplitude of Tb. (**s**) Representative Tb actograms before and after DMH^Vgat^ neuron ablation.
*Extended figure 8. Vgat gene deletion in DMH neurons reduces the elevation of LMA and Tb during the middle of the dark and presumptive dark periods and the amplitude of their circadian rhythms.* (**a**) Density plots of the injections of AAV-EGFP-iCre in the DMH of *Vgat*^*loxP/loxP*^ mice (*n*=7). (**b-e**) *Vgat* gene deletion in the DMH caused lower LMA during the middle of the dark phase in LD and presumptive dark phase in DD (LD: *RM Two-way ANOVA*; *Šídák’s* multiple comparisons test. DMH^Vgat/flox^-Control vs DMH^Vgat/flox^-EGFP-iCre: *p<0.05; DD: *RM Two-way ANOVA*; *Šídák’s* multiple comparisons test. DMH^Vgat/flox^-Control vs DMH^Vgat/flox^-EGFP-iCre: *p<0.05) with a reduction in the amplitude of in the mean periodogram peak at 24h (LD: *Two-way ANOVA*; *Šídák’s* multiple comparisons test. DMH^Vgat/flox^-Control vs DMH^Vgat/flox^-EGFP-iCre: *p<0.05; DD: *Two-way ANOVA*; *Šídák’s* multiple comparisons test. DMH^Vgat/flox^-Control vs DMH^Vgat/flox^-EGFP-iCre: *p<0.05). (**f**) Deletion of the *Vgat* gene in the DMH reduced movement during the dark or subjective dark period (*Two-way ANOVA*; *Tukey’s* multiple comparisons test. LD DMH^Vgat/flox^-Control vs DMH^Vgat/flox^-EGFP-iCre: ***p<0.001; DD Dark DMH^Vgat/flox^-Control vs DMH^Vgat/flox^-EGFP- iCre: ***p<0.001). (**g**) The CI of LMA rhythm was reduced by 25.73 ±6.01% in LD (*Unpaired* t-test t=4.275 df=12, **p=0.001) and 33.35 ± 12.63% in DD (*Unpaired* t-test t=2.642 df=12, *p=0.021) and (**h**) the cosinor amplitude of LMA was similarly reduced in LD (*Unpaired* t-test: t=5.194, df=12, ***p<0.001) and DD (*Unpaired* t-test: t=5.232, df=12, ***p<0.001). (**i**) Representative LMA actograms from AAV-DIO-GFP and AAV-DIO-EGFP- iCre injected mice. (**j-m**) Tb is reduced during the middle of the dark phase in DMH *Vgat* gene-deleted mice in LD (*RM Two-way ANOVA*; *Šídák’s* multiple comparisons test. DMH^Vgat/flox^-Control vs DMH^Vgat/flox^-EGFP-iCre: *p<0.05) and the presumptive dark phase in DD (*RM Two-way ANOVA*; *Šídák’s* multiple comparisons test. DMH^Vgat/flox^-Control vs DMH^Vgat/flox^-EGFP-iCre: *p<0.05), with no change in the mean periodogram. (**n**) The reduction in mean Tb during the dark and presumptive dark phase in the DMH *Vgat*-gene deleted mice was statistically significant (*Two-way ANOVA*; *Tukey’s* multiple comparisons test. LD Dark DMH^Vgat/flox^-Control vs DMH^Vgat/flox^-EGFP-iCre: **p=0.001; DD Dark DMH^Vgat/flox^-Control vs DMH^Vgat/flox^-EGFP-iCre: ***p<0.001). (**o**) As a result, the CI of Tb was also reduced in the DMH *Vgat*-gene deleted mice by 20.84 ±8.8% in LD (*Unpaired* t- test t=2.342 df=12, *p=0.037) and by 26.6 ±8.9% under constant dark (*Unpaired* t-test t=2.958 df=12, *p=0.012), and (**p**) the cosinor amplitude was lower in DMH *Vgat* gene- deleted mice in LD (*Unpaired* t-test: t=2.482, df=12, *p= 0.028) and DD (*Unpaired* t-test: t=3.902, df=12, **p=0.002). (**q**) Representative Tb actograms from AAV-DIO-GFP and AAV-DIO-EGFP-iCre injected mice.
*Extended figure 9. Chemogenetic inhibition of DMH*^*Vgat*^ *neurons flattens the circadian rhythm of LMA and Tb.* (**a**) Density plots of the AAV-DIO-hGlyR-mCherry injection sites in the DMH of *Vgat-ires-Cre* mice (*n*=5). (**b-g**) There was a reduction in the amount of LMA during the dark and presumptive dark periods between 24–48 hr after IVM injection (**b** and **e**: *RM Two-way ANOVA*; *Tukey’s* multiple comparisons test p<0.05. Line above the 24h graphs represent significative differences in Baseline vs IVM in green, and VEH vs IVM in blue; **c**: *Two-way ANOVA*; *Tukey’s* multiple comparisons test. dark Baseline vs dark IVM: ***p<0.001, dark VEH vs dark IVM: **p=0.001. F: *Two-way ANOVA*; *Tukey’s* multiple comparisons test. dark VEH vs dark IVM: *p=0.04), resulting in a dramatic reduction in CI during the same time period (**d**: *One-way ANOVA*; *Tukey’s* multiple comparisons test. Baseline vs IVM: ***p<0.001, VEH vs IVM: **p=0.001. **g**: *One-way ANOVA*; *Tukey’s* multiple comparisons test. Baseline vs IVM: ***p<0.001, VEH vs IVM: **p=0.002). (**h-m**) Reductions in mean Tb after IVM administration were observed during the dark and subjective dark periods (**h** and **k**: *RM Two-way ANOVA*; *Tukey’s* multiple comparisons test p<0.05. **i**: *Two-way ANOVA*; *Tukey’s* multiple comparisons test. dark Baseline vs dark IVM: *p=0.041. **l**: *Two-way ANOVA*; *Tukey’s* multiple comparisons test. dark Baseline vs dark IVM: *p=0.038), driving a reduction in CI in both LD and DD (**j**: *One-way ANOVA*; *Tukey’s* multiple comparisons test. Baseline vs IVM: ***p<0.001, VEH vs IVM: **p=0.005. **m**: *One-way ANOVA*; *Tukey’s* multiple comparisons test. Baseline vs IVM: ***p<0.001, VEH vs IVM: **p=0.004).
*Extended figure 10. cvPVH*^*Vgat*^ *neuron ablation increases the Cort response to restraint stress, but does not affect the circadian rhythm of LMA or Tb.* (**a**) Density plots showing the distribution of injections of AAV-mCherry-DIO-DTA in the cvPVH of Vgat-ires-Cre mice (*n*=5). (**b**) After 1 hour of movement restraint, mice with cvPVH^Vgat^ ablation had higher levels of Cort (Unpaired t test: t=2.782, df=8, p= 0.047). (**c**) The 24h LMA counts and (**d**) mean periodogram under LD, as well as under DD (**e, f**) did not differ from control mice. (**g-i**) As a result, no differences were detected in the circadian rhythms of LMA after cvPVH^Vgat^ neuron ablation (**j**) Representative LMA actograms from both controls and mice with ablation of cvPVH^Vgat^ neurons. (**k-n**) There were no significant changes in mean Tb levels or their periodograms under either LD or DD. (**o-q**) Likewise, the circadian rhythm of Tb was undisturbed by the cvPVH^Vgat^ ablation. (**r**) Representative Tb actograms from Vgat- Cre animals with injections of AAV-DIO-GFP and AAV-mCherrry-DIO-DTA the cvPVH. LD, Light:Dark photoperiod; DD, Constant darkness.
*Extended Figure 11. In vitro optogenetic stimulation of the GABAergic input from the caudal DMH inhibits PVH*^*CRH*^ *neurons.* (**a**) A schematic of the experiment demonstrating connectivity between the caudal DMH (cDMH) Vgat neurons and ipsilateral PVH^CRH^ neurons (cDMH^Vgat^ → PVH^CRH^; the DMH is shown on the opposite side of the brain to ease illustration). *Vgat-ires-Cre::CRH-Venus* mice were injected with *AAV-DIO-ChR2-mCherry* in the cDMH, and recordings were conducted in brain slices from Venus-labeled PVH^CRH^ neurons while photostimulating the cDMH^Vgat^ input. (**b**) An example of ChR2-mCherry expression in the cDMH (*top left*, native signal) and density plots of the *AAV-DIO-ChR2- mCherry* injection sites (*n* = 4 mice; *right* and *bottom*). (**c**) Opto-evoked inhibitory post- synaptic currents (oIPSCs) recorded in the PVH^CRH^ neurons. (**d**) Percentages of PVH^CRH^ neurons responding (Connected) and not responding (Not Connected) to photostimulation of the cDMH^Vgat^ input (*n*= 23 PVH^CRH^ recorded neurons from 4 mice). (**e**) Amplitude (*left*; filled markers, cells responding to photostimulation, *n*=22, open markers, cells not responding to photostimulation, *n*=1 neurons, from 4 mice; mean and ± SEM of responding neurons) and latency (*right*) of oIPSCs in PVH^CRH^ neurons in response to photostimulation of the cDMH^Vgat^ input (mean and ± SEM; *n*=22 from 4 mice). (**f**) Raster plot of IPSCs in a representative PVH^CRH^ neurons with photostimulation of the cDMH^Vgat^ → PVH^CRH^ input (bin duration: 50ms). (**g**) IPSC probability in response to photostimulation of the cDMH^Vgat^ → PVH^CRH^ input (black, *n* = 23). Reference scale bar: in (**b**) = 250 µm. f, fornix; 3V, third ventricle.
