# Supplementary material for: A hypothalamic circuit for circadian regulation of corticosterone secretion
Source: Res Sq. 2025 Jun 18:rs.3.rs-4718850. Originally published 2024 Jul 12. Preprint. [Version 2] doi: 10.21203/rs.3.rs-4718850/v2 (PMC11261983; doi:10.21203/rs.3.rs-4718850/v2)
Supplement: 1 — Extended figure 1. SPZVgat neuron ablation flattens the circadian rhythm of LMA and reduces the amplitude of the rhythm of Tb. (a) Representative micrograph of GABAergic neurons (native signal in green) from a Vgat-ires-Cre::L10-GFP control mouse (left panel), and the density plots of the distribution injections of AAV-mCherry-DIO-DTA in the SPZ of the Vgat-ires-cre mice (n=8; right panels). (b) Daily LMA was reduced during the dark period in LD after SPZVgat ablation (Repeated Measures [RM] Two-way ANOVA; Šídák’s multiple comparisons test. SPZVgat-GFP vs SPZVgat-DTA: *p<0.05, ***p<0.001), and (c) the periodogram showed reduced amplitude (Two-way ANOVA; Šídák’s multiple comparisons test. SPZVgat-GFP vs SPZVgat-DTA: *p<0.05). (d) SPZVgat neuron ablation further reduced the circadian rhythm of LMA in DD (RM Two-way ANOVA; Šídák’s multiple comparisons test. SPZVgat-GFP vs SPZVgat-DTA: *p<0.05), (e) causing a dramatical reduction on the amplitude of the periodogram (Two-way ANOVA; Šídák’s multiple comparisons test. SPZVgat-GFP vs SPZVgat-DTA: *p<0.05). (f) Bar graphs showing total LMA counts in the light and dark periods from the SPZVgat-GFP and SPZVgat-DTA mice. LMA was significantly reduced during the dark (Two-way ANOVA; Tukey’s multiple comparisons: SPZVgat-GFP dark vs SPZVgat-DTA dark = ***p<0.001) or subjective dark periods (Two-way ANOVA; Tukey’s multiple comparisons: SPZVgat-GFP subjective dark vs SPZVgat-DTA subjective dark = ***p<0.001). (g) The reduction of LMA during the dark or subjective dark period in SPZVgat-ablated mice reduced circadian index (CI, for method of calculation of CI, see Statistical Analysis in the Materials and Methods) by 51.1 ±3.5% in LD (Unpaired t-test: t=6.381, df=14, ***p<0.001) and by 79.7 ±4.8% in DD (Unpaired t-test: t=8.901, df=14, ***p<0.001), (h) while the cosinor amplitude was also reduced in LD (Unpaired t-test: t=6.142, df=14, ***p<0.001) and DD (Unpaired t-test: t=6.190, df=14, ***p<0.001) (i) Representative LMA actograms, [file NIHPPrs4718850v2-supplement-1.pdf]

Supplementary Files

This is a list of supplementary files associated with this preprint. Click to download.

- FigS1SPZDTA.tif
- FigS2SPZFlFl.tif
- FigS3VglutDMHDTA.tif
- FigS4VglutDMHFl.tif
- FigS5VglutDMHhGlyR.tif
- FigS6CRHRabiesnew1k.tif
- FigS7VgatDMHDTAopt2.tif
- FigS8VgatDMHFl.tif
- FigS9VgatDMHhGlyR.tif
- FigS10VgatPVHDTA.tif
- FigS11DMHVGATcaudalPVHCRH.tif
